# A randomized, double-blind, phase 2b study to investigate the efficacy, safety, tolerability and pharmacokinetics of a single-dose regimen of ferroquine with artefenomel in adults and children with uncomplicated *Plasmodium falciparum* malaria

**DOI:** 10.1186/s12936-021-03749-4

**Published:** 2021-05-19

**Authors:** Yeka Adoke, Rella Zoleko-Manego, Serge Ouoba, Alfred B. Tiono, Grace Kaguthi, Juvêncio Eduardo Bonzela, Tran Thanh Duong, Alain Nahum, Marielle Bouyou-Akotet, Bernhards Ogutu, Alphonse Ouedraogo, Fiona Macintyre, Andreas Jessel, Bart Laurijssens, Mohammed H. Cherkaoui-Rbati, Cathy Cantalloube, Anne Claire Marrast, Raphaël Bejuit, David White, Timothy N. C. Wells, Florian Wartha, Didier Leroy, Afizi Kibuuka, Ghyslain Mombo-Ngoma, Daouda Ouattara, Irène Mugenya, Bui Quang Phuc, Francis Bohissou, Denise P. Mawili-Mboumba, Fredrick Olewe, Issiaka Soulama, Halidou Tinto, the FALCI Study Group, Michael Ramharter, Diolinda Nahum, Hermione Zohou, Irène Nzwili, John Michael Ongecha, Ricardo Thompson, John Kiwalabye, Amidou Diarra, Aboubacar S. Coulibaly, Edith C. Bougouma, Désiré G. Kargougou, Moubarak Tegneri, Catherine Castin Vuillerme, Elhadj Djeriou, Aziz Filali Ansary

**Affiliations:** 1 Infectious Diseases Research Collaboration (IDRC), Nakasero Hill Road, P.O. Box 7475, Kampala, Uganda; 2 Centre de Recherches Médicales de Lambaréné (CERMEL), BP 142, Lambaréné, Gabon; 3 Department of Tropical Medicine, Bernhard Nocht Institute for Tropical Medicine & I. Dep. of Medicine, University Medical Centre Hamburg-Eppendorf, Bernhard Nocht Straße 74, 20359 Hamburg, Germany; 4 Unité de Recherche Clinique de Nanoro, Institut de Recherche en Sciences de La Santé, Nanoro, Burkina Faso; 5 Unité de Recherche Clinique de Banfora, C/H Régional de Banfora, Centre National de Recherche Et de Formation Sur Le Paludisme (CNRFP), Ouagadougou, Province de la Comoé Burkina Faso; 6 Kenya Medical Research Institute-Centre for Respiratory Diseases Research (KEMRI-CRDR), P.O. Box 47855-00100, Nairobi, Kenya; 7 Chokwe Health Research and Training Centre, 1 Bairro, Zona Do Orli, CP 30, Chokwe, Mozambique; 8 National Institute of Malariology, Parasitology and Entomology, Hanoi, Vietnam; 9 Centre de Recherche Entomologique de Cotonou (CREC) and Hôpital La Croix de Zinvié, 06 BP 2604 Cotonou, Benin; 10 Département de Parasitologie-Mycologie-Médecine Tropicale, Faculté de Médecine Et Des Sciences de La Santé, Université Des Sciences de La Santé, BP 4009 Libreville, Gabon; 11 Kenya Medical Research Institute, Kisumu, Kenya; 12 Unité de Recherche Clinique de Niangoloko, S/C Centre Medical de Niangoloko, Centre National de Recherche Et de Formation Sur Le Paludisme (CNRFP), BP 37, Niangoloko, Burkina Faso; 13 Medicines for Malaria Venture (MMV), Route de Pré-Bois 20, Post Box 1826, 1215 Geneva 15, Switzerland; 14 Sanofi Research and Development, 55 Corporate Drive, Bridgewater, NJ 08807 USA; 15 BEL Pharm Consulting, 116 Chemin du Moulin d’Ozil, 07140 Chambonas, France; 16 Sanofi Research and Development, 1 avenue Pierre Brossolette, 91385 Chilly-Mazarin, France; 17 IQVIA CEVA, 4820 Emperor Boulevard, Durham, NC 27703 USA; 18 Institute for Tropical Medicine, University of Tübingen, Wilhelmstraße 27, 72074 Tübingen, Germany

**Keywords:** Ferroquine, Combination treatment, Pharmacokinetics/pharmacodynamics, Exposure–response, C580Y, *Kelch-13* mutation, Resistance, Parasite clearance, Vomiting

## Abstract

**Background:**

For uncomplicated *Plasmodium falciparum* malaria, highly efficacious single-dose treatments are expected to increase compliance and improve treatment outcomes, and thereby may slow the development of resistance. The efficacy and safety of a single-dose combination of artefenomel (800 mg) plus ferroquine (400/600/900/1200 mg doses) for the treatment of uncomplicated *P. falciparum* malaria were evaluated in Africa (focusing on children ≤ 5 years) and Asia.

**Methods:**

The study was a randomized, double-blind, single-dose, multi-arm clinical trial in patients aged > 6 months to < 70 years, from six African countries and Vietnam. Patients were followed up for 63 days to assess treatment efficacy, safety and pharmacokinetics. The primary efficacy endpoint was the polymerase chain reaction (PCR)-adjusted adequate clinical and parasitological response (ACPR) at Day 28 in the Per-Protocol [PP] Set comprising only African patients ≤ 5 years. The exposure–response relationship for PCR-adjusted ACPR at Day 28 and prevalence of *kelch-13* mutations were explored.

**Results:**

A total of 373 patients were treated: 289 African patients ≤ 5 years (77.5%), 64 African patients > 5 years and 20 Asian patients. None of the treatment arms met the target efficacy criterion for PCR-adjusted ACPR at Day 28 (lower limit of 95% confidence interval [CI] > 90%). PCR-adjusted ACPR at Day 28 [95% CI] in the PP Set ranged from 78.4% [64.7; 88.7%] to 91.7% [81.6; 97.2%] for the 400 mg to 1200 mg ferroquine dose. Efficacy rates were low in Vietnamese patients, ranging from 20 to 40%. A clear relationship was found between drug exposure (artefenomel and ferroquine concentrations at Day 7) and efficacy (primary endpoint), with higher concentrations of both drugs resulting in higher efficacy. Six distinct *kelch-13* mutations were detected in parasite isolates from 10/272 African patients (with 2 mutations known to be associated with artemisinin resistance) and 18/20 Asian patients (all C580Y mutation). Vomiting within 6 h of initial artefenomel administration was common (24.6%) and associated with lower drug exposures.

**Conclusion:**

The efficacy of artefenomel/ferroquine combination was suboptimal in African children aged ≤ 5 years, the population of interest, and vomiting most likely had a negative impact on efficacy.

*Trial registration* ClinicalTrials.gov, NCT02497612. Registered 14 Jul 2015, https://clinicaltrials.gov/ct2/show/NCT02497612?term=NCT02497612&draw=2&rank=1

**Supplementary Information:**

The online version contains supplementary material available at 10.1186/s12936-021-03749-4.

## Background

Malaria is one of the leading public health problems in the world with an estimated 228 million cases and 400,000 deaths in 2018. About 93% of the cases occurred in Africa and 67% of deaths occurred in children under 5 years. *Plasmodium falciparum* is the most prevalent malaria parasite in Africa, accounting for 99.7% of estimated malaria cases in 2018, while it represented 50% of the cases in South-East Asia [1].

To counter the threat of plasmodial resistance to anti-malarial monotherapies and to improve treatment outcomes, the World Health Organization (WHO) recommends the use of artemisinin-based combination therapy (ACT) as the first-line treatment for uncomplicated *P. falciparum* malaria [2]. However, evidence suggests that in real-life conditions, compliance to current standard 3-day treatment regimens recommended by the WHO can be low, reducing the effectiveness of ACT [3–5]. In children aged 5 years or less, adherence may be as low as 40% [6], although reported data are highly variable [6–10], as are adherence data in other age groups [5].

Poor compliance, leading to sub-optimal treatment outcomes, is likely to increase the rate of development of drug resistance. Parasite resistance against commonly used artemisinin-based combinations is rising in South-East Asia [11–14] and the potential to spread to African countries is a major concern. *Kelch-13* mutations associated with artemisinin resistance (delayed parasite clearance) are now widespread in the Greater Mekong Region (GMR), with C580Y representing the most prevalent mutation in South-East Asia [15]. There is clear evidence of malaria treatment failure in the face of emerging drug resistance in the region [13, 16–19].

Although the efficacy of ACT has remained high outside South-East Asia [20, 21], *kelch-13* mutations have been detected at a significant prevalence (over 5%) in Guyana, Papua New Guinea and Rwanda [1], and slow parasite clearance has been observed at a frequency of < 1% in Africa [22]. Recently, *kelch-13* C580Y has been detected in Guyana [23] and Papua New Guinea [24]. For uncomplicated *P. falciparum* malaria, highly efficacious single-dose treatments are expected to increase compliance and improve treatment outcomes, and thereby may slow the development of resistance [25].

A new fixed-dose combination of ferroquine (IND 115 244) and artefenomel (IND 104 549), which have different mechanisms of action, was developed by Sanofi in partnership with Medicines for Malaria Venture (MMV) for the single-dose treatment of uncomplicated malaria in adults and children. Artefenomel, previously known as OZ439, is a synthetic trioxolane endoperoxidic anti-malarial agent [26, 27] which, like artemisinins, has multiple mechanisms of action including reacting with iron within the parasite food vacuole to produce free radicals, leading to alkylation of key parasitic proteins [26, 28, 29]. Artefenomel was selected for development based on improved blood stability and an extended plasma half-life relative to artemisinins in rat [28]. In patients with falciparum and vivax malaria, artefenomel had an estimated elimination half-life of 46 to 62 h and demonstrated rapid parasite clearance [27]. In a previous phase 2b study in African adults and children, and Asian adults, a single dose of artefenomel (800 mg) administered in combination with 1440 mg of piperaquine phosphate provided a mean Day 28 efficacy (polymerase chain reaction [PCR]-adjusted adequate clinical and parasitological response [ACPR]) of 78.6% in the Per-Protocol (PP) Set [30].

Ferroquine is a ferrocenyl derivative of chloroquine that is active against chloroquine-resistant *P. falciparum* strains with a promising anti-malarial therapeutic potential in humans [31, 32]. It has been proposed to act by preventing haemozoin formation generating reactive oxygen species and inducing lipid peroxidation [29, 31]. Ferroquine is metabolized to one major metabolite (N-demethyl derivative, SSR97213), equally active in vitro.

Based on data from a Controlled Human Infection Model (CHMI) in healthy volunteers, time above the minimum parasiticidal concentration was estimated to be approximately twofold longer for a ferroquine dose of 1200 mg than for a piperaquine dose of 1440 mg (*MMV Internal Report*), suggesting that ferroquine in combination with artefenomel may have longer duration than piperaquine in combination with artefenomel.

A phase 2b study was conducted to investigate the efficacy, safety, tolerability and pharmacokinetics (PK) of single-dose regimens of ferroquine with artefenomel for the treatment of uncomplicated *P. falciparum* malaria in adults and children in Africa and Asia.

## Methods

### Study objectives

The primary objective of the FALCI Study (for 'Ferroquine and Artefenomel in adults and children with *Plasmodium falciparum* malaria') was to determine the efficacy of a single-dose combination of artefenomel/ferroquine for the treatment of uncomplicated *P.* *falciparum* malaria in adults and children.

Key secondary efficacy objectives included determining the time to re-emergence of parasites, and time to fever and parasite clearance. The safety and tolerability of artefenomel/ferroquine in adults and children, and the PK of artefenomel, ferroquine and its active metabolite, SSR97213, were also evaluated. Key exploratory objectives included investigating the relationship between *kelch-13* genotype and parasite clearance, and characterizing the relationship between the exposure of both drugs and the clinical outcome.

### Study design and overview of study conduct

This was a multi-centre, multi-country, randomized, double-­blind, single-dose, multi-arm study to evaluate 4 dosing regimens in patients aged > 6 months to < 70 years (body weight ≥ 5 kg to ≤ 90 kg) with uncomplicated *P. falciparum* malaria. The study was conducted at 10 sites across 6 African countries (Benin, Burkina Faso, Gabon, Kenya, Mozambique and Uganda), and 4 sites in Vietnam (2 of which did not randomize patients).

The primary population of interest for this study was African patients aged > 6 months to ≤ 5 years (later referred to as African patients ≤ 5 years). Two other populations were recruited: African patients > 5 years who were primarily included to allow a safe age step-down procedure, and Asian patients. Recruitment of Asian patients was stopped by an independent Data Monitoring Committee (DMC) following evidence of low efficacy in this population.

The study design was adaptive, with up to four pre-planned interim analyses of efficacy response. The study was stopped after all treatment arms met the protocol-defined futility criteria at the first interim analysis (*see result section*). The protocol and statistical analysis plan for this study are available on ClinicalTrials.gov.

### Study participants

Patients were eligible if they presented with microscopically-confirmed (blood smear) *P. falciparum* mono-infection (1000 to 100 000 asexual parasites/μL) and fever (axillary temperature ≥ 37.5 °C, or oral/rectal/tympanic temperature ≥ 38 °C, or documented history of fever in the previous 24 h). Patients with signs of severe malaria (according to the WHO definition [33]), severe malnutrition, or hepatic function abnormalities (alanine aminotransferase [ALT] or aspartate aminotransferase [AST] > 2 × upper limit of normal [ULN], or total bilirubin > 1.5 × ULN) were excluded.

### Enrolment and randomization to study treatment

Adults and children were included sequentially in 4 cohorts through a progressive age step-down procedure and ferroquine dose step-up procedure. Following the review of safety data of the first cohort (> 14 years to < 70 years) by the independent DMC, sequentially younger patients were recruited.

Eligible patients were centrally randomized to one of the possible treatment arms (artefenomel/ferroquine 800/400 mg, 800/600 mg, 800/900 mg, and 800/1200 mg) via an Integrated Web Recognition System (IWRS) using permuted block randomization schedules and 3 randomization lists. Randomization was stratified by region, and within Africa, by age class (> 14 to < 70 years; > 5 to ≥ 14 years; > 2 to ≤ 5 years; > 6 months to ≤ 2 years). Following the decision to stop recruitment in Asia due to low efficacy, stratification by region was no longer relevant after the second cohort (> 5 to ≥ 14 years). Following the age step-down and ferroquine dose step-up procedures, the randomization across treatment arms was balanced.

Patients weighing < 35 kg received doses that were adjusted to body weight. Ferroquine doses were selected using a population PK model, such that the lightest subject of a given weight band would not have exposures higher than the lightest adult (35 kg). Artefenomel doses were selected such that the heaviest patient in each weight band would achieve similar exposures to a 60 kg adult, and the lightest patient would not exceed the exposures of the lightest adult (35 kg) in the same treatment arm. A fixed artefenomel/ferroquine dose ratio was to be maintained within each weight band. For further details on enrolment procedures, allocation ratios, justification of the doses of ferroquine and artefenomel, and dose adjustment to body weight, please refer to Additional file 1.

### Administration of study treatments, methods of blinding and handling of vomiting

Exploratory formulations of both ferroquine and artefenomel were administered orally in the fasting condition (3 h before and 2 h after completion of administration) by a healthcare worker. Ferroquine capsules were administered first, in a double-blinded manner, meaning that patients in each weight band received the same number of capsules (6 or 8) depending on the dose, followed immediately by artefenomel suspension (unblinded). Ferroquine capsules could be opened and a solution prepared for young children. The total administration volume for children from 2 to 5 years of age was between 110 and 180 mL, depending on weight.

If patients vomited during or after ferroquine administration but before artefenomel administration, no re-dosing of ferroquine was to be performed. Instead, the patients received rescue treatment and were discontinued from the study (but were followed-up for safety). Patients who vomited within 5 min of the start of artefenomel administration were to be re-dosed. Patients who vomited from 5 min after the start of artefenomel administration continued to take the artefenomel dose (if there was any left), but were not to be re-dosed.

For further details on administration of study treatments, please refer to Additional file 1.

### Baseline and follow-up assessments

Dosing was performed at Day 0. Patients were hospitalized for a minimum of 48 h post-dose (African patients > 5 years of age) or 72 h post-dose (all Asian patients and African patients ≤ 5 years of age). Following discharge from the hospital, patients returned for further assessments at Days 3, 5, 7, 10, 14, 15–18, 21 (± 2 days), 24–25, 28 (± 2 days), 42 (± 3 days) and 63 (± 3 days).

#### Determination of parasitaemia by microscopy

Blood films (2 thick films and 1 thin film) were prepared at screening/pre-dose, and post-dose after 6, 12, 18, 24, 30, 36, 48, 72 h and at Days 5, 7, 10, 14, (15–18), 21, (24–25), 28, 42 and 63. In addition, blood films at 12 h and 30 h post-dose were taken in patients > 14 years and ≥ 35 kg only. For screening/pre-dose (taken within 4 h before drug administration), the first thick blood film was stained with 10% Giemsa stain for 10 to 15 min to determine screening parasitaemia. The second thick film, processed only if the patient met the entry criteria, used a more accurate staining technique with 2.5 to 3% Giemsa stain for 45 to 60 min, to determine the pre-dose (baseline) parasitaemia. Subsequent thick and thin films were stained using this more accurate staining technique. Two qualified microscopists independently read the stained thick and thin films. Asexual parasite density, expressed as the number of parasites per microliter of blood, was calculated by averaging the 2 counts. Non-concordance was resolved by a third microscopist. A slide was considered negative where no asexual parasites were detected in 1000 counted leukocytes. A negative result was confirmed by a second negative film, prepared within 6 to 12 h of the first). Gametocyte numbers were also counted.

#### Polymerase chain reaction (PCR) methodology

Parasite genotyping by PCR (blood spots) was performed centrally in accordance with the procedures to identify parasite populations recommended by the WHO and MMV [34].

Parasite clearance parameters were estimated using the WorldWide Antimalarial Resistance Network (WWARN) parasite clearance estimator based on the linear portion of the individual natural logarithm parasitaemia time profiles [35].

#### Exploratory assessments

*Kelch-13* genotyping was determined by the PCR method developed by the Pasteur Institute (MOL08) [36].

#### Safety assessments

Safety assessments were performed until study completion, unless otherwise specified. This included recording of adverse events (AEs), clinical laboratory tests up to Day 28 (haematology, clinical chemistry, urinalysis), physical examination, vital signs, clinical signs and symptoms related to uncomplicated *P. falciparum* malaria, as well as a triplicate 12-lead electrocardiogram (ECG) using centralized reading up to Day 7. Adverse events were followed-up until the scheduled date of the patient's study completion, or up to resolution or stabilization of the AE, whichever came last.

### Ethical considerations

The study was conducted in accordance with consensus ethics principles derived from international ethics guidelines, including the Declaration of Helsinki, and the International Council for Harmonisation of Technical Requirements for Pharmaceuticals for Human Use guidelines for Good Clinical Practice, all applicable laws, rules, and regulations. It was approved by the relevant Independent Ethics Committees (IECs) and, where relevant, local regulatory authorities at each of the participating sites.

### Analysis sets

The Safety Set included all randomized patients who received at least one dose or part of a dose of the study drug. Three Safety Subsets (African patients ≤ 5 years, African patients > 5 years and Asian patients) were also defined.

The primary population of interest was African patients ≤ 5 years, and the modified Intent-To-Treat (mITT) and PP Sets included only this population. The mITT Set included all randomized African patients ≤ 5 years with parasitologically confirmed *P. falciparum* malaria at screening/baseline, who received the single dose of artefenomel plus ferroquine, and excluded patients who required rescue treatment due to vomiting during study drug administration. The PP Set comprised the mITT patients who were evaluable at Day 28, Day 42 or Day 63 for crude ACPR (see definition below) and who were without any major protocol deviation affecting efficacy up to the specified study day. Separate PP Sets and mITT Sets were defined for African patients > 5 years and Asian patients.

Data were analysed according to the study treatment received (all patients received treatment as randomized).

### Endpoints

The primary efficacy endpoint was the PCR-adjusted ACPR at Day 28 and the primary analysis population was the PP Set (African patients ≤ 5 years). Secondary endpoints included PCR-adjusted ACPR at Day 42 and Day 63 in the PP Set and crude ACPR at Day 28, Day 42 and Day 63 in the mITT Set. ACPR was defined as a negative parasitaemia (blood films), irrespective of axillary temperature, without previously meeting any criteria of early treatment failure, late clinical failure, or late parasitological failure, or having received rescue treatment for malaria under the conditions defined in the study protocol.

PCR-adjusted ACPR and crude ACPR for both the mITT and PP Sets were determined according to the principles set down by the WHO and MMV [34, 37]. Crude ACPR does not distinguish between recrudescence (re-emergence of the original clone of parasite that was present at baseline) and re-infection (by a new clone of parasite). For PCR-adjusted ACPR, re-infection on the day of evaluation was considered a cure for both mITT and PP Sets, while re-infection before the day of evaluation was considered a failure for the mITT Set and non-evaluable for the PP Set.

Additional secondary and exploratory endpoints included Kaplan–Meier estimates of time to, and incidence rates of re-emergence, recrudescence and re-infection (mITT Sets), Kaplan–Meier estimates of parasite clearance time (PCT) and fever clearance time (FCT) (PP Sets), parasite clearance parameters (including parasite clearance half-life [PCt1/2], parasite reduction ratio at 24 h and 48 h [PRR24 and PRR48 (log10)], time to 90% parasite reduction) (PP Sets), gametocyte count and Kaplan–Meier estimates of time to gametocyte appearance/clearance (mITT Sets), and *kelch-13* genotype of parasite isolates (PP and mITT Sets).

Safety and tolerability endpoints included the incidence of treatment-emergent adverse events (TEAEs) and serious adverse events (SAEs). Treatment-emergent adverse events of special interest (AESIs) were pre-defined for the study: increase in ALT (ALT ≥ 3 × ULN if baseline ALT < ULN, or ALT ≥ 2 × baseline value if baseline ALT ≥ ULN); QT interval corrected using Fridericia's formula (QTcF) ≥ 500 ms or QTcF prolongation > 60 ms from baseline; pregnancy and follow-up; symptomatic overdose with the study drug. Cases of QTcB prolongation > 500 ms were also thoroughly reviewed by an independent cardiologist. Other safety endpoints included physical examination findings, vital signs, clinical laboratory tests including liver function tests, and ECG abnormalities (including absolute QTc value categorization and change from baseline QTc).

### Statistical considerations

The aim of the study was to determine whether any of the treatment arms reached a target PCR-adjusted ACPR at Day 28 of > 90% (PP Set, i.e. African patients ≤ 5 years). The study was not powered for comparison between treatment arms.

#### Sample size calculations

Trial simulations for different PCR-adjusted ACPR response rates at Day 28 showed that a sample size of 150 evaluable patients per treatment arm would provide ≥ 80% probability to reject the null hypothesis (H0: probability of PCR-adjusted ACPR at Day 28 ≤ 0.90) at the final analysis, for the true rate of 96.4%. Recruitment was to continue until each treatment arm was deemed to be futile or until 150 evaluable patients per treatment arm was reached.

At the start of the study, the primary population of interest included Asian patients of all ages as well as African patients ≤ 5 years of age. Following evidence of low efficacy in the Asian population, recruitment of Asian patients was stopped, as per the DMC's guidance, and the primary population of interest was redefined as African children > 6 months and ≤ 5 years. In this new scenario, where no dose regimen was dropped during the study, a maximum of approximately 662 African patients (62 patients > 5 years, plus approximately 150 patients ≤ 5 years per dose group) were to be recruited and randomized to achieve approximately 600 African patients ≤ 5 years of age evaluable for the primary efficacy endpoint. A total of 21 Asian patients had already been recruited.

#### Statistical analyses of the primary efficacy endpoint

PCR-adjusted ACPR at Day 28 in the PP Set (African patients ≤ 5 years) was analysed by treatment arm, using a frequency table including the exact binomial two-sided 95% confidence interval (CIs) of the percentages calculated using the Clopper-Pearson method. Subgroup analyses were performed according to age, body weight, baseline parasitaemia and country.

Interim analyses of the primary efficacy endpoint: The study design was adaptive, with pre-planned interim analyses of the response during study conduct, in the first instance after recruiting 50 evaluable patients (African patients ≤ 5 years) per arm. Futility was to be concluded if the probability that PCR-adjusted ACPR at Day 28 was ≤ 90% was ≥ 0.3 [38]. Further interim analyses were planned (up to 3) every time another 25 patients per treatment arm had completed the Day 28 assessment. Asian patients and African patients > 5 years were excluded from the interim efficacy/futility analyses.

Final analysis of the primary efficacy endpoint: Efficacy of the treatment arm was demonstrated for African patients ≤ 5 years (PP Set) if the lower limit of the exact 95% CI of PCR-adjusted ACPR rate at Day 28 was > 90%. Within each treatment arm, the null hypothesis (H0: p ≤ 0.90) was tested against the one-sided alternative (H1: p > 0.90, with a two-sided α level of 5%), where p was the probability of PCR-adjusted ACPR at Day 28. Where the lower bound of the exact (Clopper-Pearson) 95% two-sided CI was > 0.9, then the null hypothesis was to be rejected.

#### Statistical analyses of other endpoints

Summary frequency tables with exact binomial two-sided 95% CIs of the percentages were produced for PCR-adjusted ACPR at Days 42 and 63 (PP Set), and for crude ACPR at Days 28, 42 and 63 (mITT Set). Kaplan–Meier estimates were provided with the median (and 95% CI) and quartiles. Descriptive summary statistics were produced for all secondary and exploratory endpoints. The efficacy analyses performed in the primary population of interest (African patients ≤ 5 years) were repeated for the African patients > 5 years and the Asian patients, as secondary analyses. The relationship between *kelch-13* genotype and PCt1/2 was described by region (all African patients and Asian patients separately) and by mutation in the PP Sets, and summary statistics were reported.

### Pharmacokinetic analysis

Samples for PK analysis of artefenomel in plasma and ferroquine/SSR97213 in blood (dried blood spot) were collected at a total of 16 time points for each patient in patients > 14 years and body weight ≥ 35 kg (11 for artefenomel and 13 for ferroquine/SSR97213). In the younger patients, the number of samples collected was between 4 and 7 samples per patient. The samples were analysed using validated methods and the lower limit of quantification was 5 ng/mL for ferroquine/SSR97213 and 1 ng/mL for artefenomel.

The PK analysis was performed using non-linear mixed effect modelling as implemented in Monolix version 2019R1 [39]. All data processing, analysis, model setup and modelling result analysis were conducted within R (Microsoft Open R 3.5.1) combined with the IQR tools package (v1.1.1) developed by IntiQuan [40].

To obtain the individual patient exposure estimates for artefenomel and ferroquine/SSR97213 with minimum bias due to sample time, individual PK profiles were estimated through Bayesian analysis applying previously developed population PK models (artefenomel [30], ferroquine [unpublished]; details in Additional file 3). Exposure variables included maximum concentration (C_max_), concentrations at Day 7 post-dose (C_day7_), area under the curve from time 0 to Day 28 (AUC_(0-day28)_) for ferroquine and SSR97213, and area under the curve from time 0 to infinity (AUC_(0-inf)_) for artefenomel. More details are provided in *S3 Pharmacokinetic analysis details* (see Additional file 3).

### Exploratory efficacy exposure–response analysis

The relationship between the binary outcome of PCR-adjusted ACPR at Day 28 and the estimated C_day7_ of both artefenomel and ferroquine and other covariates was evaluated in a logistic regression model within R combined with the IQRtools package as described above [40]. The following covariates were considered: baseline parasitaemia, estimated C_day7_ of the active ferroquine metabolite SSR97213, age, sex, region, study site and *kelch-13* genotype at screening. All patients with a valid estimate of the PCR-adjusted ACPR at Day 28 and an estimated exposure to both drugs were included in the analysis. More details are provided in *S4 Exposure–response analysis details* (see Additional file 4).

### Exploratory ECG exposure–response analysis

The relationships between the changes from baseline in ECG parameters and ferroquine/artefenomel concentrations from pre-dose to 48 h post-dose were explored. All patients with valid ECG recordings and exposure to both drugs were included in the analysis. More details are provided in *S5 Electrocardiogram exposure–response analysis details* (see Additional file 5).

## Results

### Interim analysis results

All treatment arms in the PP Set (African children ≤ 5 years of age) met the protocol-defined futility criteria at the first interim analysis (n = 226), given that the probability that efficacy (PCR-adjusted ACPR at Day 28) was ≤ 90% was too large (≥ 30%) for all doses (30.7% for the 1200 mg ferroquine dose). Recruitment was therefore stopped.

### Final analysis results

#### Patient disposition and analysis sets

**Fig. 1:**
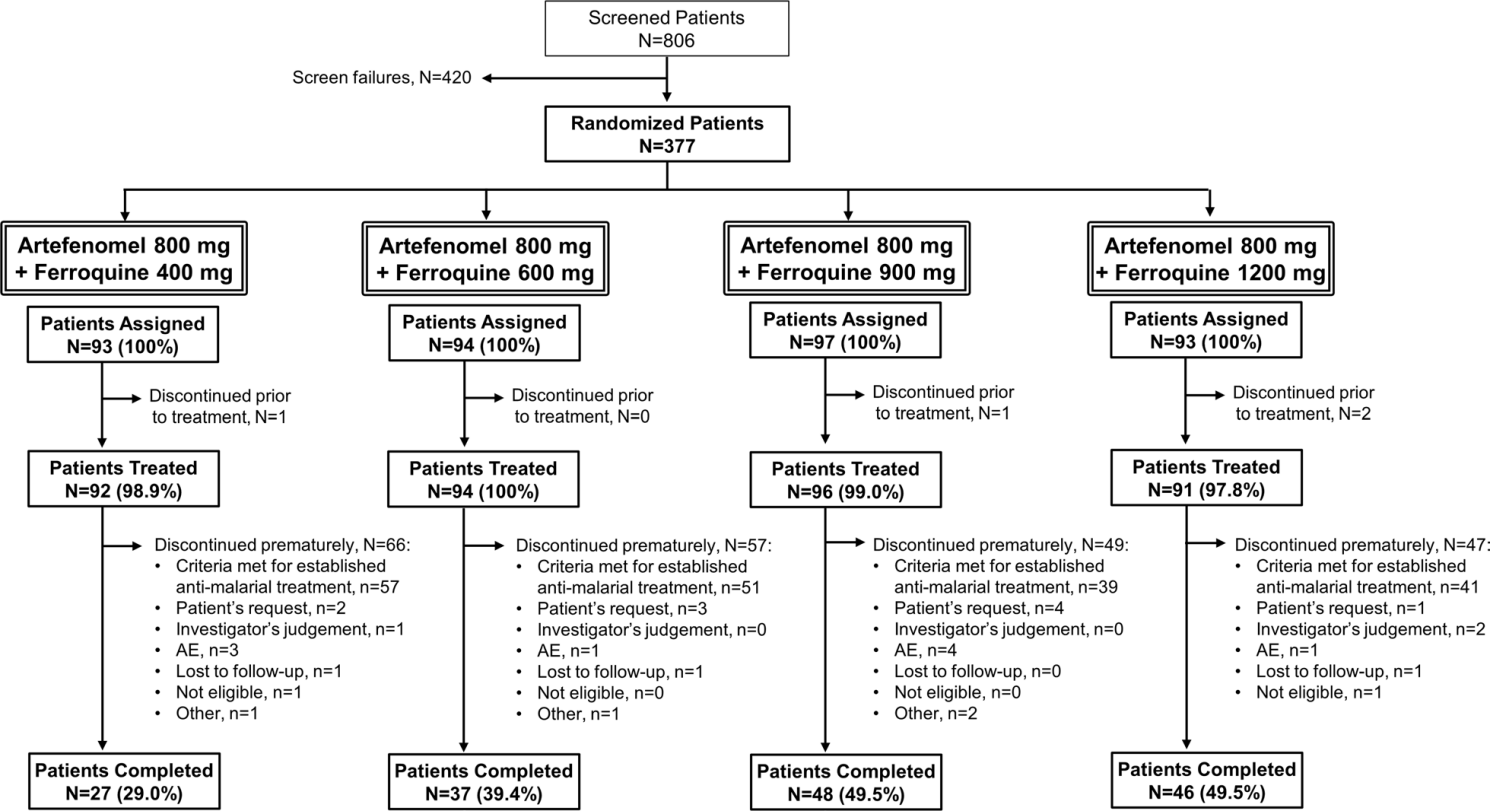
Patient disposition by treatment arm. All doses of artefenomel and ferroquine are expressed as adult-equivalent doses

Patient recruitment and follow-up occurred between 25 July 2015 and 23 September 2019. Patient disposition of the study population is presented by treatment arm in Fig. 1. Of the 806 patients screened for the study, 377 were randomized to receive study treatment. Screen failures (n = 420, 52.1%) were mainly due to the patients not meeting the criterion ‘mono-infection by *P. falciparum*’ (25.4%) and/or the patients presenting with clinically significant laboratory abnormalities (12.3%). Four patients were randomized in error and were discontinued before receiving study treatment. Of the 377 randomized patients, 373 patients (98.9%) received study treatment (Safety Set) and 158 patients (41.9%) completed the study up to Day 63. A total of 219 patients (58.1%) were prematurely discontinued from the study during the follow-up period up to Day 63, the majority of whom (188/219) discontinued prematurely due to meeting at least 1 of the criteria to receive established anti-malarial treatment due to treatment failure (49.9% of the 377 randomized patients) (see Additional File 1 for treatment failure criteria). Premature discontinuation due to treatment failure was therefore linked with the efficacy endpoint ACPR (Fig. 1).

Patient disposition is presented by region in Table 1. A total of 289 African patients ≤ 5 years (77.5% of all treated patients) and 64 African patients > 5 years were randomized and treated across all dose regimens. Only 20 Asian patients were randomized and treated, due to a decision based on DMC recommendation to stop recruitment in Asia early due to low efficacy. The percentage of patients who completed the study up to Day 63 was 44.9% of African patients ≤ 5 years, 35.9% of African patients > 5 years, and 19.0% of Asian patients (Table 1).

**Table 1:** Patient disposition by region (randomized patients)

	Artefenomel mg: Ferroquine mg				
	800:400	800:600	800:900	800:1200	Total
African patients > 6 months and ≤ 5 years (Primary population of interest)					
Randomized	73 (100)	74 (100)	73 (100)	72 (100)	292 (100)
Treated	72 (98.6)	74 (100)	72 (98.6)	71 (98.6)	289 (99.0)
Discontinued prior to treatment	1 (1.4)	0		1 (1.4)	3 (1.0)
Completed	25 (34.2)	32 (43.2)	38 (52.1)	36 (50.0)	131 (44.9)
Discontinued prematurely:	48 (65.8)	42 (56.8)	35 (47.9)	36 (50.0)	161 (55.1)
*Criteria met for established anti-malarial treatment*^a^	*44 (60.3)*	*38 (51.4)*	*29 (39.7)*	*32 (44.4)*	*143 (49.0)*
*Patient's request*	*0*	*1 (1.4)*	*1 (1.4)*	*0*	*2 (0.7)*
*Investigator's judgement*	*0*	*0*	*0*	*1 (1.4)*	*1 (0.3)*
*AE*	*3 (4.1)*	*1 (1.4)*	*3 (4.1)*	*1 (1.4)*	*8 (2.7)*
*Lost to follow-up*	*0*	*1 (1.4)*	*0*	*1 (1.4)*	*2 (0.7)*
*Not eligible*^b^	*1 (1.4)*	*0*	*0*	*1 (1.4)*	*2 (0.7)*
*Other*	*0*	*1 (1.4)*	*2 (2.7)*	*0*	*3 (1.0)*
African patients > 5 years					
Randomized	16 (100)	15 (100)	18 (100)	15 (100)	64 (100)
Treated	16 (100)	15 (100)	18 (100)	15 (100)	64 (100)
Discontinued prior to treatment	0	0	0	0	0
Completed	2 (12.5)	4 (26.7)	8 (44.4)	9 (60.0)	23 (35.9)
Discontinued prematurely:	14 (87.5)	11 (73.3)	10 (55.6)	6 (40.0)	41 (64.1)
*Criteria met for established anti-malarial treatment*^a^	*10 (62.5)*	*9 (60.0)*	*7 (38.9)*	*5 (33.3)*	*31 (48.4)*
*Patient's request*	*1 (6.3)*	*2 (13.3)*	*2 (11.1)*	*1 (6.7)*	*6 (9.4)*
*Investigator's judgement*	*1 (6.3)*	*0*	*0*	*0*	*1 (1.6)*
*AE*	*0*	*0*	*1 (5.6)*	*0*	*1 (1.6)*
*Lost to follow-up*	*1 (6.3)*	*0*	*0*	*0*	*1 (1.6)*
*Other*	*1 (6.3)*	*0*	*0*	*0*	*1 (1.6)*
Asian patients					
Randomized	4 (100)	5 (100)	6 (100)	6 (100)	21 (100)
Treated	4 (100)	5 (100)	6 (100)	5 (83.3)	20 (95.2)
Discontinued prior to treatment	0	0	0	1 (16.7)	1 (4.8)
Completed	0	1 (20.0)	2 (33.3)	1 (16.7)	4 (19.0)
Discontinued prematurely:	4 (100)	4 (80.0)	4 (66.7)	5 (83.3)	17 (81.0)
*Criteria met for established anti-malarial treatment *^***a***^	*3 (75.0)*	*4 (80.0)*	*3 (50.0)*	*4 (66.7)*	*14 (66.7)*
*Patient's request*	*1 (25.0)*	*0*	*1 (16.7)*	*0*	*2 (9.5)*
*Investigator's judgement*	*0*	*0*	*0*	*1 (16.7)*	

Treatment failure was defined as patients who met any of the criteria for early treatment failure, late clinical failure or late parasitological failure. *Early treatment failure* (Day 1 to 3) was any of the following: (i) danger signs or severe malaria at Day 1, 2 or 3 in the presence of parasitaemia; (ii) parasite count at Day 2 higher than at Day 0, irrespective of axillary temperature; (iii) parasitaemia at Day 3 with axillary temperature ≥ 37.5 °C; (iv) parasite count at Day 3 ≥ 25% at Day 0. *Late clinical failure* (Day 4 to 63) was any of the following: (i) danger signs or severe malaria in the presence of parasitaemia on any day between Day 4 and Day 63 in patients who did not previously meet any of the criteria of early treatment failure; (ii) presence of parasitaemia on any day between Day 4 and Day 63 with axillary temperature ≥ 37.5 °C (or history of fever) in patients who did not previously meet any of the criteria of early treatment failure. *Late parasitological failure* was defined as the presence of parasitaemia on any day between Day 7 and Day 63 and axillary temperature < 37.5 °C in patients who did not previously meet any of the criteria of early treatment failure or late clinical failure

Data presented are the number (%) of randomized patients. Items in italic represent the reasons for premature study discontinuation. All doses of artefenomel and ferroquine are expressed as adult-equivalent doses

^b^Two patients were randomized in error and not treated. The status at last study contact was missing

^a^Established anti-malarial treatment was recommended as rescue treatment in case of (i) vomiting during or after ferroquine dosing or vomiting within 35 min after artefenomel re-dosing, and (ii) treatment failure

**Fig. 2:**
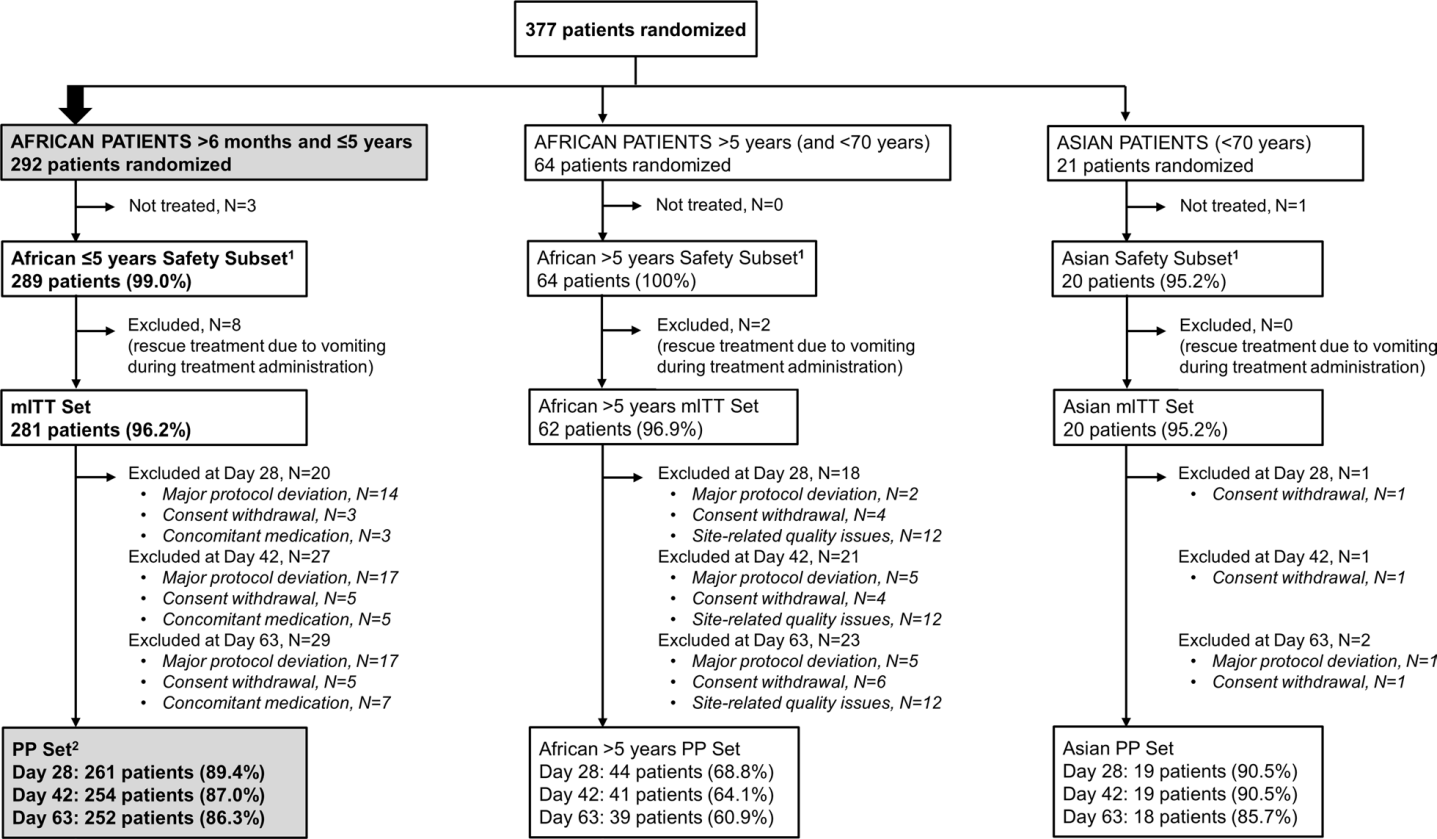
Study analysis sets. ^1^ Safety analyses were conducted in the overall Safety Set, including all randomized patients who received at least one dose or part of a dose of the study drug, regardless of age and region (n = 373). In addition, safety analyses were also performed in the 3 separate Safety Subsets including the African patients ≤ 5 years, the African patients > 5 years and the Asian patients, respectively. ^2^ The primary efficacy analysis was conducted in the PP Set at Day 28 (African patients ≤ 5 years only). Note: three African sites had quality issues related to the reading of the slides by microscopy. For one site, issues were detected before database lock and the patients were excluded from the PP Sets. All doses of artefenomel and ferroquine are expressed as adult-equivalent doses

A total of 261 African patients ≤ 5 years (89.4% of 292 randomized) were included in the PP Set at Day 28 (primary efficacy population, see Fig. 2). Three African sites (out of 12 study sites which randomized at least 1 patient) had data quality issues related to the reading of the slides by microscopy. For one site, issues were detected before database lock and the patients (n = 12, all African > 5 years) were excluded from the African > 5 years PP Set (Fig. 2). For the other two sites (n = 34), issues were detected after database lock.

#### Patient demographics and baseline characteristics

Overall, the majority of the 377 randomized patients (70.0%) were aged > 2 to ≤ 5 years and approximately half (48.0%) weighed between ≥ 10 to < 15 kg (see Additional file 2: Table S2). Most key baseline characteristics were similar across treatment arms. In the primary population of interest (African children ≤ 5 years), most of the 292 randomized patients (90.4%) were aged > 2 years to ≤ 5 years, and 9.6% of patients were > 6 months to ≤ 2 years (Table 2). A total of 62.0% of patients weighed between ≥ 10 to < 15 kg, but no patient was recruited in the ≥ 5 to < 7 kg body weight group. Key baseline characteristics in African patients ≤ 5 years were similar across treatment arms, except for a lower proportion of male patients and a lower median baseline parasitaemia in the artefenomel + high-dose ferroquine arm compared to the other arms.

**Table 2:** Demographics and patient characteristics by region (randomized patients)

	Artefenomel mg: Ferroquine mg				
	800:400	800:600	800:900	800:1200	Total
African patients > 6 months and ≤ 5 years					
N	73	74	73	72	292
Males, n (%)	43 (58.9)	35 (47.3)	41 (56.2)	27 (37.5)	146 (50.0)
Age (months), median (range)	40.74 (9.0; 59.6)	41.10 (8.7; 59.9)	40.05 (10.3; 59.9)	40.89 (7.6; 59.2)	40.30 (7.6; 59.9)
> 6 months to ≤ 2 years, n (%)^a^	7 (9.6)	8 (10.8)	6 (8.2)	7 (9.7)	28 (9.6)
> 2 years to ≤ 5 years, n (%)	66 (90.4)	66 (89.2)	67 (91.8)	65 (90.3)	264 (90.4)
Body weight (kg), median (range)	13.10 (8.2; 20.0)	13.85 (8.0; 19.0)	13.10 (7.7; 19.0)	13.75 (8.1; 21.5)	13.50 (7.7; 21.5)
Baseline parasitaemia (/µL), median (range)^b^	34,400 (18; 182,719)	38,153.5 (1060; 177,867)	31,164.5 (599; 145,053)	22,793 (920; 119,804)	31,219 (18; 182,719)
African patients > 5 years					
N	16	15	18	15	64
Males, n (%)	9 (56.3)	9 (60.0)	8 (44.4)	5 (33.3)	31 (48.4)
Age (years), median (range)	14.58 (6.2; 56.0)	15.90 (7.2; 32.9)	15.71 (6.1; 43.7)	16.28 (5.2; 48.3)	15.24 (5.2; 56.0)
> 5 years to ≤ 14 years, n (%)	6 (37.5)	5 (33.3)	6 (33.3)	5 (33.3)	22 (34.4)
> 14 years to ≤ 18 years, n (%)	5 (31.3)	5 (33.3)	5 (27.8)	4 (26.7)	19 (29.7)
> 18 years	5 (31.3)	5 (33.3)	7 (38.9)	6 (40.0)	23 (35.9)
Body weight (kg), median (range)	47.20 (18.8; 70.2)	47.60 (19.9; 77.3)	44.85 (19.3; 89.0)	50.70 (15.0; 75.0)	47.00 (15.0; 89.0)
Baseline parasitaemia (/µL), median (range)^b^	4693.0 (885; 72,317)	21,968.0 (550; 162,303)	4280.0 (684; 85,130)	5321.0 (1060; 103,947)	5962.0 (550; 162,303)
Asian patients					
N	4	5	6	6	21
Males, n (%)	4 (100)	4 (80.0)	5 (83.3)	5 (83.3)	18 (85.7)
Age (years), median (range)	28.39 (15.4; 32.9)	24.96 (19.8; 61.9)	28.12 (14.7; 55.8)	28.91 (21.2; 53.4)	27.38 (14.7; 61.9)
> 6 months to ≤ 2 years, n (%)	0	0	0	0	0
> 2 years to ≤ 5 years, n (%)	0	0	0	0	0
> 5 years to ≤ 14 years, n (%)	0	0	0	0	0
> 14 years to ≤ 18 years, n (%)	1 (25.0)	0	2 (33.3)	0	3 (14.3)
> 18 years	3 (75.0)	5 (100)	4 (66.7)	6 (100)	18 (85.7)
Body weight (kg), median (range)	52.50 (46.0; 55.0)	55.00 (47.0; 63.0)	55.50 (44.0; 62.0)	55.50 (50.0; 63.0)	55.00 (44.0; 63.0)
B aseline parasitaemia (/µL), median (range)^b^	19,681.5 (1280; 44,951)	2698.0 (1562; 26,190)	24,274.5 (7512; 95,251)	15,702.0 (1592; 63,985)	19,627.0 (1280; 95,251)

N: total number of patients in the relevant analysis set. All doses of artefenomel and ferroquine are expressed as adult-equivalent doses

^a^Including 11 patients (3.8%) aged < 12 months (2 with the artefenomel + 400 mg ferroquine dose, 2 with the 600 mg dose, 4 with the 900 mg dose and 3 with the 1200 mg dose)

^b^Baseline parasitaemia corresponds to the baseline/post-dose measurement and was summarized for all treated patients (i.e. Safety Set, including 289 African patients ≤ 5 years, 64 African patients > 5 years and 20 Asian patients). Baseline parasitaemia is missing for 3 African patients > 5 years

Asian patients were from two sites in different parts of Vietnam, and most were males (85.7%) and aged ≥ 18 years (85.7%). All Asian patients were > 14 years of age (Table 2).

#### Compliance/exposure

Of the 373 patients who received study treatment (Safety Set), 7 patients (1.9%) vomited during or after ferroquine administration, with no clear trend between the ferroquine dose and vomiting rate (see Additional file 2: Table S3). These patients received rescue treatment and were discontinued from the study.

Of the 366 patients successfully dosed with ferroquine and who received artefenomel, 90 patients (24.6%) vomited within 6 h of initial artefenomel administration, with no clear trend between the ferroquine dose and vomiting rate (range for the 4 treatment arms: 23.3% to 25.8%). Of the 90 patients who vomited, 6 vomited within 5 min of initial artefenomel administration and were re-dosed (2 of whom vomited again after re-administration).

The overall vomiting rate for the combined treatment was 26.0% (97 patients vomited out of the 373 patients who received at least one dose or part of a dose of the study drug).

#### PCR-adjusted ACPR in African patients ≤ 5 years (PP Set)

**Fig. 3:**
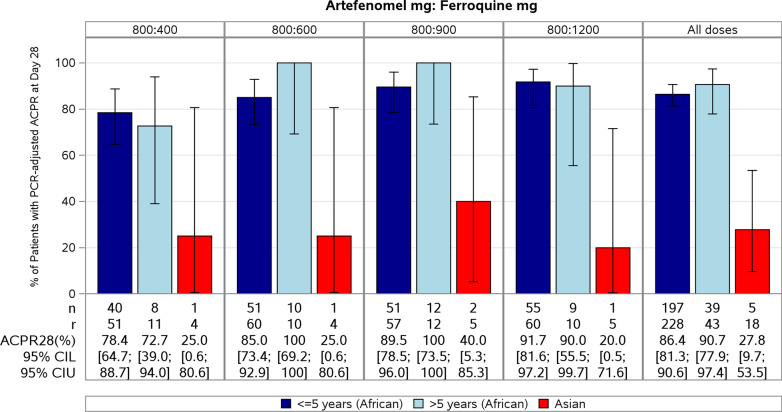
PCR-adjusted ACPR at Day 28 by age (African patients) and by region (PP Sets). All doses of artefenomel and ferroquine are expressed as adult-equivalent doses. n: number of patients in each category achieving ACPR. r: total number of patients in the relevant analysis set with a defined response of Cure or Failure (i.e. patients evaluable for the outcome considered). 95% CI L: lower limit of 95% confidence interval; 95% CI U: upper limit of 95% confidence interval

The primary efficacy endpoint, PCR-adjusted ACPR at Day 28 in the primary population of interest, African patients ≤ 5 years (PP Set), is presented by treatment arm in Table 3 and depicted in Fig. 3. Note that all doses are expressed as adult-equivalent doses. Actual doses administered across all weight bands are given in Additional file 1: Table S1.

**Table 3:** Crude and PCR-adjusted ACPR at each time point in African patients aged ≤ 5 years (PP Set)

	Artefenomel mg: Ferroquine mg				
	800:400	800:600	800:900	800:1200	Total
Day 28					
N	69	67	63	62	261
Crude ACPR, n/r (%)	34/69 (49.3)	48/67 (71.6)	48/63 (76.2)	54/62 (87.1)	184/261 (70.5)
[95% CI]	[37.0; 61.6]	[59.3; 82.0]	[63.8; 86.0]	[76.1; 94.3]	[64.6; 76.0]
PCR-adjusted ACPR, n/r (%)	40/51 (78.4)	51/60 (85.0)	51/57 (89.5)	55/60 (91.7)	197/228 (86.4)
[95% CI]	[64.7; 88.7]	[73.4; 92.9]	[78.5; 96.0]	[81.6; 97.2]	[81.3; 90.6]
Day 42					
N	69	65	59	61	254
Crude ACPR, n/r (%)	27/69 (39.1)	33/65 (50.8)	36/59 (61.0)	42/61 (68.9)	138/254 (54.3)
[95% CI]	[27.6; 51.6]	[38.1; 63.4]	[47.4; 73.5]	[55.7; 80.1]	[48.0; 60.6]
PCR-adjusted ACPR, n/r (%)	30/43 (69.8)	45/55 (81.8)	42/50 (84.0)	50/57 (87.7)	167/205 (81.5)
[95% CI]	[53.9; 82.8]	[69.1; 90.9]	[70.9; 92.8]	[76.3; 94.9]	[75.5; 86.5]
Day 63					
N	68	65	59	60	252
Crude ACPR, n/r (%)	24/68 (35.3)	30/65 (46.2)	34/59 (57.6)	35/60 (58.3)	123/252 (48.8)
[95% CI]	[24.1; 47.8]	[33.7; 59.0]	[44.1; 70.4]	[44.9; 70.9]	[42.5; 55.2]
PCR-adjusted ACPR, n/r (%)	25/39 (64.1)	33/43 (76.7)	36/44 (81.8)	41/47 (87.2)	135/173 (78.0)
[95% CI]	[47.2; 78.8]	[61.4; 88.2]	[67.3; 91.8]	[74.3; 95.2]	[71.1; 84.0]

N: total number of patients in the relevant analysis set. All doses of artefenomel and ferroquine are expressed as adult-equivalent doses

n: number of patients in each category achieving ACPR

r: total number of patients in the relevant analysis set with a defined response of Cure or Failure (i.e. patients evaluable for the outcome considered)

All treatment arms failed the protocol-defined efficacy criterion for PCR-adjusted ACPR at Day 28 (i.e. treatment arms failed to achieve the lower limit of 95% CI > 90%). A trend was apparent between ferroquine dose and the PCR-adjusted ACPR at Day 28 for the PP Set, with efficacy rates ranging from 78.4% (95% CI 64.7 to 88.7%) with the artefenomel + 400 mg ferroquine dose up to 91.7% (95% CI 81.6 to 97.2%) with the 1200 mg ferroquine dose. Only 1 patient overall experienced an ETF, in the treatment arm randomized to artefenomel + 600 mg ferroquine dose.

The exclusion of two sites with data quality issues related to microscopy did not substantially affect the primary results at Day 28 (*post-hoc* analysis). Efficacy rates ranged from 77.8% (95% CI 62.9 to 88.8%) with the artefenomel + 400 mg ferroquine dose to 92.3% (95% CI 81.5 to 97.9%) with the artefenomel + 1200 mg ferroquine dose.

**Fig. 4:**
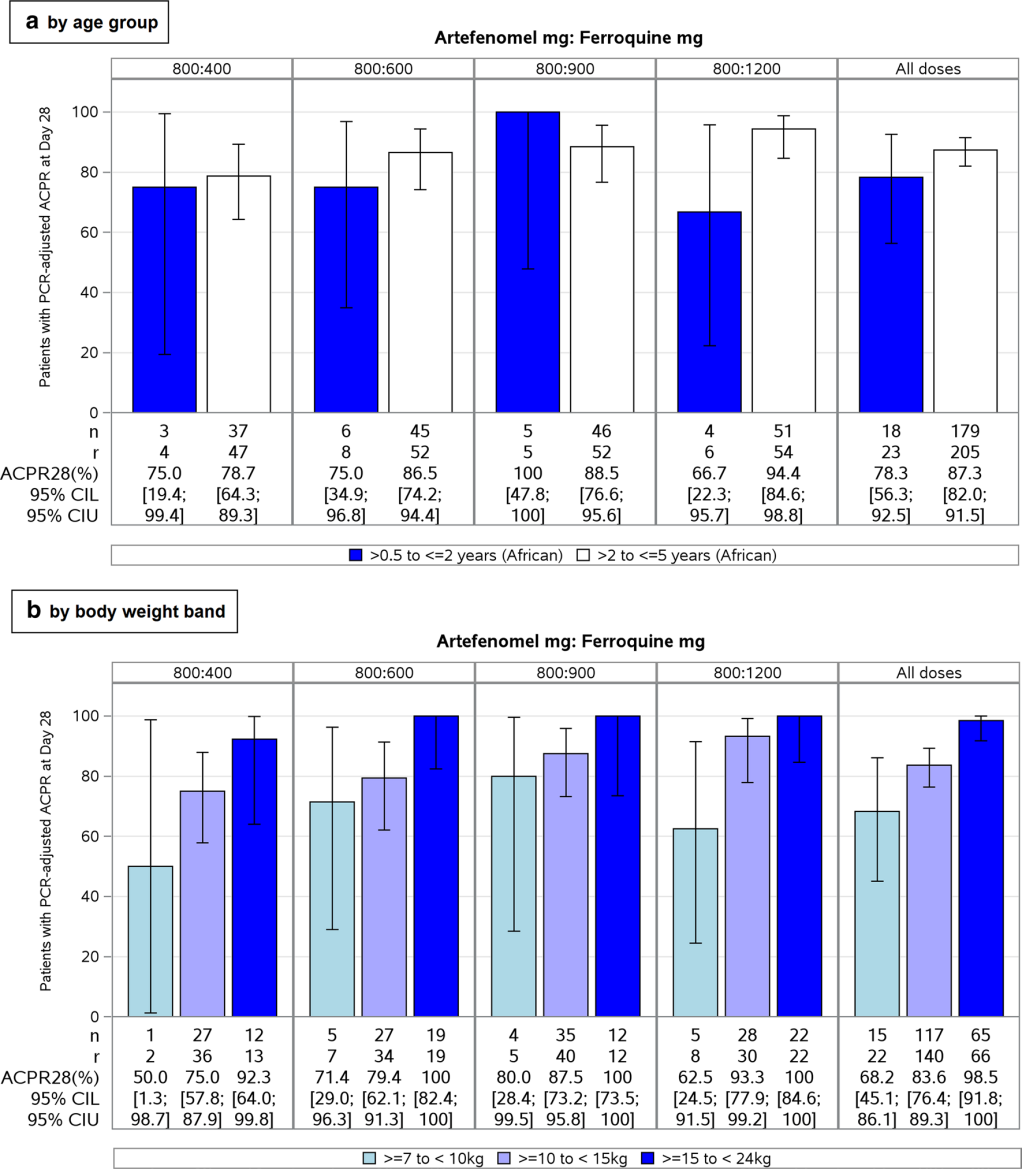
PCR-adjusted ACPR at Day 28 in African patients ≤ 5 years by **a** age group and **b** body weight band (PP Set). All doses of artefenomel and ferroquine are expressed as adult-equivalent doses. n: number of patients in each category achieving ACPR. r: total number of patients in the relevant analysis set with a defined response of Cure or Failure (i.e. patients evaluable for the outcome considered). 95% CI L: lower limit of 95% confidence interval; 95% CI U: upper limit of 95% confidence interval

PCR-adjusted ACPR appeared to decrease over time, as expected (Table 3), although this reduction was less apparent with higher ferroquine doses. PCR-adjusted ACPR was 87.2% at Day 63 with the highest ferroquine dose (1200 mg) in the PP Set. There was a trend towards lower efficacy with lower weight band and younger age group (Fig. 4), although the number of children was very small for the lowest weight band and youngest age group. There was no clear trend in PCR-adjusted ACPR at Day 28 according to baseline parasitaemia quartile (Additional file 2: Table S4).

#### Crude ACPR in African patients ≤ 5 years (PP set)

Crude ACPR at Day 28 ranged from 49.3% with the 400 mg ferroquine dose to 87.1% with the 1200 mg ferroquine dose in the PP Set (Table 3). Similar trends with respect to age (when considering all ferroquine doses) and weight band were observed as for PCR-adjusted ACPR at Day 28 (Additional file 2: Table S5).

#### PCR-adjusted and crude ACPR in African patients ≤ 5 years (mITT Set)

For the mITT Set, a similar trend between ferroquine dose and efficacy was apparent. Crude ACPR at Day 28 ranged from 49.3% with the artefenomel + 400 mg ferroquine dose to 81.2% with the 1200 mg ferroquine dose (Additional file 2: Table S6).

**Fig. 5:**
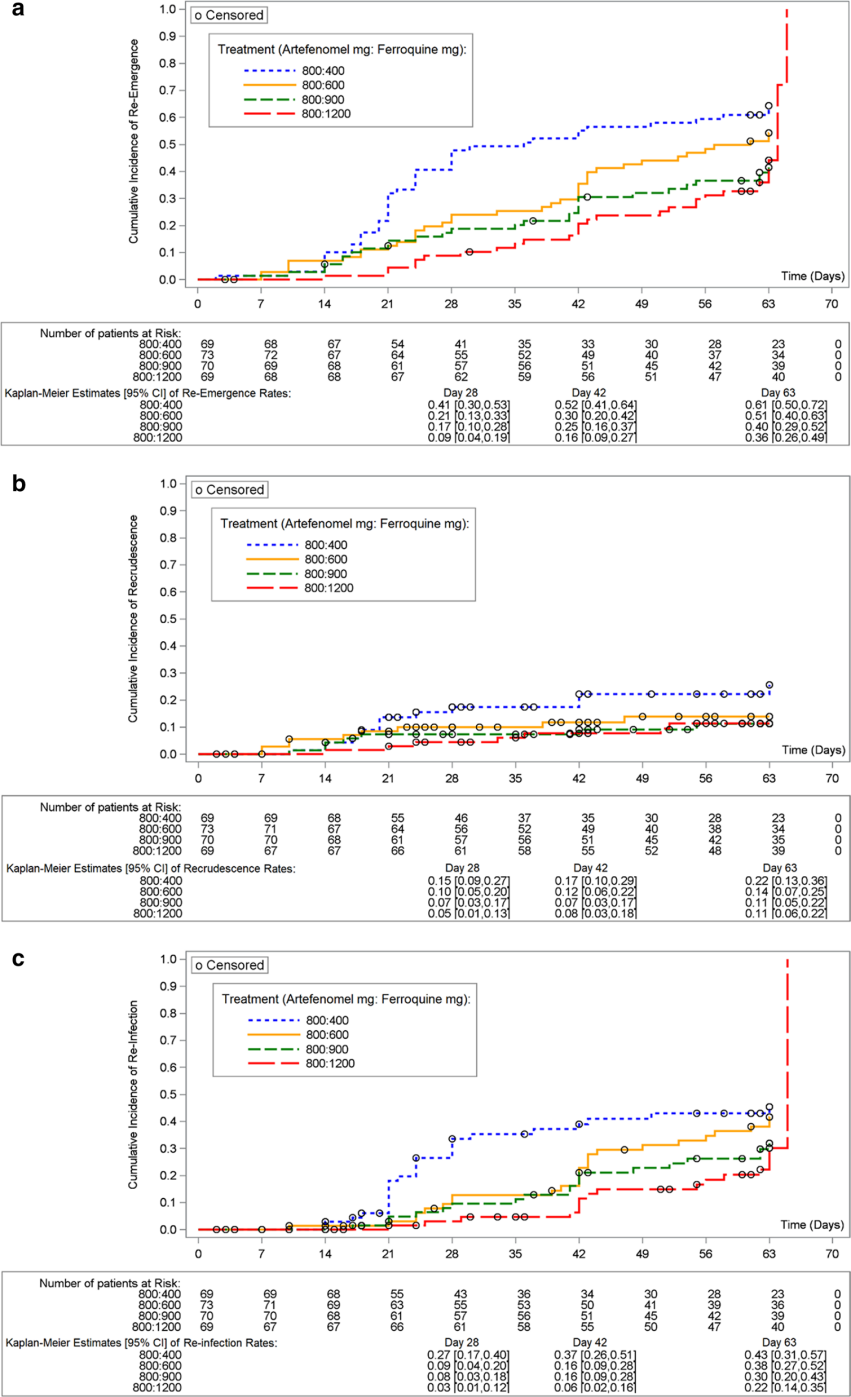
Kaplan–Meier cumulative incidence curve for time to **a** re-emergence, **b** recrudescence and **c** re-infection in African patients aged ≤ 5 years (mITT Set). Kaplan–Meier estimates below each graph are estimates of the cumulative incidence rates. All doses of artefenomel and ferroquine are expressed as adult-equivalent doses. Patients with no event were censored at the time of study completion, premature study discontinuation, including switch to established anti-malarial treatment or start of any other treatment with anti-malarial activity, whichever was earliest

Incidence rates of re-emergence, recrudescence and re-infection increased over time (Fig. 5), with a clear inverse relationship with dose observed at each time point, as expected. Median time to re-emergence ranged from 36.0 days with the 400 mg ferroquine dose to 64.0 days with the 1200 mg ferroquine dose (Additional file 2: Table S7).

#### ACPR in Asian patients

The number of Asian patients was small (19 in the Asian PP Set at Day 28). Efficacy rates were low across all ferroquine doses at all time points, with PCR-adjusted and crude ACPR ranging from 20 to 40% at Day 28 (Fig. 3 and Additional file 2: Table S8). Treatment failure was mainly due to recrudescence, with 12 of the 13 events of re-emergence observed in the Asian mITT Set.

being due to recrudescence (Additional file 2: Table S9).

#### ACPR in African patients > 5 years

The number of African patients > 5 years was small (44 in the African > 5 years PP Set at Day 28, with 10 to 12 patients per treatment arm). PCR-adjusted ACPR at Day 28 ranged from 72.7% to 100%, with no apparent trend with ferroquine dose (Fig. 3 and Additional file 2: Table S8). A trend between ferroquine dose and efficacy was observed in the African > 5 years mITT Set (see Additional file 2: Table S10).

#### Parasite clearance kinetics

Kaplan–Meier analysis of time to initial asexual parasite clearance (ie, PCT) is presented by region for the PP Sets in Table S11 (see Additional file 2). In African patients ≤ 5 years, there was no trend between ferroquine dose and PCT, or the percentage of patients achieving parasite clearance. Median PCT across all ferroquine doses was 36.0 h, with 76.7% of patients achieving parasite clearance after 72 h. Lower parasitaemia at baseline (lowest quartile, i.e. ≤ 9082 µL) was associated with an apparently faster parasite clearance with a median PCT of 24.0 h and higher percentage of patients achieving parasite clearance at 72 h (92.3%), compared to quartiles with baseline parasitaemia > 9082 µL (median PCT of 36.1 to 42.1 h, and 66.4% to 79.5% of patients achieving parasite clearance at 72 h, depending on the quartile). In African patients > 5 years, median PCT across all ferroquine doses was 24.2 h, with 90.9% of patients achieving parasite clearance at 72 h. In Asian patients, parasite clearance was slow relative to African patients: median PCT was 79.8 h across all ferroquine doses, with 25.0%, 40.0% and 40.0% of patients achieving parasite clearance at 72 h with the 600 mg, 900 mg and 1200 mg ferroquine dose, respectively. However, the number of patients in each arm was small (n = 4 to 5).

Parasite clearance parameters are provided in Table S12 (see Additional file 2). In African patients ≤ 5 years (PP Set), across all ferroquine doses, median PCt1/2 was 2.6 h (range: 1.3 to 6.6 h), median PRR48 (log10) was 5.6 and median time to 90% parasite reduction was 10.8 h. Similar values were observed in African patients > 5 years: median PCt1/2 was 2.2 h, median PRR48 (log10) was 6.6 and median time to 90% parasite reduction was 9.5 h. Across all ferroquine doses, parasite clearance was slower in Asian patients than in African patients. In the Asian PP Set, median PCt1/2 was 6.0 h (n = 19), median PRR48 (log10) was 2.4 and the median time to 90% parasite reduction was 20.0 h.

Results regarding fever clearance time (FCT) and time to gametocyte appearance/clearance are presented in Additional File 2.

#### Kelch-13 genotype

For *P. falciparum* infections, a total of 34 loci were tested for *kelch-13* mutation. Analyses were carried out in the African population across all age groups (i.e. ≤ 5 years and > 5 years). Results are presented for the mITT Sets (n = 294) and the PP Sets (n = 272). Infections in almost all African patients had no mutation at any locus and so were true wild type (WT): 284 patients in the mITT Sets (96.6%) and 262 patients in the PP Sets (96.3%). In total, 6 distinct *kelch-13* mutations were detected in 10 patients, 2 of which (A578S and S522C) were associated with artemisinin resistance, as previously reported by Ashley and colleagues [12].

For *P. falciparum* infections in the Asian population, almost all Asian patients carried the C580Y mutation only: 18/20 patients in the mITT Set (90.0%) and 17/19 patients in the PP Set (89.5%). Only 2 patients were true WT.

#### Parasite clearance and association with kelch-13 mutation (PP Sets)

**Fig. 6:**
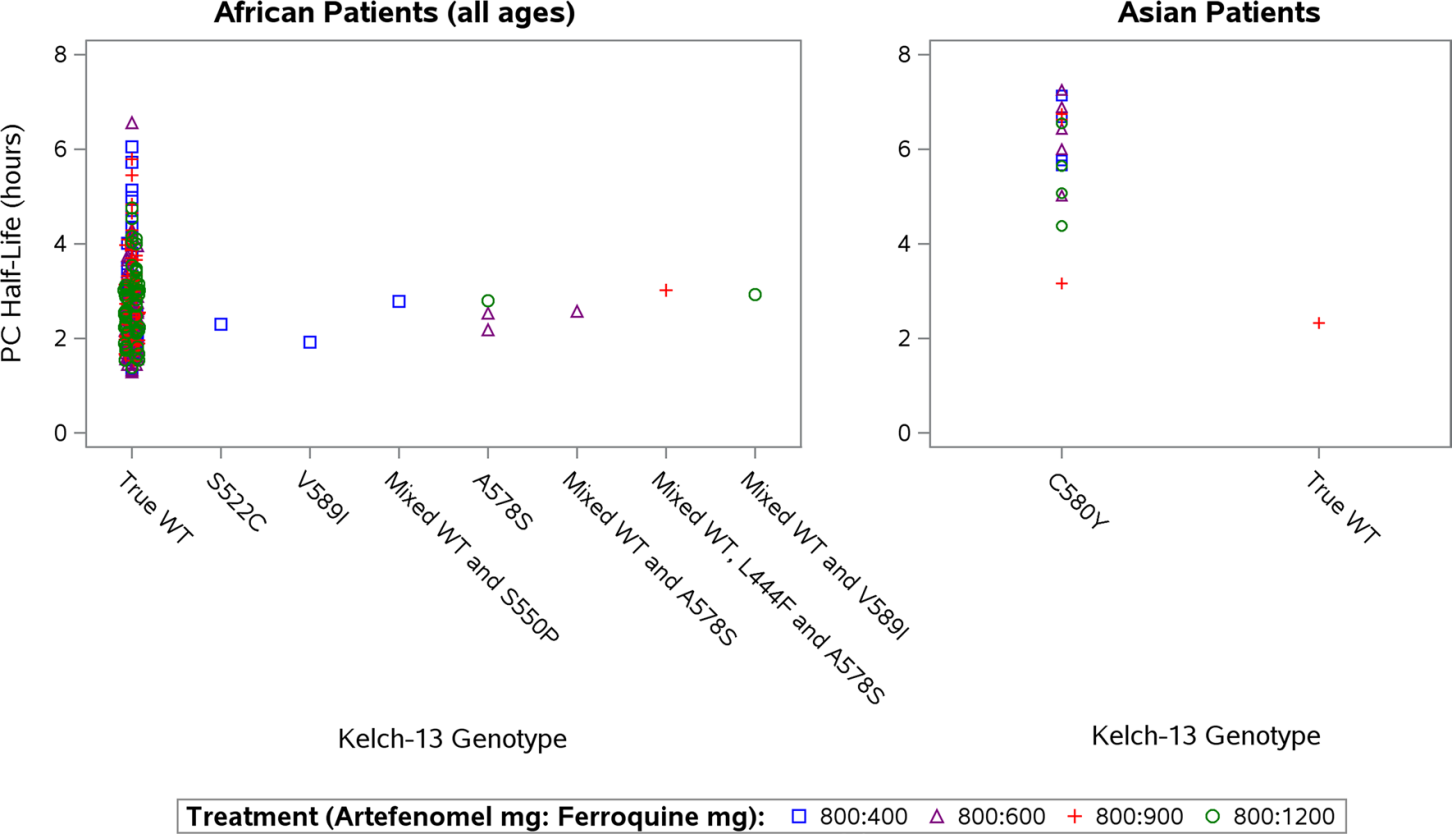
Relationship between parasite clearance half-life and *kelch-13* genotype by region (PP Sets). Only patients with both genotyping and PCt1/2 results were included (n = 240). The patient with the mixed infection including the E556G mutation was not included due to the lack of a PCt1/2 result. PC half-life: parasite clearance half-life; mixed WT: mixed wild type (presence of mutants and wild type); true WT: true wild type (no mutations at any of the tested loci)

The median PCt1/2 for the African patients across all ferroquine doses was 2.6 h (range 1.3 to 6.6 h, n = 239). Scatter plots of the correlation of PCt1/2 and *kelch-13* genotype in the PP Sets are provided in Fig. 6 (with the African and Asian patients shown separately). The influence of *kelch-13* genotype on PCt1/2 for African patients could not be evaluated because only a small fraction of the patients carried *kelch-13* mutations. Of the 2 Asian patients with WT genotype, 1 had an evaluable PCt1/2 (2.3 h), while the median PCt1/2 for the 17 Asian patients with C580Y mutation (16 evaluable) was 6.2 h, with a range from 3.2 to 7.2 h.

#### Pharmacokinetics

**Fig. 7:**
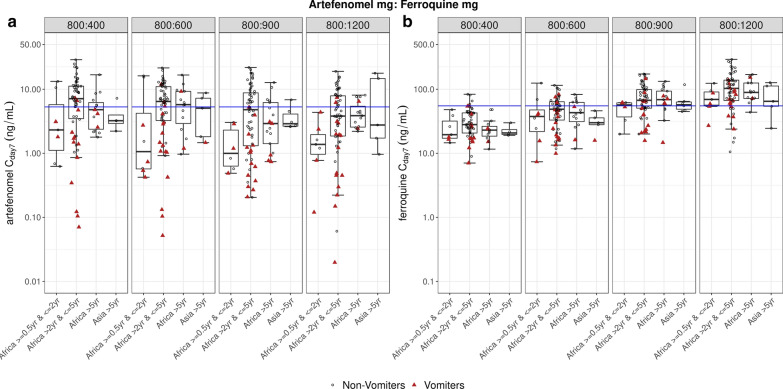
Estimated Day 7 concentrations of artefenomel (**a**) and ferroquine (**b**) by treatment arm and region/age group. All doses of artefenomel and ferroquine are expressed as adult-equivalent doses. The boxplots show the sample medians, with the first and third quartiles. The whiskers represent the lowest/largest values no further than 1.5 times the interquartile range. Circles and triangles are individual patient values. Blue lines represent the median C_day7_ in the exposure–response dataset: artefenomel 5.3 ng/mL and ferroquine 55 ng/mL. The PK Sets included all patients who received each study drug and had at least one evaluable blood sample for PK (n = 364 for artefenomel; n = 367 for ferroquine)

Individual artefenomel and ferroquine/SSR97213 exposures could be estimated for 364 and 367 patients respectively (including patients who vomited and were not successfully re-dosed). Summaries of the individual estimated exposures by region and age group are provided in *S3 Pharmacokinetic analysis details* (see Additional file 3). C_day7_ is summarized across region and age group in Fig. 7; C_max_ and AUC showed a similar pattern.

**Fig. 8:**
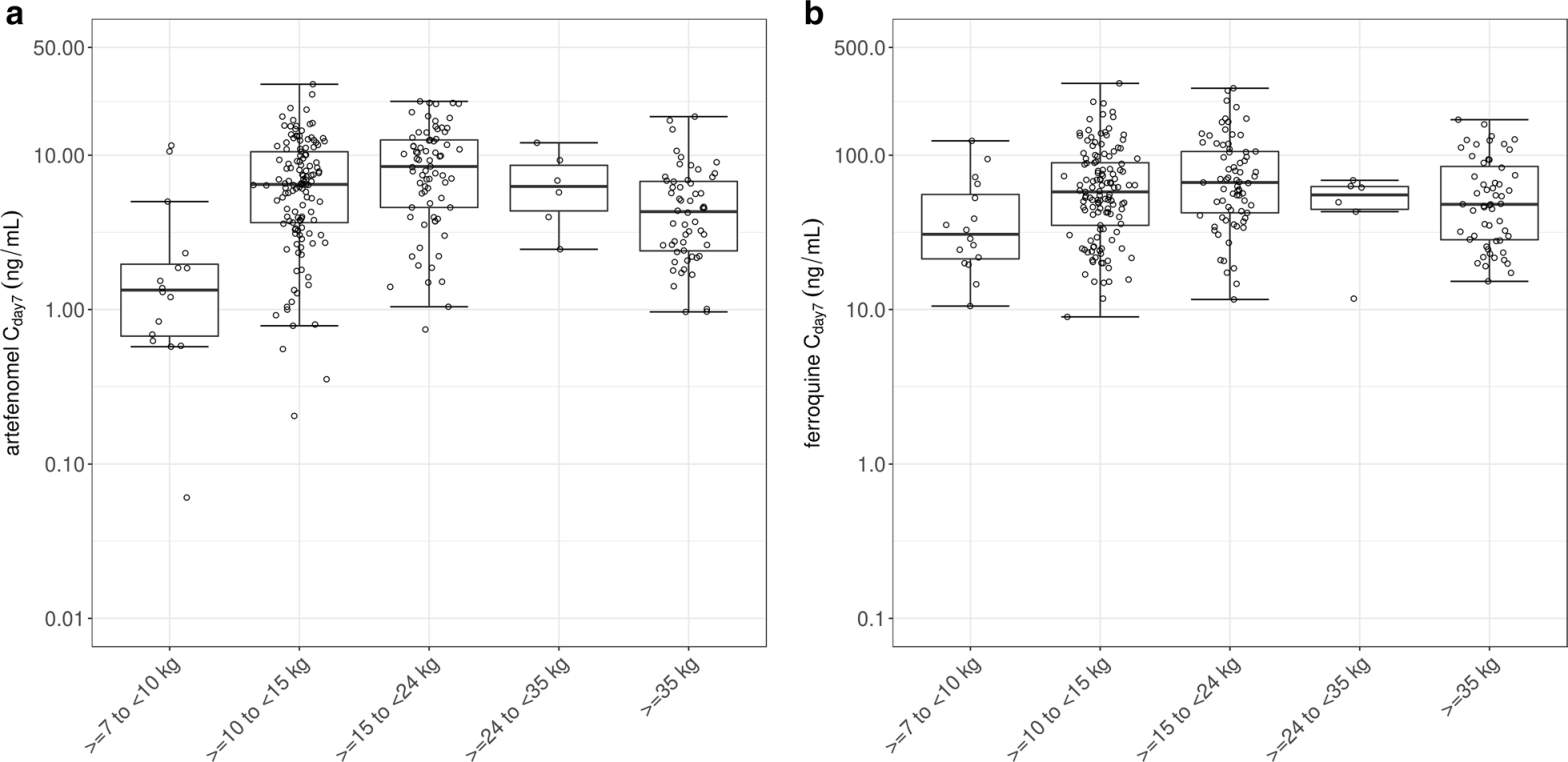
Estimated Day 7 concentrations of artefenomel (**a**) and ferroquine (**b**) across the treatment arms by body weight band (excluding vomiters). The boxplots show the sample medians, with the first and third quartiles. The whiskers represent the lowest/largest values no further than 1.5 times the interquartile range. Circles are individual patient values of non-vomiters (n = 280 for artefenomel and 276 for ferroquine)

Exposures to artefenomel showed substantial between-patient variability, with a coefficient of variation (CV) of 170 to 196% across treatment arms for C_day7_. Exposures were comparable across regions and age groups, except in the youngest African children (> 6 months to ≤ 2 years) and the lowest weight band (≥ 7 to < 10 kg) who had lower exposures (Figs. 7 and 8, respectively). However, it must be noted that there were ≤ 10 patients both in the youngest age group and the lightest weight group. The artefenomel exposures were comparable across the 4 treatment arms.

Exposures of ferroquine were comparable across regions and age groups and were approximately dose-proportional (Fig. 7), with a moderate-to-high variability between patients (CV 51 to 70% for C_day7_). Similarly, exposures of ferroquine active metabolite (SSR97213, see Additional file 3) were also comparable across regions and age groups.

Patients who vomited had lower exposures than non-vomiters (see Additional file 3). Exposures of artefenomel in vomiters were about 25% of that in non-vomiters. Exposures of ferroquine in vomiters were about 67–70% of the exposure in non-vomiters. The non-vomiters also showed considerably lower between-patient variability in exposures of artefenomel (CV of non-vomiters 92 to 135% for C_day7_, versus 112 to 312% for vomiters). The Asian patients had similar exposures of artefenomel and ferroquine/SSR97213 compared to the African patients > 5 years.

Comparing exposures across the body weight bands (for non-vomiters only and across treatment arms) suggests similar exposures across the bands, except for artefenomel which showed lower exposures in patients in the lowest body weight band (≥ 7 to < 10 kg, Fig. 8).

#### Efficacy exposure–response analysis

**Fig. 9:**
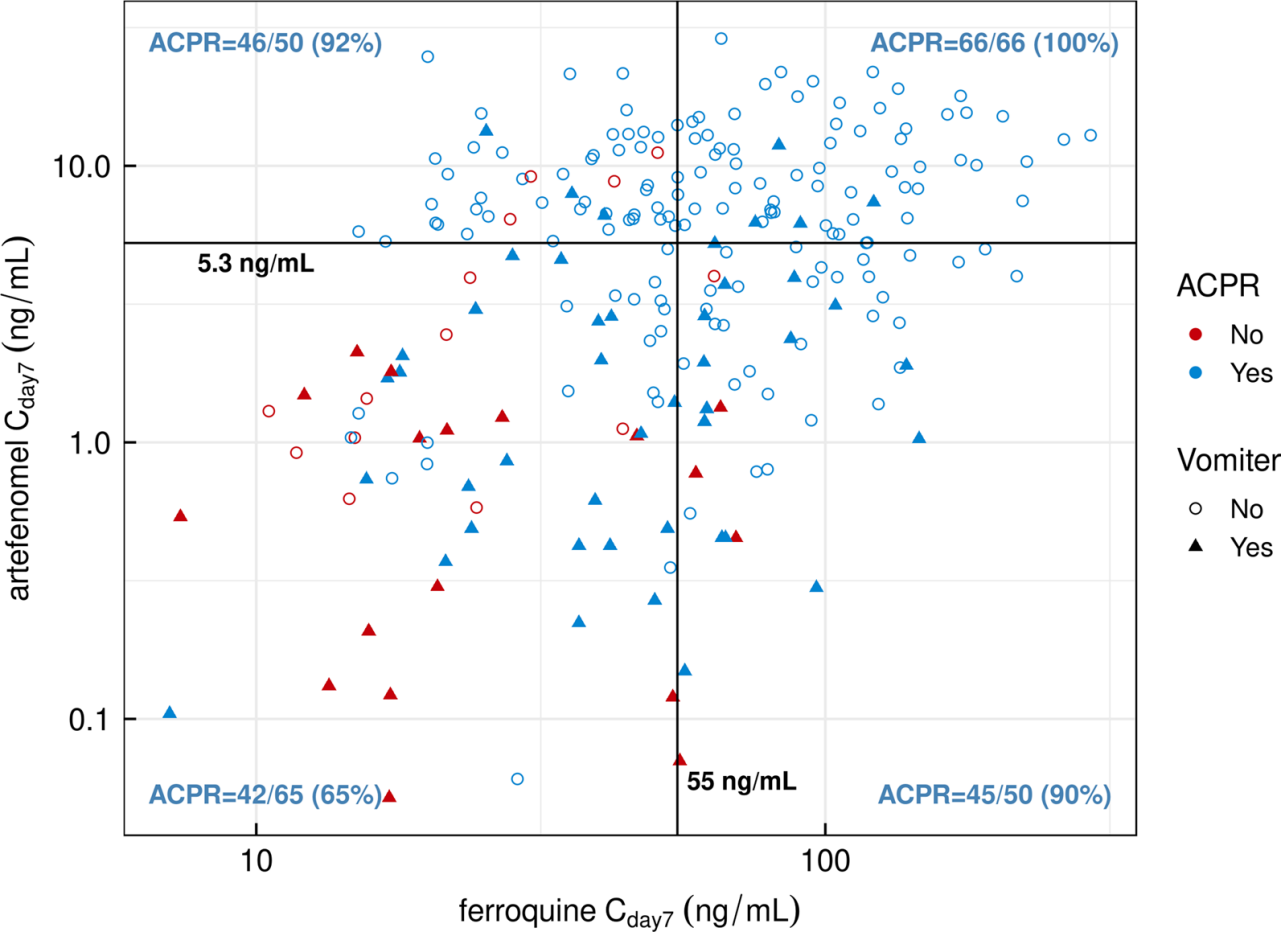
Estimated Day 7 concentrations of artefenomel and ferroquine in individual patients grouped by vomiting status and PCR-adjusted ACPR at Day 28 (African patients ≤ 5 years). All African patients ≤ 5 years with PCR-adjusted ACPR at Day 28 and exposure to both drugs were included (n = 231). The scatter plot displays the relationship between exposures to ferroquine (X axis) and artefenomel (Y axis) in African patients ≤ 5 years. Four quadrants were then defined according to the median artefenomel and ferroquine exposures, and the PCR-adjusted ACPR at Day 28 is provided for the patients belonging to each quadrant

As an exploratory objective, the relationship between artefenomel and ferroquine exposure, as well as other covariates, and ACPR at Day 28 was analysed with a logistic regression model. Details are provided in *S4 Exposure–response analysis details* (see Additional file 4). The analysis data set comprised 298 patients. The data suggested a clear relationship between the C_day7_ of artefenomel as well as ferroquine and the clinical outcome (PCR-adjusted ACPR at Day 28), as shown for African patients ≤ 5 years in Fig. 9 (n = 231). All 66 patients who had C_day7_ of both compounds above their observed median value (depicted as horizontal and vertical lines on the plot) achieved PCR-adjusted ACPR at Day 28 (efficacy rate = 100%). In contrast, for the 65 patients who had the C_day7_ of both compounds below their median value, the PCR-adjusted ACPR at Day 28 was only 65%. If one of the compounds only was above its median value, the PCR-adjusted ACPR at Day 28 was about 90%. These observations clearly indicate that both compounds contributed to the ACPR at Day 28.

**Fig. 10:**
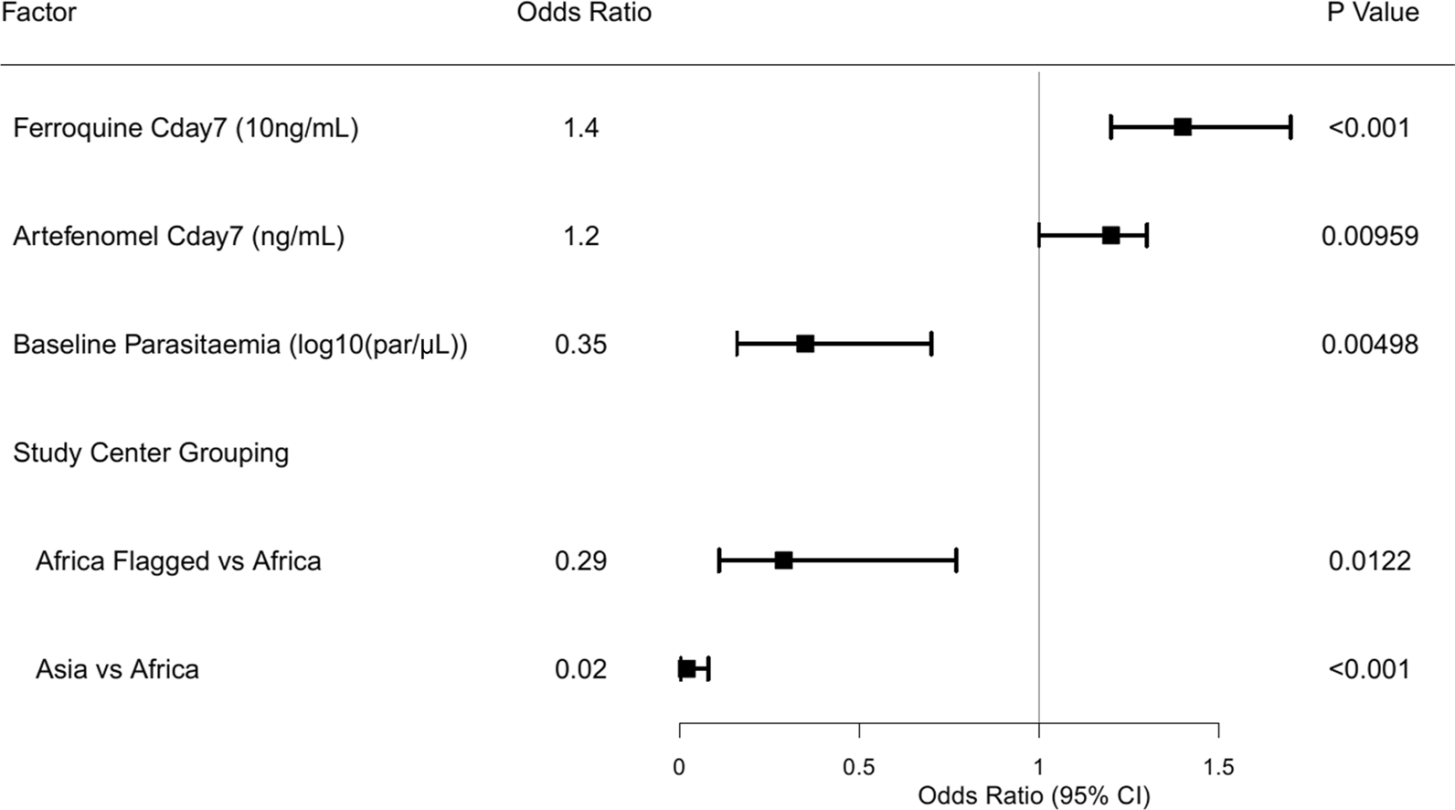
Logistic regression result for PCR-adjusted ACPR at Day 28. All patients with PCR-adjusted ACPR at Day 28 and exposure to both drugs were included in the analysis (n = 298). Note: 'Africa Flagged' refers to the three African sites where quality issues related to the reading of the slides by microscopy were detected

The logistic regression analysis indicated that the PCR-adjusted ACPR at Day 28 could be described as a function of artefenomel (p = 0.01) and ferroquine (p < 0.001) C_day7_, baseline parasitaemia (p < 0.005), and a study site grouping (African sites with data quality issues related to microscopy [p = 0.012], or Asian sites [p < 0.001] versus other African sites) (Fig. 10 and Additional file 4). The odds of achieving ACPR at Day 28 increased 1.4-fold per 10 ng/mL increase in ferroquine C_day7_ and increased 1.2-fold per 1 ng/mL increase in artefenomel C_day7_, respectively. In contrast, the odds of achieving ACPR at Day 28 decreased 2.9-fold for each log increase in baseline parasitaemia (parasites/μL). The odds of achieving ACPR at Day 28 in Asian patients were only 0.02 of that in African patients, although few Asian patients (n = 18) were in the dataset, and these were all from Vietnam. Reduced odds were also identified for the three African study sites that were flagged for data quality issues related to microscopy. No effect of age, sex or SSR97213 C_day7_ could be identified after taking artefenomel and ferroquine exposure as well as baseline parasitaemia into account in the model. The impact of *kelch-13* genotype at screening could not be evaluated because of its correlation with region (see Additional file 4). SSR97213 exposure was not identified as contributing to the efficacy, despite having similar potency and exposures as ferroquine. This is most likely because of the high correlation between parent and metabolite exposures. Ferroquine exposures should therefore be considered as a proxy for the combined exposure of both.

**Fig. 11:**
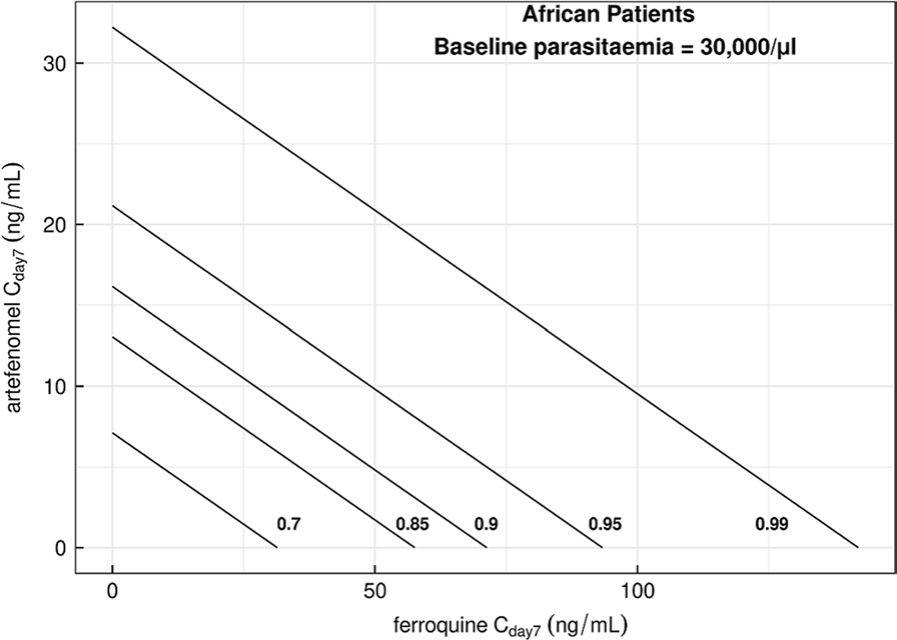
Isobolograms for various levels of PCR-adjusted ACPR at Day 28 in African patients, regardless of age (baseline parasitaemia = 30,000/μL). The figure shows the isobolograms for African patients for various probabilities of achieving ACPR at Day 28 with a baseline parasitaemia of 30,000 parasites/μL, which was the median baseline parasitaemia observed in African children ≤ 5 years

The combined exposures of ferroquine and artefenomel required to achieve various PCR-adjusted ACPR at Day 28 outcomes can be illustrated using isobolograms. Figure 11 shows the isobolograms for various probabilities of achieving ACPR at Day 28 for African patients with a baseline parasitaemia of 30,000 parasites /μL, i.e. the median baseline parasitaemia observed in African children ≤ 5 years. For patients with a baseline parasitaemia of 30,000 parasites /μL or less, a dosing regimen providing a combination of C_day7_ of artefenomel and ferroquine that is to the right of the ACPR at Day 28 = 0.95 isobole for all patients is projected to result in an ACPR at Day 28 > 0.95.

#### Safety and tolerability

Overall, 90.3% of patients had at least 1 TEAE and the frequencies were similar across treatment arms (see Additional file 2: Table S13). Permanent treatment discontinuation of either artefenomel or ferroquine was reported in 10 patients (2.7%), all due to TEAEs of vomiting: 7 vomited during or after administration of ferroquine but before administration of artefenomel, and 3 vomited during or after administration of artefenomel. Table 4 presents the incidence of TEAEs reported in ≥ 5% of patients in any treatment arm for the Safety Set (n = 373) up to Day 63. The most frequently reported TEAEs were malaria (corresponding to recrudescence or reinfection, 50.1%) and vomiting (32.7%). Malaria was more frequently reported with the lowest ferroquine doses (400/600 mg), while vomiting was more frequently reported with the highest ferroquine doses (900/1200 mg). Similar results were observed in African patients ≤ 5 years, who represent the majority of the Safety Set (289/373) (see Additional file 2: Table S14).

**Table 4:** Summary of treatment-emergent adverse events reported in ≥ 5% of patients in any treatment arm (Safety Set)

Preferred term	Artefenomel mg: Ferroquine mg				
	800:400	800:600	800:900	800:1200	Total
N	92	94	96	91	373
At least 1 TEAE	84 (91.3)	87 (92.6)	85 (88.5)	81 (89.0)	337 (90.3)
Malaria	55 (59.8)	50 (53.2)	41 (42.7)	41 (45.1)	187 (50.1)
Vomiting	27 (29.3)	27 (28.7)	34 (35.4)	34 (37.4)	122 (32.7)
Cough	10 (10.9)	7 (7.4)	15 (15.6)	15 (16.5)	47 (12.6)
Upper Respiratory Tract Infection	7 (7.6)	6 (6.4)	17 (17.7)	10 (11.0)	40 (10.7)
Diarrhoea	10 (10.9)	11 (11.7)	7 (7.3)	11 (12.1)	39 (10.5)
Pyrexia	7 (7.6)	2 (2.1)	9 (9.4)	10 (11.0)	28 (7.5)
Electrocardiogram QT Prolonged^a^	6 (6.5)	5 (5.3)	6 (6.3)	8 (8.8)	25 (6.7)
Headache	6 (6.5)	7 (7.4)	7 (7.3)	5 (5.5)	25 (6.7)
Abdominal pain	7 (7.6)	5 (5.3)	5 (5.2)	6 (6.6)	23 (6.2)
Decreased Appetite	5 (5.4)	8 (8.5)	5 (5.2)	5 (5.5)	23 (6.2)
Bronchitis	3 (3.3)	5 (5.3)	4 (4.2)	5 (5.5)	17 (4.6)
Rhinitis	4 (4.3)	3 (3.2)	1 (1.0)	7 (7.7)	15 (4.0)

MedDRA version 22.1. All doses of artefenomel and ferroquine are expressed as adult-equivalent doses

Data presented are the number (%) of patients with at least 1 TEAE in the Safety Set

TEAEs were AEs that developed or worsened or became serious during the on-treatment phase, i.e. time from the start of the first dose of study drug administration (included) up to the Day 63 visit (included)

Table sorted by decreasing frequency of preferred term in pooled treatment arms

^a^QTc prolongation was to be recorded as an AE (preferred term 'electrocardiogram QT prolonged') if it met any of the following criteria (i) symptomatic, (ii) requiring either corrective treatment or consultation, (iii) leading to treatment discontinuation or modification of dosing, (iv) fulfilling a seriousness criterion, (v) met the criteria for an AESI (QTcF ≥ 500 ms or QTcF prolongation > 60 ms from baseline)

The changes in haematological parameters were aligned with malaria and disease recovery.

No patients died during the study. Eight patients (2.1%), all Africans ≤ 5 years, experienced treatment-emergent SAEs: 2 in the artefenomel + ferroquine 600 mg arm (malaria and pharyngitis), 4 in the artefenomel + ferroquine 900 mg arm (malaria, drug-induced liver injury and 2 cases of ALT increase), and 2 in the artefenomel + ferroquine 1200 mg arm (hepatitis A, pneumonia aspiration).

With regards to hepatic safety, 8 patients (6 of whom were Africans ≤ 5 years) reported an AE related to liver that was considered as AESI, and in 4 of these patients, the AESI was also reported as an SAE (as 'other medically important condition'). The 8 patients with AESIs related to liver included 6 patients with ALT increase (reported at Day 15 for 3 patients and at Days 6, 8 and 29 for the other 3 patients; 2 AESIs were also reported as SAEs related either to artefenomel and ferroquine or ferroquine alone), 1 patient with drug-induced liver injury, who had an ALT increase > 20 × ULN (the AESI started at Day 29 and resolved by Day 43, and was also an SAE considered related to ferroquine only) and 1 patient diagnosed with hepatitis A (also reported as an SAE at Day 29). One of the 6 patients with ALT increase (108 U/L) also presented with an increase in bilirubin (51 µmol/L, 80% conjugated) at Day 8 (resolved at Day 11) consistent with a case of possible Hy's law but with no evidence of drug-induced liver injury (based on the DMC review of the case). All 8 hepatic AESIs were confirmed as resolved within 2 months after treatment administration (except one case of ALT increase which was confirmed as resolved later due to missed laboratory evaluations).

With regards to cardiac safety, heart rate (mean and median) decreased from baseline to post-baseline time points, with no evidence of a dose effect. Similarly, the lowest on-treatment heart rate values were similar across treatment arms. The QT interval corrected using the Bazett’s formula (QTcB) and using Fridericia's formula (QTcF) both increased on the day of study drug administration, in all treatment arms, peaking 4–6 h after administration. Mean and median values decreased thereafter. The increases in QTcF and QTcB tended to be greater in the higher ferroquine dose arms (900/1200 mg). There was evidence of a dose effect in terms of the incidence of QTcF and QTcB increases from baseline of > 60 ms, with the highest incidences reported in the highest ferroquine dose arm (1200 mg): 15.4% and 7.0%, respectively. No QT or QTcF prolongation > 500 ms was reported, while 5 patients (1.3%) (all African patients ≤ 5 years) had a QTcB prolongation > 500 ms, all of which were recorded 2–6 h after administration. Following the review of these 5 patients by an independent cardiologist, only 3 of the 5 patients of QTcB prolongation > 500 ms were confirmed. In 1 patient, the QTcB prolongation > 500 ms was associated with an increase from baseline > 60 ms (in artefenomel + ferroquine 900 mg arm), whereas in the 2 other patients, the QTcB increase from baseline was > 30 ms but < 60 ms (in artefenomel + ferroquine 400 mg and 900 mg arms). The ECG exposure–response analysis showed small increases in PR interval and QRS duration which were considered not clinically relevant, and an increase in QTcB exceeding 10 ms for ferroquine doses of 600 mg and higher, for which the clinical relevance remains to be established (see Additional file 5 for further details).

## Discussion

For the primary efficacy evaluation in the primary population of interest (African children aged > 6 months to ≤ 5 years, PP Set), artefenomel 800 mg in combination with ferroquine doses of 400, 600, 900 and 1200 mg (adult-equivalent doses) failed to achieve the protocol-defined efficacy criterion for PCR-adjusted ACPR at Day 28 (i.e. treatment arms failed to achieve the lower limit of 95% CI > 90%). For African children ≤ 5 years of age, a trend was apparent between ferroquine dose and PCR-adjusted ACPR at Day 28, and within this population, the highest dose group (1200 mg) showed a 91.7% response. There was a trend towards lower efficacy with lower weight band and younger age group, although the number of children weighing < 10 kg or aged ≤ 2 years was small. The overall variability in exposure was substantial for artefenomel (CV 170 to 196% across treatment arms for C_day7_) and moderate-to-high for ferroquine (CV 51 to 70% for C_day7_). Drug exposures were comparable across age and weight groups, except in the youngest and the lowest weight groups who had lower exposures.

Vomiting within 6 h of artefenomel dosing was recorded in around 25% of patients and had a substantial impact on artefenomel exposures (about 25% of that in non-vomiters), and to a lesser extent on ferroquine exposures (about 67–70% of that in non-vomiters). Variability in artefenomel exposures was also considerably higher in vomiters. Nausea and vomiting are known symptoms of malaria, especially in young children. It may be that in children who already felt unwell, a higher vomiting rate was the result of multiple factors including malaria symptoms, side-effect profile of artefenomel, and the need for large administration volumes for the exploratory artefenomel formulation. It is noteworthy that in a similar study of artefenomel (800 mg) plus piperaquine, which was ongoing at the same time as this study, a similar frequency of vomiting was detected [30]. For the lowest weight band, lower drug exposures were also evident in patients who did not vomit (although numbers were small). There were anecdotal reports that young children struggled to consume the entire dosing volume even though the vast majority of patients were recorded as having all drug administered (without considering vomiting). Considering these factors, the high administration volume very likely contributed to the high exposure variability and lower overall exposures in the youngest/lightest children.

Simple scatter plots of the relationship between exposure to both drugs and efficacy, with and without vomiting, showed that vomiting clearly decreased exposure. This lower exposure was associated with lower ACPR at Day 28.

To better understand the efficacy results, the dependency of efficacy on various covariates was explored using logistic regression. This analysis indicated that both drugs contributed to efficacy, with the odds of achieving ACPR at Day 28 increasing with increasing concentrations of both drugs, and that higher baseline parasitaemia reduced the odds of achieving PCR-adjusted ACPR at Day 28. No effect of age or sex could be identified, after taking drug exposure and baseline parasitaemia into account in the model. However, given the impact of baseline parasitaemia on efficacy, it is noteworthy that in this study, African patients ≤ 5 years of age had a median baseline parasitaemia around fivefold higher than African patients > 5 years of age (31,219 /µL vs 5962 /µL), and this could be related to the absence of, or the lower immunity in, young children. On average therefore, higher exposures would be required to achieve efficacy in African patients ≤ 5 years of age compared with African patients > 5 years of age.

The study results illustrate the challenges in developing anti-malarial drugs which require very high cure rates. Treatment doses need to be high enough to achieve the required duration of exposure across the population, whilst remaining well tolerated and safe. The administered treatment must be able to cure > 95% of the population including those with high baseline parasitaemia and with less sensitive parasites. The administered treatment must also accommodate multiple sources of exposure variability, including variable bioavailability and variability due to the need to scale doses across a large weight range using a feasible number of weight bands.

Clearly, low between-patient exposure variability including minimal food effects is key to maximizing the probability of achieving high efficacy, with as low a dose as possible and with acceptable tolerability. Achieving this balance is particularly challenging when attempting to administer the entire therapeutic dose in a single administration since this leads to a higher C_max_ compared with multiple-dose treatment.

For young children, single-dose treatments may be particularly challenging given that the full treatment must be administered whilst the patient has symptomatic malaria. Low dose/volume treatments will likely increase the feasibility of administration to young children, and this may be best achieved through developing potent drug combinations, which along with low between-patient exposure variability will provide feasible dose sizes.

Recruitment of Asian patients was stopped early by the DMC due to evidence of low efficacy. There were only 20 patients randomized, and these were all from one country, Vietnam, but efficacy was clearly substantially lower than in the African patients (20 to 40% at Day 28), across all ferroquine doses. Exposures of artefenomel, ferroquine and ferroquine active metabolite (SSR97213) were comparable in Asia and Africa. The logistic regression analysis confirmed that the response was significantly lower in Asian patients compared to African patients even when drug exposures and baseline parasitaemia were taken into account.

Initial parasite clearance was approximately twofold slower for Asian patients compared with African patients. However, 17/19 of these Asian patients carried the C580Y mutation, which was previously shown to reduce parasite clearance time [41]. These 17 patients showed a median PCt1/2 of 6.2 h, which is comparable to the 5.5 h reported for artefenomel in monotherapy studies [27]. The single evaluable patient carrying WT showed a PCt1/2 of 2.3 h, which is within the range previously described in monotherapy.

In recent years, there have been reports of *kelch-13* mutations potentially conferring artemisinin resistance arising in Africa [42]. In the present study, 10 out of 294 African patients (mITT, all ages) were found to carry 6 distinct *kelch-13* mutations, some of which were previously linked with artemisinin resistance [12]. The impact of *kelch-13* genotype on PCt1/2 for artefenomel could not be evaluated. The observed PCt1/2 of 2.6 h (range 1.3 to 6.6 h) is similar to the 3.6 h (95% CI 3.2 to 3.8 h) reported following artefenomel monotherapy in WT *P. falciparum* blood-stage malaria infection in healthy volunteers [43].

In the case of artemisinin combination therapies, frank treatment failure has only been evident where artemisinin resistance occurs alongside partner drug resistance [44]. It is therefore important to understand to what extent the treatment failure might be linked to ferroquine resistance. Ferroquine is a member of the 4-aminoquinoline family of anti-malarials, structurally related to piperaquine, amodiaquine and chloroquine. No ferroquine-resistant strains have been generated in the laboratory. It is fully active against chloroquine-sensitive and chloroquine-resistant *P. falciparum* [45]. Changes in piperaquine sensitivity have been reported to be linked to plasmepsin2/3 amplifications and *crt* mutations [46], although no correlation between minimum inhibitory concentration (MIC) shifts for piperaquine and ferroquine has been detected in field samples from Vietnam.

Recent studies comparing in vitro potency values of ferroquine determined on parasites from different countries in South-East Asia did not show any significant difference and less than a threefold decrease compared to laboratory strains. This suggests that no resistance to ferroquine has yet emerged in this region, which is well known to be the historical ignition point of resistance to anti-malarials.

The safety data demonstrated that vomiting was a significant tolerability issue with approximately 25% of patients vomiting during or shortly after artefenomel administration. Aside from the vomiting, the single-dose regimen of artefenomel + ferroquine was overall well tolerated in adults and children with uncomplicated *P. falciparum* malaria. The different doses of ferroquine tested appeared to have a similar overall tolerability and safety profile, although there was a trend towards a higher frequency of certain AEs, including the previously identified risks of proarrhythmic effect (QTc prolongation) [47] and hepatic effect (elevated liver enzymes) [48, 49], with the highest doses of ferroquine (the adult-equivalent doses of 900 and 1200 mg). Aside from a higher-than-expected frequency of vomiting post-dosing, the safety profile observed in this study was aligned with the known profiles of both drugs as observed in previous studies, with some findings overlapping with malaria symptoms.

## Conclusion

In this study, all treatment arms failed the protocol-defined adequate efficacy criterion for PCR-adjusted ACPR at Day 28 in the PP Set of African children ≤ 5 years of age. The highest dose used (equivalent to an adult dose of 1200 mg ferroquine plus 800 mg artefenomel) achieved only a 91.7% response from a single-dose treatment. Vomiting within 6 h of dosing occurred in around 25% of patients, reducing drug exposures and thus negatively impacting efficacy. The high variability of artefenomel exposure was also a key factor in the lack of success. New combination treatments with formulations requiring lower doses/dosing volumes for use in small children would clearly be an important next step for future development, and these aspects are being researched. Failures in Vietnam where the majority of parasites carry the *kelch-13* C580Y genotypes are particularly concerning. The influence of *kelch-13* genotype for African patients could not be evaluated because only a small fraction of the patients carried *kelch-13* mutations. Understanding the molecular basis for this difference in response will be important. Aside from the higher-than-expected incidence of vomiting shortly after artefenomel administration in the small children, no new safety signals were identified in this study. This study rules out the use of artefenomel and ferroquine as a single-dose cure. Successful multiple-day regimens with this combination may be possible but would offer little benefit over existing 3-day regimens of fully synthetic endoperoxides, such as arterolane-piperaquine. The lessons learned from this study will help influence the design of future development programs for next generation treatments for malaria which would simplify therapy and be effective against emerging strains of resistant parasites.

## Supplementary Information

**Additional file 1.** Study methods. Supplementary information regarding enrolment procedures, randomization, administration of study treatments, justification of the doses of ferroquine and artefenomel, dose adjustment to body weight, use of rescue treatment and definition of treatment failure.

**Additional file 2.** Study results. Supplementary data regarding patient demographics, baseline characteristics, compliance/exposure, as well as further efficacy and safety data.

**Additional file 3.** Pharmacokinetic analysis details. Supplementary document including tables and figures to provide further methodological details and results on the pharmacokinetic analysis.

**Additional file 4.** Exposure–response analysis details. Supplementary document including tables and figures to provide further methodological details and results on the exposure–response analysis.

**Additional file 5.** Electrocardiogram (ECG) exposure–response analysis details. Supplementary document including tables and figures to provide further methodological details and results on the ECG exposure–response analysis.

## Data Availability

The data sets used and/or analysed during the current study are available from the corresponding author on reasonable request.

## Abbreviations

ACPR: Adequate clinical and parasitological response
ACT: Artemisinin-based combination therapy
AE: Adverse event
AESI: Adverse event of special interest
ALT: Alanine aminotransferase
AST: Aspartate aminotransferase
AUC_(0-day28)_: Area under the curve from time 0 to Day 28
AUC_(0-inf)_: Area under the curve from time 0 to infinity
C_day7_: Concentration at Day 7 post-dose
CI: Confidence interval
CHIM: Controlled Human Infection Model
C_max_: Maximum concentration
CV: Coefficient of variation
DMC: Data Monitoring Committee
ECG: Electrocardiogram
FCT: Fever clearance time
GMR: Greater Mekong Region
IEC: Independent Ethics Committee
IWRS: Integrated web recognition system
MedDRA: Medical Dictionary for Regulatory Activities
MIC: Minimum inhibitory concentration (i.e. anti-malarial drug concentration associated with a multiplication rate of 1/asexual cycle)
mITT: Modified Intent-To-Treat
MMV: Medicines for Malaria Venture
OZ439: Artefenomel
PCR: Polymerase chain reaction
PCT: Parasite clearance time
PCt1/2: Parasite clearance half-life
PK: Pharmacokinetics
PP: Per-Protocol
PRR24: Parasite reduction ratio at 24 h
PRR48: Parasite reduction ratio at 48 h
QTc: Corrected QT interval
QTcB: Corrected QT interval using Bazett’s formula
QTcF: Corrected QT interval using Fridericia’s formula
SAE: Serious adverse event
TEAE: Treatment-emergent adverse event
ULN: Upper limit of normal
WHO: World Health Organization
WT: Wild type
WWARN: WorldWide Antimalarial Resistance Network

## References

[1] WHO. World Malaria Report 2019. Geneva: World Health Organization; 2019. https://www.who.int/publications/i/item/world-malaria-report-2019. Accessed 15 Dec 2020

[2] WHO. Guidelines for the treatment of malaria. 3rd Edn. World Health Organization; 2015. https://apps.who.int/iris/bitstream/handle/10665/162441/9789241549127_eng.pdf?sequence=1. Accessed 15 Dec 2020.

[3] Afaya A, Salia SM, Adatara P, Afaya RA, Suglo S, Japiong M (2018). Patients' knowledge of artemisinin-based combination therapy treatment and its impact on patient adherence. J Trop Med.

[4] Amponsah AO, Vosper H, Marfo AF (2015). Patient-related factors affecting adherence to antimalarial medication in an urban estate in Ghana. Malar Res Treat.

[5] Banek K, Lalani M, Staedke SG, Chandramohan D (2014). Adherence to artemisinin-based combination therapy for the treatment of malaria: a systematic review of the evidence. Malar J.

[6] Depoortere E, Guthmann JP, Sipilanyambe N, Nkandu E, Fermon F, Balkan S (2004). Adherence to the combination of sulphadoxine-pyrimethamine and artesunate in the Maheba refugee settlement Zambia. Trop Med Int Health.

[7] Banek K, Webb EL, Smith SJ, Chandramohan D, Staedke SG (2018). Adherence to treatment with artemether-lumefantrine or amodiaquine-artesunate for uncomplicated malaria in children in Sierra Leone: a randomized trial. Malar J.

[8] Beer N, Ali AS, Rotllant G, Abass AK, Omari RS, Al-mafazy AW (2009). Adherence to artesunate-amodiaquine combination therapy for uncomplicated malaria in children in Zanzibar Tanzania. Trop Med Int Health.

[9] Fogg C, Bajunirwe F, Piola P, Biraro S, Checchi F, Kiguli J (2004). Adherence to a six-dose regimen of artemether-lumefantrine for treatment of uncomplicated *Plasmodium falciparum* malaria in Uganda. Am J Trop Med Hyg.

[10] Kalyango JN, Rutebemberwa E, Karamagi C, Mworozi E, Ssali S, Alfven T (2013). High adherence to antimalarials and antibiotics under integrated community case management of illness in children less than five years in eastern Uganda. PLoS ONE.

[11] Ariey F, Witkowski B, Amaratunga C, Beghain J, Langlois AC, Khim N (2014). A molecular marker of artemisinin-resistant *Plasmodium falciparum* malaria. Nature.

[12] Ashley EA, Dhorda M, Fairhurst RM, Amaratunga C, Lim P, Suon S (2014). Spread of artemisinin resistance in *Plasmodium falciparum* malaria. N Engl J Med.

[13] Blasco B, Leroy D, Fidock DA (2017). Antimalarial drug resistance: linking *Plasmodium falciparum* parasite biology to the clinic. Nat Med.

[14] Wells TN, van Huijsduijnen R, Van Voorhis WC (2015). Malaria medicines: a glass half full?. Nat Rev Drug Discov..

[15] Uwimana A, Legrand E, Stokes BH, Ndikumana JM, Warsame M, Umulisa N (2020). Emergence and clonal expansion of in vitro artemisinin-resistant *Plasmodium falciparum* kelch13 R561H mutant parasites in Rwanda. Nat Med.

[16] Amaratunga C, Lim P, Suon S, Sreng S, Mao S, Sopha C (2016). Dihydroartemisinin-piperaquine resistance in *Plasmodium falciparum* malaria in Cambodia: a multisite prospective cohort study. Lancet Infect Dis.

[17] Duru V, Khim N, Leang R, Kim S, Domergue A, Kloeung N (2015). *Plasmodium falciparum* dihydroartemisinin-piperaquine failures in Cambodia are associated with mutant K13 parasites presenting high survival rates in novel piperaquine in vitro assays: retrospective and prospective investigations. BMC Med.

[18] Mukherjee A, Bopp S, Magistrado P, Wong W, Daniels R, Demas A (2017). Artemisinin resistance without pfkelch13 mutations in *Plasmodium falciparum* isolates from Cambodia. Malar J.

[19] Spring MD, Lin JT, Manning JE, Vanachayangkul P, Somethy S, Bun R (2015). Dihydroartemisinin-piperaquine failure associated with a triple mutant including kelch13 C580Y in Cambodia: an observational cohort study. Lancet Infect Dis.

[20] Hamilton WL, Amato R, van der Pluijm RW, Jacob CG, Quang HH, Thuy-Nhien NT (2019). Evolution and expansion of multidrug-resistant malaria in southeast Asia: a genomic epidemiology study. Lancet Infect Dis.

[21] Imwong M, Suwannasin K, Kunasol C, Sutawong K, Mayxay M, Rekol H (2017). The spread of artemisinin-resistant *Plasmodium falciparum* in the Greater Mekong subregion: a molecular epidemiology observational study. Lancet Infect Dis.

[22] Group WAbCTABS, Dahal P, d'Alessandro U, Dorsey G, Guerin PJ, Nsanzabana C, et al. Clinical determinants of early parasitological response to ACTs in African patients with uncomplicated falciparum malaria: a literature review and meta-analysis of individual patient data. BMC Med. 2015;13:212. 10.1186/s12916-015-0445-x PMC4561425 26343145

[23] Mathieu LC, Cox H, Early AM, Mok S, Lazrek Y, Paquet JC (2020). Local emergence in Amazonia of *Plasmodium falciparum* k13 C580Y mutants associated with in vitro artemisinin resistance. Elife.

[24] Miotto O, Sekihara M, Tachibana SI, Yamauchi M, Pearson RD, Amato R (2020). Emergence of artemisinin-resistant *Plasmodium falciparum* with kelch13 C580Y mutations on the island of New Guinea. PLoS Pathog.

[25] Burrows JN, Duparc S, Gutteridge WE, van Huijsduijnen R, Kaszubska W, Macintyre F (2017). New developments in anti-malarial target candidate and product profiles. Malar J..

[26] McCarthy JS, Ruckle T, Elliott SL, Ballard E, Collins KA, Marquart L (2019). A single-dose combination study with the experimental antimalarials artefenomel and DSM265 to determine safety and antimalarial activity against blood-stage *Plasmodium falciparum* in healthy volunteers. Antimicrob Agents Chemother.

[27] Phyo AP, Jittamala P, Nosten FH, Pukrittayakamee S, Imwong M, White NJ (2016). Antimalarial activity of artefenomel (OZ439), a novel synthetic antimalarial endoperoxide, in patients with *Plasmodium falciparum* and *Plasmodium vivax* malaria: an open-label phase 2 trial. Lancet Infect Dis.

[28] Charman SA, Arbe-Barnes S, Bathurst IC, Brun R, Campbell M, Charman WN (2011). Synthetic ozonide drug candidate OZ439 offers new hope for a single-dose cure of uncomplicated malaria. Proc Natl Acad Sci USA.

[29] Salim M, Khan J, Ramirez G, Murshed M, Clulow AJ, Hawley A (2019). Impact of ferroquine on the solubilization of artefenomel (OZ439) during in vitro lipolysis in milk and implications for oral combination therapy for malaria. Mol Pharm.

[30] Macintyre F, Adoke Y, Tiono AB, Duong TT, Mombo-Ngoma G, Bouyou-Akotet M (2017). A randomised, double-blind clinical phase II trial of the efficacy, safety, tolerability and pharmacokinetics of a single dose combination treatment with artefenomel and piperaquine in adults and children with uncomplicated *Plasmodium falciparum* malaria. BMC Med.

[31] Biot C, Nosten F, Fraisse L, Ter-Minassian D, Khalife J, Dive D (2011). The antimalarial ferroquine: from bench to clinic. Parasite.

[32] Biot C, Taramelli D, Forfar-Bares I, Maciejewski LA, Boyce M, Nowogrocki G (2005). Insights into the mechanism of action of ferroquine. Relationship between physicochemical properties and antiplasmodial activity. Mol Pharm..

[33] WHO. Management of severe malaria: a practical handbook. 3rd Edn. World Health Organization; 2013. https://apps.who.int/iris/bitstream/handle/10665/79317/9789241548526_eng.pdf?sequence=1. Accessed 15 Dec 2020.

[34] WHO. Methods and techniques for clinical trials on antimalarial drug efficacy: genotyping to identify parasite populations. Informal consultation organized by the Medicines for Malaria Venture and cosponsored by the World Health Organization. Amsterdam, 2017. http://apps.who.int/iris/bitstream/10665/43824/1/9789241596305_eng.pdf. Accessed 15 Dec 2020.

[35] WorldWide Antimalarial Resistance Network (WWARN). Parasite Clearance Estimator. https://www.wwarn.org/parasite-clearance-estimator-pce. Accessed 15 Dec 2020.

[36] Institut Pasteur (Authors: Menard D and Ariey F). PCR and sequencing for genotyping of candidate *Plasmodium falciparum* artemisinin resistance SNPs in the Kelch 13 gene. https://www.wwarn.org/tools-resources/procedures/pcr-and-sequencing-genotyping-candidate-plasmodium-falciparum-artemisinin. Accessed 15 Dec 2020.

[37] WHO. Methods for surveillance of antimalarial efficacy. Geneva, World Health Organization; 2009. http://apps.who.int/iris/bitstream/10665/44048/1/9789241597531_eng.pdf. Accessed 15 Dec 2020.

[38] Lee JJ, Liu DD (2008). A predictive probability design for phase II cancer clinical trials. Clin Trials.

[39] Monolix version 2019R1. Antony, France: Lixoft SAS, 2019. http://lixoft.com/products/monolix/. Accessed 15 Dec 2020.

[40] Modeling & Simulation in R - Supporting efficient model informed drug development with IQR tools. IntiQuan GmbH, Basel, Switzerland, 22 October 2020. https://iqrtools.intiquan.com/. Accessed 15 Dec 2020.

[41] WHO. Artemisinin resistance and artemisinin-based combination therapy efficacy - Global Malaria Programme, August 2018 Status Report. Geneva, World Health Organization, 2018. https://www.who.int/malaria/publications/atoz/artemisinin-resistance-august2018/en/. Accessed 15 Dec 2020.

[42] WHO. Artemisinin resistance and artemisinin-based combination therapy efficacy - Global Malaria Programme, December 2019 Status Report. Geneva, World Health Organization, 2019. https://www.who.int/docs/default-source/documents/publications/gmp/who-cds-gmp-2019-17-eng.pdf?ua=1. Accessed 15 Dec 2020.

[43] McCarthy JS, Baker M, O’Rourke P, Marquart L, Griffin P, van Huijsduijnen R (2016). Efficacy of OZ439 (artefenomel) against early *Plasmodium falciparum* blood-stage malaria infection in healthy volunteers. J Antimicrob Chemother..

[44] WHO. Q&A on artemisinin resistance. Geneva, World Health Organization, 2020. https://www.who.int/malaria/media/artemisinin_resistance_qa/en/. Accessed 15 Dec 2020.

[45] Dubar F, Khalife J, Brocard J, Dive D, Biot C (2008). Ferroquine, an ingenious antimalarial drug: thoughts on the mechanism of action. Molecules.

[46] van der Pluijm RW, Imwong M, Chau NH, Hoa NT, Thuy-Nhien NT, Thanh NV (2019). Determinants of dihydroartemisinin-piperaquine treatment failure in *Plasmodium falciparum* malaria in Cambodia, Thailand, and Vietnam: a prospective clinical, pharmacological, and genetic study. Lancet Infect Dis.

[47] Held J, Supan C, Salazar CL, Tinto H, Bonkian LN, Nahum A (2015). Ferroquine and artesunate in African adults and children with *Plasmodium falciparum* malaria: a phase 2, multicentre, randomised, double-blind, dose-ranging, non-inferiority study. Lancet Infect Dis.

[48] McCarthy JS, Ruckle T, Djeriou E, Cantalloube C, Ter-Minassian D, Baker M (2016). A Phase II pilot trial to evaluate safety and efficacy of ferroquine against early *Plasmodium falciparum* in an induced blood-stage malaria infection study. Malar J.

[49] Mombo-Ngoma G, Supan C, Dal-Bianco MP, Missinou MA, Matsiegui PB, Ospina Salazar CL (2011). Phase I randomized dose-ascending placebo-controlled trials of ferroquine–a candidate anti-malarial drug–in adults with asymptomatic Plasmodium falciparum infection. Malar J.

