# Supplementary material for: A randomized, double-blind, phase 2b study to investigate the efficacy, safety, tolerability and pharmacokinetics of a single-dose regimen of ferroquine with artefenomel in adults and children with uncomplicated Plasmodium falciparum malaria
Source: Malar J. 2021 May 19;20:222. doi: 10.1186/s12936-021-03749-4 (PMC8135182; doi:10.1186/s12936-021-03749-4)
Supplement: Supplementary file 1 — Additional file 1. Study methods. Supplementary information regarding enrolment procedures, randomization, administration of study treatments, justification of the doses of ferroquine and artefenomel, dose adjustment to body weight, use of rescue treatment and definition of treatment failure. [file 12936_2021_3749_MOESM1_ESM.pdf]

S1 Study methods - Supplementary details regarding the design of the study

Contents of the document

Enrolment procedures **2**

Randomization **2**

Figure S 1 Study age step-down and ferroquine step-up procedures 3

Administration of study treatments **4**

Justification of the doses of ferroquine and artefenomel **5**

Dose adjustment to body weight **6**

Table S 1 Weight bands and pediatric doses (mg) for artefenomel and ferroquine 6

Use of rescue treatment and definition of treatment failure **7**

References **8**

Enrolment procedures

Adults and children were included sequentially in 4 cohorts through a progressive age step-down procedure and ferroquine dose step-up procedure. In the first cohort, patients aged >14 years to <70 years were randomized. Thereafter, following the review of safety data by the independent Data Monitoring Committee (DMC), sequentially younger patients were recruited (Figure S 1). Dose groups could be dropped during the study following the DMC decision or for futility based on the outcome of pre-planned interim analyses.

Randomization

Eligible patients were centrally randomized to one of the possible treatment arms (artefenomel/ferroquine 800/400 mg, 800/600 mg, 800/900 mg, and 800/1200 mg) via an Integrated Web Recognition System (IWRS) using permuted block randomization schedules and 3 randomization lists (see Figure S 1 for further details on allocation ratios). Pre-defined data from patients aged >14 years and weighing ≥35 kg exposed to the 3 lower doses in parallel were reviewed (Cohort 1a, including ≥10 patients per treatment arm) before testing 1200 mg ferroquine dose in association with 800 mg artefenomel (Cohort 1b). Randomization was stratified by region, and within Africa, by age class (>14 to <70 years; >5 to ≥14 years; >2 to ≤5 years; >6 months to ≤2 years). Following the decision to stop recruitment in Asia due to low efficacy, the stratification by region was no longer relevant after the second cohort (>5 to ≥14 years). Following the age step-down and ferroquine dose step-up procedures, the randomization across treatment arms was balanced.

Figure S 1 Study age step-down and ferroquine step-up procedures

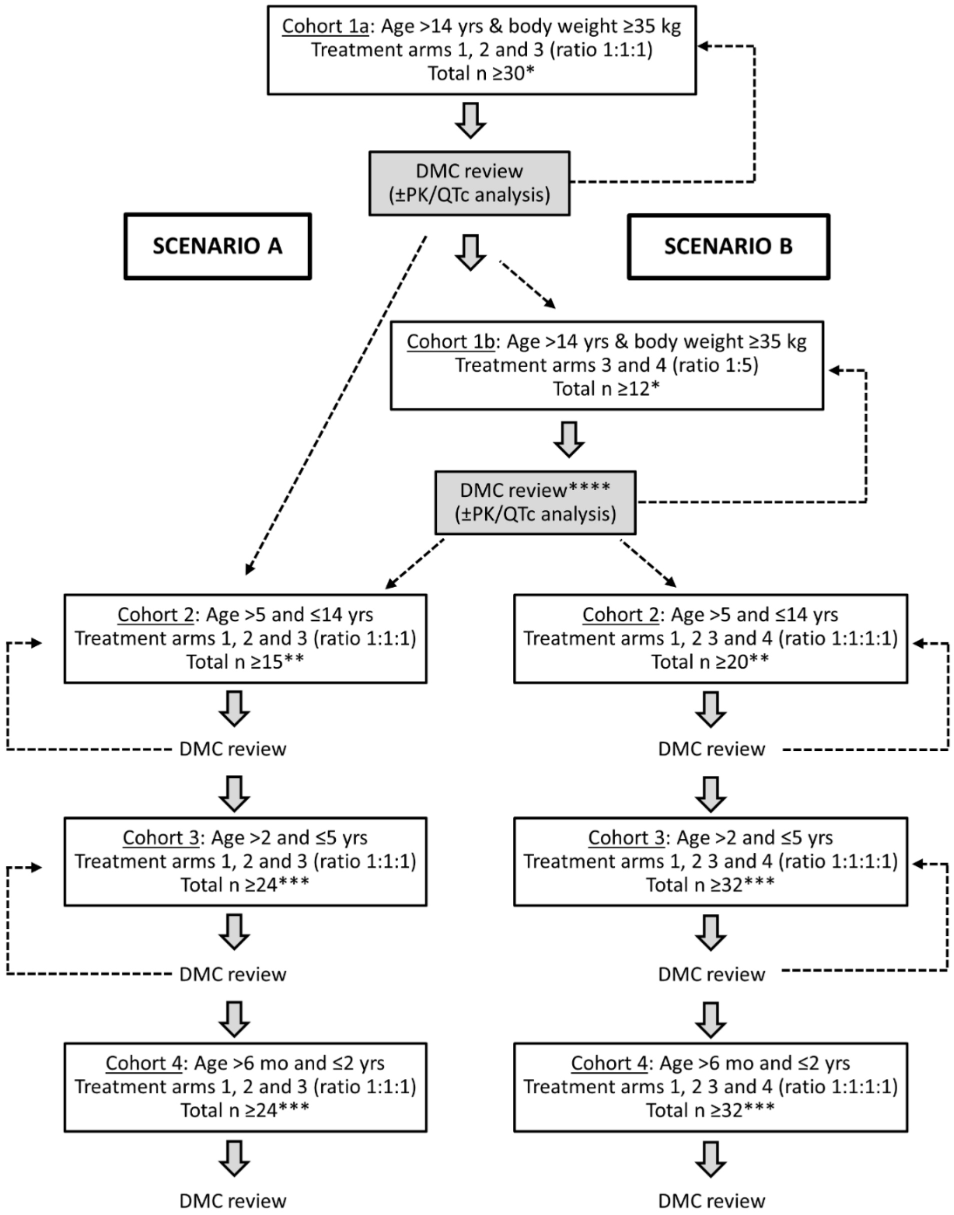

mo = months

* A minimum number of 10 patients (Cohort 1) per treatment arm for DMC safety and PK/QTc evaluation.

** A minimum number of 5 patients (Cohort 2) per treatment arm for DMC review.

*** A minimum number of 8 patients (Cohorts 3 and 4) per treatment arm for DMC review.

After all these age step-down stages, the recruitment was to be fully open. Approximately 62 patients were to be recruited in Cohorts 1 and 2 for Scenario B. The remaining patients were to comprise Cohorts 3 and 4. At least 10% of the population of African patients aged ≤2 years were to come from Cohort 4.

**** DMC review before starting Cohort 2 recruitment were to be made on pooled data (Cohorts 1a and 1b) with a total number of patients N ≥42.

Note on the randomization process:

Eligible patients were centrally randomized to 1 of the possible treatment arms, as detailed below, via IWRS using permuted block randomization schedules. The IWRS managed 3 patient randomization lists (1 for Cohort 1a, a 2nd for Cohort 1b, and the 3rd including all 4 cohorts) and allocated the treatment number and the corresponding treatment kit to the patient as follows:

- In Cohort 1a, 30 patients were to be randomized to 1 of the 3 treatment arms of artefenomel/ferroquine. The randomization ratio was 1:1:1 (artefenomel/ferroquine 800/400 mg: artefenomel/ferroquine 800/600 mg: artefenomel/ferroquine 800/900 mg).
- In Cohort 1b, if the DMC had given the green light to proceed with the 1200 mg dose of ferroquine, 12 patients were to be randomized to 1 of the 2 treatment arms of artefenomel/ferroquine. The randomization ratio was 1:5 (artefenomel/ferroquine 800/900 mg: artefenomel/ferroquine 800/1200 mg).
- After DMC review of Cohorts 1a and 1b, if the DMC had given the green light to proceed with the 1200 mg dose of ferroquine in younger patients according to the age step-down procedure, approximately 155 patients (150 African patients ≤5 years of age [Cohorts 3 and 4] + a minimum of 5 African patients aged >5 years to ≤14 years [Cohort 2]) were to be randomized to 1 of the 4 treatment arms of artefenomel/ferroquine according to the randomization ratio of 1:1:1:1 (artefenomel/ferroquine 800/400 mg: artefenomel/ferroquine 800/600 mg: artefenomel/ferroquine 800/900 mg: artefenomel/ferroquine 800/1200 mg).

Administration of study treatments

Exploratory formulations of both ferroquine and artefenomel were administered orally in the fasting condition (3 h before and 2 h after completion of administration) by a healthcare

worker. Ferroquine capsules were administered first, in a double-blind manner: patients in each weight band received the same number of capsules (6 or 8 capsules of ferroquine 5, 30, 100 and/or 200 mg or matching placebo). Ferroquine capsules could be opened, and a solution prepared for young children. Immediately after ferroquine administration, artefenomel was administered as a suspension formulation containing alpha-tocopherol polyethylene glycol 1000 succinate, in an unblinded manner: artefenomel was formulated as a sachet of granules containing the appropriate dosage for the treatment arm and weight band. If a solution of ferroquine was prepared, it had to be administered within 30 min of preparation. After preparation of the artefenomel suspension, the mixture needed to stand for 20-30 min, and so was prepared in advance. Artefenomel was to be administered within 2 h 20 min after the start of preparation of the suspension.

Justification of the doses of ferroquine and artefenomel

A single dose of 800 mg artefenomel was considered the maximum well-tolerated dose. Efficacy was demonstrated (up to 36 h) against *P. falciparum* and *P. vivax* malaria in a proof-of-concept study in adult Asian patients, with a 97.9% reduction in parasites for all doses investigated (200-1200 mg). Exposure was predicted not to exceed that achieved in previous studies, in which artefenomel had been well tolerated. Since artefenomel had not been studied in a pediatric population, the pediatric dose adjustments were based on simulation (see the dose adjustment section below). The ferroquine doses were selected to span a range of PCR-adjusted ACPR at Day 28, estimated based on the results of a phase 2b study that tested 100 mg, 200 mg and 300 mg of ferroquine in combination with 200 mg artesunate, and 200 mg ferroquine alone, administered once per day for 3 days to adults and

children [Held 2015]. The highest ferroquine dose was estimated based on predicted effect on corrected QT interval (QTc) prolongation.

Dose adjustment to body weight

Patients weighing <35 kg received doses that were adjusted to body weight. The doses of ferroquine were selected using a population PK model including data of pediatric patients down to 15 kg, such that the lightest subject of a given weight band would not have exposures higher than the lightest adult (35 kg), and that a fixed artefenomel/ferroquine dose ratio would be maintained within each weight band (see Table S 1). The calculated ferroquine 1200 mg dose-equivalent was 300 mg for patients weighing ≥7 to <10 kg. The doses of artefenomel were selected such that the heaviest patient in each weight band would achieve similar exposures to a 60 kg adult, and the lightest patient would not exceed the exposures of the lightest adult (35 kg) in the same treatment arm. The calculated artefenomel 800 mg dose-equivalent was 200 mg for patients weighing ≥7 to <10 kg.

Table S 1 Weight bands and pediatric doses (mg) for artefenomel and ferroquine

		**Artefenomel Doses**	**Ferroquine Doses Corresponding to the Different Treatment Arms**			
**Body Weight Band**	**Factor[a]**	**(mg)**	**(mg)**	**(mg)**	**(mg)**	**(mg)**
**≥35 kg (adult-equivalent dose)**	1	800	400	600	900	1200
**≥24 kg and <35 kg**	0.75	600	300	450	675	900
**≥15 kg and <24 kg**	0.5	400	200	300	450	600
**≥10 kg and <15 kg**	0.37	300	150	225	335	450
**≥7 kg and <10 kg**	0.25	200	100	150	225	300
**≥5 kg and <7 kg**	0.19	150	75	115	170	225
**Ferroquine/artefenomel ratio**			0.50	0.75	1.13	1.50

a. The factor is the ratio to the adult-equivalent doses.
Ferroquine doses were selected using a population PK model including data of paediatric patients down to 15 kg.
Artefenomel dose for a given weight band was adjusted assuming clearance (mainly hepatic) and volume of distribution are related to body weight using the theoretic allometric exponents (0.75 and 1, respectively).

Use of rescue treatment and definition of treatment failure

The use of established anti-malarial drug combination per WHO recommendations [WHO 2009] or local guidelines was to be considered as a rescue treatment and the choice of the best therapeutic option was at the Investigator's discretion.

The use of a rescue treatment was recommended in the following cases:

- Vomiting during or after FQ dosing or vomiting within 35 min after OZ439 re-dosing.
- Treatment failure as defined below.

The classification of treatment outcomes was adapted from the WHO 2009 criteria [WHO 2009]. Treatment failure was defined as patients who met any of the criteria for early treatment failure, late clinical failure or late parasitological failure, as defined below.

Early treatment failure (Day 1 to 3), any of the following:

- Danger signs or severe malaria at Day 1, 2 or 3 in the presence of parasitaemia.
- Parasite count at Day 2 higher than at Day 0, irrespective of axillary temperature.
- Parasitaemia at Day 3 with axillary temperature ≥37.5°C.
- Parasite count at Day 3 ≥25% at Day 0.

Late clinical failure (Day 4 to 63), any of the following:

- Danger signs or severe malaria in the presence of parasitaemia on any day between Day 4 and Day 63 in patients who did not previously meet any of the criteria of early treatment failure.
- Presence of parasitaemia on any day between Day 4 and Day 63 with axillary temperature ≥37.5°C (or history of fever) in patients who did not previously meet any of the criteria of early treatment failure.

Late parasitological failure:

- Presence of parasitaemia on any day between Day 7 and Day 63 and axillary temperature <37.5°C in patients who did not previously meet any of the criteria of early treatment failure or late clinical failure.

References

[Held 2015] Held J, Supan C, Salazar CL, Tinto H, Bonkian LN, Nahum A, Moulero B, Sie A, Coulibaly B, Sirima SB, et al: Ferroquine and artesunate in African adults and children with Plasmodium falciparum malaria: a phase 2, multicentre, randomised, double-blind, dose-ranging, non-inferiority study. Lancet Infect Dis 2015, 15:1409-1419.

[WHO 2009] World Health Organization (WHO). Methods for surveillance of antimalarial drug efficacy, WHO, 2009, ISBN 978 92 4 159753 1.
