# Supplementary material for: A randomized, double-blind, phase 2b study to investigate the efficacy, safety, tolerability and pharmacokinetics of a single-dose regimen of ferroquine with artefenomel in adults and children with uncomplicated Plasmodium falciparum malaria
Source: Malar J. 2021 May 19;20:222. doi: 10.1186/s12936-021-03749-4 (PMC8135182; doi:10.1186/s12936-021-03749-4)
Supplement: Supplementary file 2 — Additional file 2. Study results. Supplementary data regarding patient demographics, baseline characteristics, compliance/exposure, as well as further efficacy and safety data. [file 12936_2021_3749_MOESM2_ESM.pdf]

S2 Study results - Supplementary results

Contents of the document

Patient demographics and baseline characteristics 3

Table S 2 Demographics and patient characteristics of the study population (randomized patients) 3

Compliance/exposure 4

Table S 3 Exposure to ferroquine and artefenomel and incidence of vomiting (Safety Set) 4

ACPR in African patients ≤5 years 6

Table S 4 PCR-adjusted ACPR at Day 28 according to baseline parasitaemia quartile in African patients aged ≤5 years (PP Set) 6

Table S 5 Crude ACPR at Day 28 according to age group and body weight band in African patients aged ≤5 years (PP Set) 7

Table S 6 Crude ACPR at Day 28, 42 and 63 in African patients aged ≤5 years (mITT Set) 8

Table S 7 Time to re-emergence, recrudescence and re-infection (Kaplan-Meier estimates) in African patients aged ≤5 years (mITT Set) 9

ACPR in African patients >5 years and Asian patients 10

Table S 8 Crude and PCR-adjusted ACPR at Day 28, 42 and 63 in African patients aged >5 years and Asian patients (PP Sets) 10

Table S 9 Time to re-emergence, recrudescence and re-infection (Kaplan-Meier estimates) in Asian patients (Asian mITT Set) 12

Table S 10 Crude ACPR at Day 28, 42 and 63 in African patients aged >5 years and Asian patients (mITT Sets) 13

Parasite clearance kinetics 14

Table S 11 Parasite clearance time (Kaplan-Meier estimates) by region (PP Sets) 14

Table S 12 Parasite clearance parameters by region (PP Sets) 15

Fever clearance time (FCT) 16

Time to gametocyte appearance/clearance 17

Safety and tolerability........ 18

Table S 13 Overview of adverse event profile: Treatment-emergent adverse events (Safety Set) 18

Table S 14 Summary of treatment-emergent adverse events reported in ≥5% of the African patients aged ≤5 years in any treatment arm (Safety Set, African patients ≤5 years) 19

Patient demographics and baseline characteristics

Table S 2 Demographics and patient characteristics of the study population (randomized patients)

	Artefenomel mg: Ferroquine mg				
	**800:400**	**800:600**	**800:900**	**800:1200**	**Total**
**All patients**					
N	93	94	97	93	377
Males, n (%)	56 (60.2)	48 (51.1)	54 (55.7)	37 (39.8)	195 (51.7)
Age (years), median (range)	3.89 (0.8; 56.0)	3.83 (0.7; 61.9)	3.65 (0.9; 55.8)	4.13 (0.6; 53.4)	3.89 (0.6; 61.9)
>6 months to ≤2 years, n (%)	7 (7.5)	8 (8.5)	6 (6.2)	7 (7.5)	28 (7.4)
>2 years to ≤5 years, n (%)	66 (71.0)	66 (70.2)	67 (69.1)	65 (69.9)	264 (70.0)
>5 years to ≤14 years, n (%)	6 (6.5)	5 (5.3)	6 (6.2)	5 (5.4)	22 (5.8)
>14 years to ≤18 years, n (%)	6 (6.5)	5 (5.3)	7 (7.2)	4 (4.3)	22 (5.8)
>18 years	8 (8.6)	10 (10.6)	11 (11.3)	12 (12.9)	41 (10.9)
Body weight (kg), median (range)	14.00 (8.2; 70.2)	14.65 (8.0; 77.3)	14.30 (7.7; 89.0)	14.60 (8.1; 75.0)	14.40 (7.7; 89.0)
Baseline parasitaemia (/µL), median (range)	25011.0 (18; 182719)	29142.0 (550; 177867)	26485.0 (599; 145053)	21455.0 (920; 119804)	25392.5 (18; 182719)

N: total number of patients in the relevant analysis set. All doses of artefenomel and ferroquine are expressed as adult-equivalent doses.

Compliance/exposure

Table S 3 Exposure to ferroquine and artefenomel and incidence of vomiting (Safety Set)

	Artefenomel mg: Ferroquine mg				
	**800:400**	**800:600**	**800:900**	**800:1200**	**Total**
Number of patients in Safety Set	92	94	96	91	373
**Exposure to ferroquine**					
N	92	94	96	91	373
Ferroquine administration method, n (%)					
Capsules	17 (18.5)	16 (17.0)	19 (19.8)	16 (17.6)	68 (18.2)
Suspension	75 (81.5)	78 (83.0)	77 (80.2)	75 (82.4)	305 (81.8)
Total/partial ferroquine administration, n (%)					
Full dose administered	92 (100)	94 (100)	96 (100)	90 (98.9)	372 (99.7)
Full dose not administered[a]	0	0	0	1 (1.1)	1 (0.3)[b]
Vomiting during or after ferroquine administration, but before artefenomel administration, n (%)					
No	90 (97.8)	93 (98.9)	93 (96.9)	90 (98.9)	366 (98.1)
Yes[c]	2 (2.2)	1 (1.1)	3 (3.1)	1 (1.1)	7 (1.9)
**Exposure to artefenomel**					
**Artefenomel dosing**					
Artefenomel not administered, n (%)	2 (2.2)	1 (1.1)	3 (3.1)	1 (1.1)	7 (1.9)
Total/partial artefenomel administration, n (%)					
N	90	93	93	90	366
Full dose administered	90 (100)	92 (98.9)	91 (97.8)	88 (97.8)	361 (98.6)
Full dose not administered[a]	0	1 (1.1)	2 (2.2)	2 (2.2)	5 (1.4)[d]
Vomiting after artefenomel administration, n (%)					
No	69 (76.7)	70 (75.3)	69 (74.2)	68 (75.6)	276 (75.4)
Yes	21 (23.3)	23 (24.7)	24 (25.8)	22 (24.4)	90 (24.6)
If yes, timing of vomiting[e]					
<5 min	3 (14.3)	0	1 (4.2)	2 (9.1)	6 (6.7)
5-35 min	11 (52.4)	14 (60.9)	14 (58.3)	9 (40.9)	48 (53.3)
>35 min	7 (33.3)	9 (39.1)	9 (37.5)	11 (50.0)	36 (40.0)

	Artefenomel mg: Ferroquine mg				
	800:400	800:600	800:900	800:1200	Total
**Artefenomel re-dosing**[f]					
Total/partial artefenomel re-administration, n (%)					
N	3	0	2	1	6
Full dose administered	2 (66.7)	0	2 (100)	1 (100)	5 (83.3)
Full dose not administered[a]	1 (33.3)	0	0	0	1 (16.7)[g]
Vomiting after artefenomel re-administration, n (%)					
N	3	0	2	1	6
No	1 (33.3)	0	2 (100)	1 (100)	4 (66.7)
Yes	2 (66.7)	0	0	0	2 (33.3)
If yes, timing of vomiting[e]					
<5 min	1 (50.0)	0	0	0	1 (50.0)
5-35 min	0	0	0	0	0
>35 min	1 (50.0)	0	0	0	1 (50.0)

a. The item corresponds to the question 'was all study drug administered?' in the Case Report Form.

b. One patient did not receive the full dose of ferroquine. The reason for partial administration of ferroquine was: 'other reason' (vomiting before completing the entire dose of ferroquine). This patient is recorded as one of the patients who vomited during or after ferroquine administration, but before artefenomel administration.

c. If patients vomited during or after ferroquine administration but before artefenomel administration, no re-dosing of ferroquine was to be performed. The patients received rescue treatment and were discontinued from the study. These patients were followed-up for safety.

d. The reasons for partial administration of artefenomel were: 'AE' (vomiting, n=4), 'other reason' (vomiting <5 min after artefenomel administration, but the patient was also reported with an AE of vomiting on the same day, n=1).

e. Percentages are based on patients who vomited. Investigators were instructed not to report vomiting occurring more than 6 h after the start of artefenomel administration or re-dosing.

f. Patients who vomited within 5 min of the start of artefenomel administration were to be re-dosed. Patients who vomited from 5 min after the start of artefenomel administration continued to take the artefenomel dose (if there was any left) but were not to be re-dosed.

g. The reason for partial administration of artefenomel was: 'AE'.

Note: occurrence and timing of vomiting was recorded for up to 6 h after study drug administration on the Case Report Form page dedicated to the extent of study drug exposure and compliance, whereas AEs of vomiting were recorded up to Day 63 on the Case Report Form page dedicated to the AEs.

All doses of artefenomel and ferroquine are expressed as adult-equivalent doses.

ACPR in African patients ≤5 years

Table S 4 PCR-adjusted ACPR at Day 28 according to baseline parasitaemia quartile in African patients aged ≤5 years (PP Set)

	Artefenomel mg: Ferroquine mg				
	800:400	800:600	800:900	800:1200	Total
**≤Q1 (9082/µL)**					
N	14	11	13	19	57
PCR-adjusted ACPR, n/r (%)	12/14 (85.7)	8/11 (72.7)	12/13 (92.3)	18/19 (94.7)	50/57 (87.7)
[95% CI]	[57.2;98.2]	[39.0;94.0]	[64.0;99.8]	[74.0;99.9]	[76.3;94.9]
**>Q1 (9082/µL) to ≤Q2 (31219/µL)**					
N	9	15	16	16	56
PCR-adjusted ACPR, n/r (%)	7/9 (77.8)	14/15 (93.3)	16/16 (100.0)	14/16 (87.5)	51/56 (91.1)
[95% CI]	[40.0;97.2]	[68.1;99.8]	[79.4;100.0]	[61.7;98.4]	[80.4;97.0]
**>Q2 (31219/µL) to ≤Q3 (64395/µL)**					
N	15	15	15	13	58
PCR-adjusted ACPR, n/r (%)	11/15 (73.3)	12/15 (80.0)	13/15 (86.7)	12/13 (92.3)	48/58 (82.8)
[95% CI]	[44.9;92.2]	[51.9;95.7]	[59.5;98.3]	[64.0;99.8]	[70.6;91.4]
**>Q3 (64395/µL)**					
N	13	19	13	12	57
PCR-adjusted ACPR, n/r (%)	10/13 (76.9)	17/19 (89.5)	10/13 (76.9)	11/12 (91.7)	48/57 (84.2)
[95% CI]	[46.2;95.0]	[66.9;98.7]	[46.2;95.0]	[61.5;99.8]	[72.1;92.5]

N: total number of patients in the relevant analysis set. All doses of artefenomel and ferroquine are expressed as adult-equivalent doses.
n: number of patients in each category achieving ACPR.
r: total number of patients in the relevant analysis set with a defined response of Cure or Failure (i.e. patients evaluable for the outcome considered).

Table S 5 Crude ACPR at Day 28 according to age group and body weight band in African patients aged ≤5 years (PP Set)

	Artefenomel mg: Ferroquine mg				
	800:400	800:600	800:900	800:1200	Total
**Crude ACPR at Day 28 by age group**					
**>6 months to ≤2 years**					
N	7	8	6	7	28
Crude ACPR, n/r (%)	3/7 (42.9)	6/8 (75.0)	5/6 (83.3)	4/7 (57.1)	18/28 (64.3)
[95% CI]	[9.9;81.6]	[34.9;96.8]	[35.9;99.6]	[18.4;90.1]	[44.1;81.4]
**>2 years to ≤5 years**					
N	62	59	57	55	233
Crude ACPR, n/r (%)	31/62 (50.0)	42/59 (71.2)	43/57 (75.4)	50/55 (90.9)	166/233 (71.2)
[95% CI]	[37.0;63.0]	[57.9;82.2]	[62.2;85.9]	[80.0;97.0]	[65.0;77.0]
**Crude ACPR at Day 28 by body weight band**					
**≥7 kg to <10 kg**					
N	6	7	6	9	28
Crude ACPR, n/r (%)	1/6 (16.7)	5/7 (71.4)	3/6 (50.0)	5/9 (55.6)	14/28 (50.0)
[95% CI]	[0.4;64.1]	[29.0;96.3]	[11.8;88.2]	[21.2;86.3]	[30.6;69.4]
**≥10 kg to <15 kg**					
N	46	37	45	31	159
Crude ACPR, n/r (%)	23/46 (50.0)	26/37 (70.3)	33/45 (73.3)	27/31 (87.1)	109/159 (68.6)
[95% CI]	[34.9;65.1]	[53.0;84.1]	[58.1;85.4]	[70.2;96.4]	[60.7;75.7]
**≥15 kg to <24 kg**					
N	17	23	12	22	74
Crude ACPR, n/r (%)	10/17 (58.8)	17/23 (73.9)	12/12 (100.0)	22/22 (100.0)	61/74 (82.4)
[95% CI]	[32.9;81.6]	[51.6;89.8]	[73.5;100.0]	[84.6;100.0]	[71.8;90.3]

N: total number of patients in the relevant analysis set. All doses of artefenomel and ferroquine are expressed as adult-equivalent doses.
n: number of patients in each category achieving ACPR.
r: total number of patients in the relevant analysis set with a defined response of Cure or Failure (i.e. patients evaluable for the outcome considered).

Table S 6 Crude ACPR at Day 28, 42 and 63 in African patients aged ≤5 years (mITT Set)

	Artefenomel mg: Ferroquine mg				
	**800:400**	**800:600**	**800:900**	**800:1200**	**Total**
**Day 28**					
N	69	73	70	69	281
Crude ACPR, n/r (%)	34/69 (49.3)	50/73 (68.5)	52/70 (74.3)	56/69 (81.2)	192/281 (68.3)
[95% CI]	[37.0; 61.6]	[56.6; 78.9]	[62.4; 84.0]	[69.9; 89.6]	[62.5; 73.7]
**Day 42**					
N	69	73	70	69	281
Crude ACPR, n/r (%)	27/69 (39.1)	35/73 (47.9)	41/70 (58.6)	43/69 (62.3)	146/281 (52.0)
[95% CI]	[27.6; 51.6]	[36.1; 60.0]	[46.2; 70.2]	[49.8; 73.7]	[45.9; 57.9]
**Day 63**					
N	69	73	70	69	281
Crude ACPR, n/r (%)	25/69 (36.2)	32/73 (43.8)	38/70 (54.3)	36/69 (52.2)	131/281 (46.6)
[95% CI]	[25.0; 48.7]	[32.2; 55.9]	[41.9; 66.3]	[39.8; 64.4]	[40.7; 52.6]

N: total number of patients in the relevant analysis set. All doses of artefenomel and ferroquine are expressed as adult-equivalent doses.
n: number of patients in each category achieving ACPR.
r: total number of patients in the relevant analysis set with a defined response of Cure or Failure (i.e. patients evaluable for the outcome considered).

Table S 7 Time to re-emergence, recrudescence and re-infection (Kaplan-Meier estimates) in African patients aged ≤5 years (mITT Set)

	Artefenomel mg: Ferroquine mg				
	**800:400**	**800:600**	**800:900**	**800:1200**	**Total**
**Time to re-emergence**					
N	69	73	70	69	281
Number of events, n (%)	44 (63.8)	38 (52.1)	28 (40.0)	31 (44.9)	141 (50.2)
Number of censored, n (%)	25 (36.2)	35 (47.9)	42 (60.0)	38 (55.1)	140 (49.8)
Time to re-emergence (days)	36.0	61.0	NE	64.0	63.0
[95% CI]	[24.0; 63.0]	[43.0; NE]	NE	[63.0; 65.0]	[56.0; 65.0]
**Time to recrudescence**					
N	69	73	70	69	281
Number of events, n (%)	14 (20.3)	9 (12.3)	7 (10.0)	7 (10.1)	37 (13.2)
Number of censored, n (%)	55 (79.7)	64 (87.7)	63 (90.0)	62 (89.9)	244 (86.8)
Time to re-emergence (days)	NE	NE	NE	NE	NE
[95% CI]	NE	NE	NE	NE	NE
**Time to re-infection**					
N	69	73	70	69	281
Number of events, n (%)	26 (37.7)	25 (34.2)	19 (27.1)	18 (26.1)	88 (31.3)
Number of censored, n (%)	43 (62.3)	48 (65.8)	51 (72.9)	51 (73.9)	193 (68.7)
Time to re-emergence (days)	NE	NE	NE	65.0	65.0
[95% CI]	NE	NE	NE	NE	NE

NE: not estimated.
All doses of artefenomel and ferroquine are expressed as adult-equivalent doses.
Note: Patients with no event were censored at the time of study completion, premature study discontinuation, including switch to established anti-malarial treatment or start of any other treatment with anti-malarial activity, whichever was earliest.
Re-emergence/Recurrence (recrudescence and re-infection): The appearance of asexual parasites after clearance of initial infection irrespective of genotype.
Recrudescence: The appearance of asexual parasites after clearance of initial infection with a genotype identical to that of parasites present at baseline. Recrudescence was confirmed by microscopy (positive blood smear) and by genotyping PCR analysis.
Re-infection: The appearance of asexual parasites after clearance of initial infection with a genotype that differed from that of parasites present at baseline. Re-infection was confirmed by microscopy (positive blood smear) and by genotyping PCR analysis. Confirmed new infection was not regarded as treatment failure or recrudescence.

ACPR in African patients >5 years and Asian patients

Table S 8 Crude and PCR-adjusted ACPR at Day 28, 42 and 63 in African patients aged >5 years and Asian patients (PP Sets)

	Artefenomel mg: Ferroquine mg				
	**800:400**	**800:600**	**800:900**	**800:1200**	**Total**
**African patients >5 years**					
**Day 28**					
N	11	11	12	10	44
Crude ACPR, n/r (%)	7/11 (63.6)	10/11 (90.9)	11/12 (91.7)	9/10 (90.0)	37/44 (84.1)
[95% CI]	[30.8; 89.1]	[58.7; 99.8]	[61.5; 99.8]	[55.5; 99.7]	[69.9; 93.4]
PCR-adjusted ACPR, n/r (%)	8/11 (72.7)	10/10 (100)	12/12 (100)	9/10 (90.0)	39/43 (90.7)
[95% CI]	[39.0; 94.0]	[69.2; 100]	[73.5; 100]	[55.5; 99.7]	[77.9; 97.4]
**Day 42**					
N	10	10	11	10	41
Crude ACPR, n/r (%)	5/10 (50.0)	4/10 (40.0)	9/11 (81.8)	8/10 (80.0)	26/41 (63.4)
[95% CI]	[18.7; 81.3]	[12.2; 73.8]	[48.2; 97.7]	[44.4; 97.5]	[46.9; 77.9]
PCR-adjusted ACPR, n/r (%)	5/8 (62.5)	7/7 (100)	10/10 (100)	9/10 (90.0)	31/35 (88.6)
[95% CI]	[24.5; 91.5]	[59.0; 100]	[69.2; 100]	[55.5; 99.7]	[73.3; 96.8]
**Day 63**					
N	8	10	11	10	39
Crude ACPR, n/r (%)	2/8 (25.0)	3/10 (30.0)	5/11 (50.0)	6/10 (60.0)	16/39 (42.1)
[95% CI]	[3.2; 65.1]	[6.7; 65.2]	[18.7; 81.3]	[26.2; 87.8]	[26.3; 59.2]
PCR-adjusted ACPR, n/r (%)	2/6 (33.3)	4/4 (100)	7/8 (87.5)	7/9 (77.8)	20/27 (74.1)
[95% CI]	[4.3; 77.7]	[39.8; 100]	[47.3; 99.7]	[40.0; 97.2]	[53.7; 88.9]

	Artefenomel mg: Ferroquine mg				
	**800:400**	**800:600**	**800:900**	**800:1200**	**Total**
**Asian patients**					
**Day 28**					
N	4	5	5	5	19
Crude ACPR, n/r (%)	1/4 (25.0)	1/5 (20.0)	2/5 (40.0)	1/5 (20.0)	5/19 (26.3)
[95% CI]	[0.6; 80.6]	[0.5; 71.6]	[5.3; 85.3]	[0.5; 71.6]	[9.1; 51.2]
PCR-adjusted ACPR, n/r (%)	1/4 (25.0)	1/4 (25.0)	2/5 (40.0)	1/5 (20.0)	5/18 (27.8)
[95% CI]	[0.6; 80.6]	[0.6; 80.6]	[5.3; 85.3]	[0.5; 71.6]	[9.7; 53.5]
**Day 42**					
N	4	5	5	5	19
Crude ACPR, n/r (%)	1/4 (25.0)	1/5 (20.0)	2/5 (40.0)	1/5 (20.0)	5/19 (26.3)
[95% CI]	[0.6; 80.6]	[0.5; 71.6]	[5.3; 85.3]	[0.5; 71.6]	[9.1; 51.2]
PCR-adjusted ACPR, n/r (%)	1/4 (25.0)	1/4 (25.0)	2/5 (40.0)	1/5 (20.0)	5/18 (27.8)
[95% CI]	[0.6; 80.6]	[0.6; 80.6]	[5.3; 85.3]	[0.5; 71.6]	[9.7; 53.5]
**Day 63**					
N	3	5	5	5	18
Crude ACPR, n/r (%)	0/3	1/5 (20.0)	2/5 (40.0)	1/5 (20.0)	4/18 (22.2)
[95% CI]	[-; -]	[0.5; 71.6]	[5.3; 85.3]	[0.5; 71.6]	[6.4; 47.6]
PCR-adjusted ACPR, n/r (%)	0/3	1/4 (25.0)	2/5 (40.0)	1/5 (20.0)	4/17 (23.5)
[95% CI]	[-; -]	[0.6; 80.6]	[5.3; 85.3]	[0.5; 71.6]	[6.8; 49.9]

All doses of artefenomel and ferroquine are expressed as adult-equivalent doses.
N: total number of patients in the relevant analysis set.
n: number of patients in each category achieving ACPR.
r: total number of patients in the relevant analysis set with a defined response of Cure or Failure (i.e. patients evaluable for the outcome considered).

Table S 9 Time to re-emergence, recrudescence and re-infection (Kaplan-Meier estimates) in Asian patients (Asian mITT Set)

	Artefenomel mg: Ferroquine mg				
	800:400	800:600	800:900	800:1200	Total
**Time to re-emergence**					
N	4	5	6	5	20
Number of events, n (%)	3 (75.0)	4 (80.0)	2 (33.3)	4 (80.0)	13 (65.0)
Number of censored, n (%)	1 (25.0)	1 (20.0)	4 (66.7)	1 (20.0)	7 (35.0)
Time to re-emergence (days)	17.5	25.0	NE	20.0	21.0
[95% CI]	[10.0;NE]	[14.0;NE]	[NE;NE]	[10.0;NE]	[14.0;38.0]
**Time to recrudescence**					
N	4	5	6	5	20
Number of events, n (%)	3 (75.0)	3 (60.0)	2 (33.3)	4 (80.0)	12 (60.0)
Number of censored, n (%)	1 (25.0)	2 (40.0)	4 (66.7)	1 (20.0)	8 (40.0)
Time to re-emergence (days)	17.5	38.0	NE	20.0	23.0
[95% CI]	[10.0;NE]	[14.0;NE]	[NE;NE]	[10.0;NE]	[14.0;NE]
**Time to re-infection**					
N	4	5	6	5	20
Number of events, n (%)	0 (0.0)	1 (20.0)	0 (0.0)	0 (0.0)	1 (5.0)
Number of censored, n (%)	4 (100.0)	4 (80.0)	6 (100.0)	5 (100.0)	19 (95.0)
Time to re-emergence (days)	NE	NE	NE	NE	NE
[95% CI]	[NE;NE]	[NE;NE]	[NE;NE]	[NE;NE]	[NE;NE]

NE: not estimated.
All doses of artefenomel and ferroquine are expressed as adult-equivalent doses.
Note: Patients with no event were censored at the time of study completion, premature study discontinuation, including switch to established anti-malarial treatment or start of any other treatment with anti-malarial activity, whichever was earliest.
Re-emergence/Recurrence (recrudescence and re-infection): The appearance of asexual parasites after clearance of initial infection irrespective of genotype.
Recrudescence: The appearance of asexual parasites after clearance of initial infection with a genotype identical to that of parasites present at baseline. Recrudescence was confirmed by microscopy (positive blood smear) and by genotyping PCR analysis.
Re-infection: The appearance of asexual parasites after clearance of initial infection with a genotype that differed from that of parasites present at baseline. Re-infection was confirmed by microscopy (positive blood smear) and by genotyping PCR analysis. Confirmed new infection was not regarded as treatment failure or recrudescence.

Table S 10 Crude ACPR at Day 28, 42 and 63 in African patients aged >5 years and Asian patients (mITT Sets)

	Artefenomel mg: Ferroquine mg				
	800:400	800:600	800:900	800:1200	Total
**African patients >5 years**					
**Day 28**					
N	16	15	17	14	62
Crude ACPR, n/r (%)	7/16 (43.8)	12/15 (80.0)	12/17 (70.6)	12/14 (85.7)	43/62 (69.4)
[95% CI]	[19.8; 70.1]	[51.9; 95.7]	[44.0; 89.7]	[57.2; 98.2]	[56.3; 80.4]
**Day 42**					
N	16	15	17	14	62
Crude ACPR, n/r (%)	6/16 (37.5)	5/15 (33.3)	11/17 (64.7)	10/14 (71.4)	32/62 (51.6)
[95% CI]	[15.2; 64.6]	[11.8; 61.6]	[38.3; 85.8]	[41.9; 91.6]	[38.6; 64.5]
**Day 63**					
N	16	15	17	14	62
Crude ACPR, n/r (%)	2/16 (12.5)	4/15 (26.7)	7/17 (41.2)	7/14 (50.0)	20/62 (32.3)
[95% CI]	[1.6; 38.3]	[7.8; 55.1]	[18.4; 67.1]	[23.0; 77.0]	[20.9; 45.3]
**Asian patients**					
**Day 28**					
N	4	5	6	5	20
Crude ACPR, n/r (%)	1/4 (25.0)	1/5 (20.0)	2/6 (33.3)	1/5 (20.0)	5/20 (25.0)
[95% CI]	[0.6; 80.6]	[0.5; 71.6]	[4.3; 77.7]	[0.5; 71.6]	[8.7; 49.1]
**Day 42**					
N	4	5	6	5	20
Crude ACPR, n/r (%)	1/4 (25.0)	1/5 (20.0)	2/6 (33.3)	1/5 (20.0)	5/20 (25.0)
[95% CI]	[0.6; 80.6]	[0.5; 71.6]	[4.3; 77.7]	[0.5; 71.6]	[8.7; 49.1]
**Day 63**					
N	4	5	6	5	20
Crude ACPR, n/r (%)	0/4 (0)	1/5 (20.0)	2/6 (33.3)	1/5 (20.0)	4/20 (20.0)
[95% CI]	[-; -]	[0.5; 71.6]	[4.3; 77.7]	[0.5; 71.6]	[5.7; 43.7]

All doses of artefenomel and ferroquine are expressed as adult-equivalent doses.
N: total number of patients in the relevant analysis set.
n: number of patients in each category achieving ACPR.
r: total number of patients in the relevant analysis set with a defined response of Cure or Failure (i.e. patients evaluable for the outcome considered).

Parasite clearance kinetics

Table S 11 Parasite clearance time (Kaplan-Meier estimates) by region (PP Sets)

	**Artefenomel mg: Ferroquine mg**				
	**800:400**	**800:600**	**800:900**	**800:1200**	**Total**
**African patients >6 months and ≤5 years (PP Set)**					
N	69	73	70	69	281
PCT (h), KM estimate [95% CI]	36.0 (24.23; 36.07)	36.0 (35.93; 36.10)	36.1 (36.00; 48.00)	36.1 (24.25; 36.42)	36.0 (36.00; 36.10)
Patients achieving clearance, estimated rate, % [95% CI]					
At 24h	24.6 (16.10; 36.60)	24.0 (15.48; 36.22)	17.7 (10.24; 29.74)	21.0 (12.76; 33.35)	22.0 (17.41; 27.52)
At 48h	73.9 (63.21; 83.57)	74.2 (63.24; 83.98)	64.8 (52.94; 76.41)	64.7 (52.90; 76.35)	69.6 (63.91; 75.09)
At 72h	79.7 (68.18; 89.16)	77.4 (66.70; 86.62)	79.0 (64.50; 90.52)	64.7 (52.90; 76.35)	76.7 (70.10; 82.83)
**African patients >5 years (African >5 years PP Set)**					
N	11	11	12	10	44
PCT (h), KM estimate [95% CI]	24.0 (18.00; 24.18)	24.3 (18.07; 36.00)	24.3 (12.23; 36.00)	27.2 (6.15; 36.17)	24.2 (18.15; 24.58)
Patients achieving clearance, estimated rate, % [95% CI]					
At 24h	54.5 (29.31; 83.34)	45.5 (22.04; 77.15)	25.0 (8.83; 59.16)	50.0 (24.68; 81.64)	43.2 (30.12; 59.00)
At 48h	100 (100; 100)	90.9 (66.71; 99.46)	91.7 (68.89; 99.49)	80.0 (52.53; 96.91)	90.9 (80.29; 97.10)
At 72h	100 (100; 100)	NE	NE	80.0 (52.53; 96.91)	90.9 (80.29; 97.10)
**Asian patients (Asian PP Set)**					
N	4	5	5	5	19
PCT (h), KM estimate [95% CI]	82.0 (79.97; NE)	75.1 (53.93; 95.82)	79.8 (36.00; NE)	72.2 (60.08; 79.98)	79.8 (72.00; 80.07)
Patients achieving clearance, estimated rate, % [95% CI]					
At 24h	NE	NE	NE	NE	NE
At 48h	NE	NE	40.0 (11.82; 87.43)	NE	10.5 (2.74; 35.92)
At 72h	NE	25.0 (3.95; 87.21)	40.0 (11.82; 87.43)	40.0 (11.82; 87.43)	27.3 (12.36; 53.73)

KM = Kaplan-Meier; NE = not estimated.
All doses of artefenomel and ferroquine are expressed as adult-equivalent doses.
N: total number of patients in the relevant analysis set.

Patients with no event were censored at the time of study completion, premature study discontinuation, including switch to established anti-malarial treatment or start of any other treatment with anti-malarial activity, whichever was earliest.

Table S 12 Parasite clearance parameters by region (PP Sets)

	Artefenomel mg: Ferroquine mg				
	**800:400**	**800:600**	**800:900**	**800:1200**	**Total**
**African patients >6 months and ≤5 years (PP Set)**					
Number in Set	69	67	63	62	261
Number analysed	63	57	58	53	231
Clearance rate constant (/h)	0.28 (0.11; 0.51)	0.29 (0.11; 0.54)	0.23 (0.12; 0.46)	0.25 (0.15; 0.50)	0.27 (0.11; 0.54)
PCt1/2 (h), median (range)	2.52 (1.35; 6.06)	2.40 (1.28; 6.56)	3.01 (1.52; 5.78)	2.79 (1.38; 4.76)	2.57 (1.28; 6.56)
PRR24 (log10), median (range)	2.87 (1.19; 5.35)	3.01 (1.10; 5.63)	2.40 (1.25; 4.75)	2.59 (1.52; 5.24)	2.82 (1.10; 5.63)
PRR48 (log10), median (range)	5.73 (2.39; 10.69)	6.02 (2.20; 11.27)	4.79 (2.50; 9.50)	5.17 (3.04; 10.48)	5.63 (2.20; 11.27)
Time to 50% parasite reduction (h), median (range)[a]	4.67 (0.04; 17.18)	4.43 (0.34; 16.36)	4.85 (1.23; 16.91)	4.85 (0.04; 34.85)	4.68 (0.04; 34.85)
Time to 90% parasite reduction (h), median (range)	9.94 (4.89; 31.24)	10.37 (5.03; 25.01)	11.81 (3.05; 29.67)	10.91 (4.99; 45.37)	10.80 (3.05; 45.37)
Time to 99% parasite reduction (h), median (range)	18.15 (10.64; 51.36)	18.56 (10.89; 44.21)	22.04 (9.72; 48.88)	20.79 (9.57; 60.44)	19.78 (9.57; 60.44)
**African patients >5 years (African >5 years PP Set)**					
Number in Set	11	11	12	10	44
Number analysed	7	9	10	8	34
Clearance rate constant (/h)	0.29 (0.23; 0.41)	0.39 (0.21; 0.48)	0.32 (0.22; 0.46)	0.29 (0.17; 0.54)	0.32 (0.17; 0.54)
PCt1/2 (h), median (range)	2.43 (1.68; 2.99)	1.78 (1.44; 3.34)	2.20 (1.50; 3.14)	2.37 (1.29; 4.10)	2.20 (1.29; 4.10)
PRR24 (log10), median (range)	2.98 (2.41; 4.30)	4.07 (2.16; 5.00)	3.29 (2.30; 4.81)	3.05 (1.76; 5.59)	3.29 (1.76; 5.59)
PRR48 (log10), median (range)	5.96 (4.83; 8.60)	8.14 (4.32; 10.00)	6.58 (4.61; 9.63)	6.11 (3.52; 11.17)	6.58 (3.52; 11.17)
Time to 50% parasite reduction (h), median (range)[a]	4.59 (0.73; 9.38)	6.01 (1.46; 9.94)	4.61 (0.75; 10.69)	2.70 (0.40; 4.62)	4.34 (0.40; 10.69)

	Artefenomel mg: Ferroquine mg				
	800:400	800:600	800:900	800:1200	Total
Time to 90% parasite reduction (h), median (range)	11.14 (4.63; 14.49)	11.96 (5.20; 15.11)	9.76 (4.24; 17.97)	7.76 (3.84; 12.58)	9.48 (3.84; 17.97)
Time to 99% parasite reduction (h), median (range)	20.53 (10.21; 23.99)	17.52 (10.56; 26.22)	17.71 (9.23; 28.39)	15.27 (8.14; 23.97)	17.11 (8.14; 28.39)
**Asian patients (Asian PP Set)**					
Number in Set	4	5	5	5	19
Number analysed	4	5	4	4	17
Clearance rate constant (/h)	0.11 (0.10; 0.12)	0.11 (0.10; 0.14)	0.16 (0.10; 0.30)	0.13 (0.11; 0.16)	0.12 (0.10; 0.30)
PCt1/2 (h), median (range)	6.22 (5.66; 7.13)	6.42 (5.02; 7.25)	4.89 (2.32; 6.74)	5.36 (4.37; 6.55)	6.00 (2.32; 7.25)
PRR24 (log10), median (range)	1.17 (1.01; 1.28)	1.13 (1.00; 1.44)	1.69 (1.07; 3.12)	1.35 (1.10; 1.65)	1.20 (1.00; 3.12)
PRR48 (log10), median (range)	2.34 (2.03; 2.56)	2.25 (1.99; 2.88)	3.38 (2.14; 6.24)	2.71 (2.21; 3.31)	2.41 (1.99; 6.24)
Time to 50% parasite reduction (h), median (range)[a]	8.21 (5.09; 15.67)	8.11 (3.20; 22.62)	7.55 (2.41; 20.39)	4.46 (3.59; 7.83)	7.49 (2.41; 22.62)
Time to 90% parasite reduction (h), median (range)	23.06 (18.48; 31.18)	24.10 (17.13; 37.53)	19.05 (7.80; 35.76)	17.39 (14.87; 20.93)	19.98 (7.80; 37.53)
Time to 99% parasite reduction (h), median (range)	44.30 (37.63; 53.37)	46.97 (35.81; 58.87)	35.49 (15.49; 57.75)	35.94 (29.39; 41.19)	39.67 (15.49; 58.87)

a. Number of evaluable patients for Time to 50% parasite reduction: 227 African patients >6 months and ≤5 years, 33 African patients >5 years and 17 Asian patients.
Parasite clearance parameters were estimated using the WWARN Parasite Clearance Estimator based on the linear part of the individual natural log parasitaemia-time profiles. Data from patients with a poor fit to the linear model (r^2 <0.75) or with <3 data points were excluded from the analysis.
Observations taken from patients who received rescue medication, or from patients with <3 observations within the first 24 h were not included in the WWARN Parasite Clearance Estimator. Only data up to 168 h after study drug administration were included in the analysis.
All doses of artefenomel and ferroquine are expressed as adult-equivalent doses.

Fever clearance time (FCT)

Time to fever clearance was estimated in the few patients in the PP Set who had documented fever at baseline and did not receive paracetamol. Across all ferroquine doses, FCT ranged from 1.0 to 24.0 h (median 1.0 h) in African patients ≤5 years (n=16), from 1.0 to 18.0 h

(median 1 h) in African patients >5 years (n=7), and from 18.0 to 36.0 h in Asian patients (n=2).

Time to gametocyte appearance/clearance

Gametocytes were differentiated from asexual forms by measuring messenger ribonucleic acid (mRNA) transcripts of gametocyte-specifically expressed genes (Pfs25) using reverse transcriptase PCR, which allowed the detection of sub-microscopic gametocytemia and characterization of gametocyte maturation stage.

The majority of patients in the mITT Sets had no gametocytes detectable at baseline (254/281 African patients ≤5 years, 61/62 African patients >5 years, and 16/20 Asian patients). In patients with gametocytes at baseline, time to gametocyte clearance was not estimatable. In patients without gametocytes at baseline, time to gametocyte appearance was not estimatable for most ferroquine doses.

Safety and tolerability

Table S 13 Overview of adverse event profile: Treatment-emergent adverse events (Safety Set)

n (%) patients with any TEAE	Artefenomel mg: Ferroquine mg				
	800:400	800:600	800:900	800:1200	Total
N	92	94	96	91	373
Any TEAE	84 (91.3)	87 (92.6)	85 (88.5)	81 (89.0)	337 (90.3)
Any severe TEAE	0	3 (3.2)	3 (3.1)	2 (2.2)	8 (2.1)
Any ferroquine-related TEAE	28 (30.4)	26 (27.7)	38 (39.6)	35 (38.5)	127 (34.0)
Any artefenomel-related TEAE	29 (31.5)	26 (27.7)	34 (35.4)	35 (38.5)	124 (33.2)
Any TEAE leading to permanent treatment discontinuation[a]	3 (3.3)	2 (2.1)	3 (3.1)	2 (2.2)	10 (2.7)
Any treatment-emergent SAE	0	2 (2.1)	4 (4.2)	2 (2.2)	8 (2.1)
Any TEAE leading to death	0	0	0	0	0
Any treatment-emergent AESI[b]	5 (5.4)	8 (8.5)	9 (9.4)	11 (12.1)	33 (8.8)

Data presented are the number (%) of patients with at least 1 TEAE in the Safety Set.
TEAEs were AEs that developed or worsened or became serious during the on-treatment phase, i.e. time from the start of the first dose of study drug administration (included) up to the Day 63 visit (included). All doses of artefenomel and ferroquine are expressed as adult-equivalent doses.

a. Permanent treatment discontinuation was the withdrawal of at least 1 of the study drugs, either ferroquine or artefenomel.
b. The following treatment-emergent AESIs were defined in the protocol: increase in ALT (ALT ≥3x ULN if baseline ALT <ULN, or ALT ≥2x baseline value if baseline ALT ≥ULN); QTcF ≥500 ms or QTcF prolongation >60 ms from baseline; pregnancy and follow-up; symptomatic overdose with the study drug.

Table S 14 Summary of treatment-emergent adverse events reported in ≥5% of the African patients aged ≤5 years in any treatment arm (Safety Set, African patients ≤5 years)

**Preferred Term**	**Artefenomel mg: Ferroquine mg**				
	**800:400**	**800:600**	**800:900**	**800:1200**	**Total**
N	72	74	72	71	289
**At least 1 TEAE**	66 (91.7)	67 (90.5)	64 (88.9)	63 (88.7)	260 (90.0)
Malaria	44 (61.1)	38 (51.4)	30 (41.7)	33 (46.5)	145 (50.2)
Vomiting	22 (30.6)	24 (32.4)	26 (36.1)	31 (43.7)	103 (35.6)
Cough	9 (12.5)	7 (9.5)	12 (16.7)	14 (19.7)	42 (14.5)
Upper Respiratory Tract Infection	7 (9.7)	6 (8.1)	17 (23.6)	10 (14.1)	40 (13.8)
Diarrhoea	8 (11.1)	8 (10.8)	7 (9.7)	8 (11.3)	31 (10.7)
Pyrexia	5 (6.9)	2 (2.7)	8 (11.1)	9 (12.7)	24 (8.3)
Electrocardiogram QT Prolonged	5 (6.9)	5 (6.8)	5 (6.9)	7 (9.9)	22 (7.6)
Decreased Appetite	3 (4.2)	5 (6.8)	4 (5.6)	3 (4.2)	15 (5.2)
Bronchitis	3 (4.2)	5 (6.8)	3 (4.2)	3 (4.2)	14 (4.8)
Abdominal Pain	4 (5.6)	3 (4.1)	4 (5.6)	2 (2.8)	13 (4.5)
Rhinitis	3 (4.2)	3 (4.1)	1 (1.4)	6 (8.5)	13 (4.5)
Anaemia	3 (4.2)	4 (5.4)	1 (1.4)	2 (2.8)	10 (3.5)
Gastroenteritis	3 (4.2)	2 (2.7)	1 (1.4)	4 (5.6)	10 (3.5)
Respiratory Tract Infection	4 (5.6)	1 (1.4)	1 (1.4)	1 (1.4)	7 (2.4)

MedDRA version 22.1. All doses of artefenomel and ferroquine are expressed as adult-equivalent doses.
Data presented are the number (%) of patients with at least 1 TEAE in the Safety Set, African patients ≤5 years.
TEAEs were AEs that developed or worsened or became serious during the on-treatment phase, i.e. time from the start of the first dose of study drug administration (included) up to the Day 63 visit (included).
Table sorted by decreasing frequency of preferred term pooled treatment arms.

References

WorldWide Antimalarial Resistance Network (WWARN). Parasite Clearance Estimator.

https://www.wwarn.org/parasite-clearance-estimator-pce. Accessed 15 December 2020
