# Supplementary material for: A randomized, double-blind, phase 2b study to investigate the efficacy, safety, tolerability and pharmacokinetics of a single-dose regimen of ferroquine with artefenomel in adults and children with uncomplicated Plasmodium falciparum malaria
Source: Malar J. 2021 May 19;20:222. doi: 10.1186/s12936-021-03749-4 (PMC8135182; doi:10.1186/s12936-021-03749-4)
Supplement: Supplementary file 3 — Additional file 3. Pharmacokinetic analysis details. Supplementary document including tables and figures to provide further methodological details and results on the pharmacokinetic analysis. [file 12936_2021_3749_MOESM3_ESM.pdf]

S3 Pharmacokinetic Analysis Details

Note. Some of the figures and tables refer to "OZ" or "OZ439" which are the same as artefenomel.

No formal population PK analysis of either artefenomel or ferroquine has been performed on the study data, contrary to what was specified in the protocol. Instead, population PK techniques were applied using historical population PK models to estimate only the individual patient PK parameters. The latter approach was pre-specified in an analysis plan.

All data processing, analysis, model setup and modelling result analysis were conducted within R (Microsoft Open R 3.5.1) combined with the IQR package (v1.1.1) developed by IntiQuan (IQR Tools, https://iqrtools.intiquan.com) to support the entire workflow of a PK/PD analysis from estimations to simulations. For all estimations, a nonlinear mixed effects (NLME) modelling approach was performed using the stochastic approximation expectation maximization (SAEM) method of Monolix (Monolix version 2019R1. Antony, France: Lixoft SAS, 2019) by automatically generating and running MONOLIX projects through IQR from R environment. The log-likelihood and the Fisher information matrix were approximated by linearization. The individual parameters were determined as conditional modes.

Pharmacokinetic Samples

Per protocol, samples for pharmacokinetic analysis of artefenomel in plasma and ferroquine (FQ)/SSR97213 in blood (dried blood spot) were collected at a total of 16 time points for each patient in patients >14 years and body weight ≥35 kg (11 for artefenomel and 13 for FQ/SSR97213). In the younger patients the number of samples collected was either 5 for artefenomel or 7 for FQ/SSR97231 for patients >5 to ≤14 years or 4/5 for artefenomel and 5 for FQ/SSR97213 for patients >6 months to ≤5 years.

The samples were analysed for artefenomel, ferroquine and SSR97213 by LC MS/MS. The limit of quantification was 5 ng/mL for FQ/SSR97213 and 1 ng/mL for artefenomel. Using human blood, an LC-MS /MS assay method for quantification of FQ and its metabolite SSR97213 in dried blood spot (DBS) over a range of 5-500 ng/mL was developed and validated. Method was specific and selective relative to endogenous compounds, with process efficiency, 60%, and no matrix effect. Accuracy and precision for intraday and interday analyzes were <15% at all concentrations tested. FQ and its metabolite SSR97213 were stable at room temperature for a duration to cover study samples, and for at least 24 hours at 37°C with and without 95% relative humidity, to cover sampling, drying, and shipment conditions in the field.

Observations below the quantification limit (BQL) were included in the data set. Monolix handles these (left-censored data) by including the simulation of the censored data with a truncated Gaussian distribution in the Markov Chain Monte Carlo (MCMC) procedure.

Pharmacokinetic Analysis

The analysis used a non-linear mixed effect modelling approach as implemented in Monolix. The individual Empirical Bayes Estimates of the PK parameters for each individual patient and each analyte (artefenomel, FQ and SSR97213) were estimated applying the historical population PK models developed previously (described below). The historical structural population PK model was fitted to the observed data and dosing history of the study, providing the individual PK parameters for each subject as post-hoc estimates. From these, the individual exposures were estimated through simulation and calculation: C_{max}, t_{max}, C_{day7}, C_{day14}, C_{day21}, C_{day28}, $AUC_{(0-\infty)}$ as well as $AUC_{(0-day28)}$ (for FQ and SSR97213 only).

All patients who vomited after either ferroquine (which was administered first) or artefenomel were considered vomiters for the analyses and summaries, unless they were successfully re-dosed. If patients vomited after ferroquine, they were not to be re-dosed with ferroquine but received rescue medication. If patients vomited within 5 minutes after artefenomel administration, they were to be re-dosed with artefenomel only. These patients were not considered vomiters for the artefenomel analysis, but they were considered vomiters for the ferroquine summaries (this concerned 4 patients in the PK Set for ferroquine).

Historical Population PK models

A single population PK model including both ferroquine and its active metabolite SSR97213 was previously developed using data of 541 subjects from eight phase I and II studies conducted in healthy volunteers (HV), asymptomatic adult subjects and symptomatic adult and pediatric patients (>2 years old) [Boulu 2016]. It included a three- and two-compartment PK model for FQ and SSR97213 respectively. The absorption of FQ was described with a first-order absorption process, lag-time and relative bio-availability. Allometric scaling was implemented and disease status (*i.e* HV and asymptomatic subject *versus* symptomatic patients) was identified as a covariate. Some data (African adults >35 kg) from study DRI12805, where FQ was co-administered with artefenomel, were included in the model development. No effect of artefenomel on the pharmacokinetics of FQ or its metabolite was identified (Table 1).

Table 1 Parameter estimates ferroquine historical population PK model in Patients [Boulu 2016]

Parameter		Estimate[a]	BSV[b] (%)
**ferroquine**			
F	Relative Oral Bioavailability	1	-
tlag (hr)	Absorption lag time	0.45 (1)	-
ka (1/hr)	Absorption rate constant	0.71 (8)	110 (12)
Cl/F (L/hr)	Apparent Clearance	$13.5(6) * \left(\frac{BW}{65}\right)^{0.75}$	46 (10)
V1/F (L)	Apparent central volume of distribution	$2590(4) * \left(\frac{BW}{65}\right)^{1}$	56 (10)
Q/F (L/hr)	Apparent inter compartmental Clearance 1	$141(7) * \left(\frac{BW}{65}\right)^{0.75}$	90 (13)
V2/F (L)	Apparent peripheral volume of distribution 1	$3540(4) * \left(\frac{BW}{65}\right)^{1}$	37 (16)
Q2/F (L/hr)	Apparent inter compartmental Clearance 2	$19.5(8) * \left(\frac{BW}{65}\right)^{0.75}$	98 (11)
V3/F (L)	Apparent peripheral volume of distribution 2	12400 (5)	-
**SSR97213**			
Clpm (L/hr)	Transformation FQ to SSR	0.25 (4)	19 (18)
CL/F (L/hr)	Apparent Clearance	0.20 (4)	16 (26)
V1/F (L)	Apparent central volume of distribution	1	-
Q/F (L/hr)	Apparent inter compartmental Clearance 1	0.72 (6)	42 (12)
V2/F (L)	Apparent peripheral volume of distribution 1	43 (5)	47 (18)
residual	Proportional ferroquine	0.22 (2)	
residual	Proportional SSR97213	0.26 (2)	-

[a]Estimate, with between brackets the RSE (Relative Standard Error %)
[b]Between Subject Variability
BW= body weight (kg)

For artefenomel a population PK model was previously developed based on three phase II studies: two mono therapy studies (MMV_OZ439_10_002 and MMV_OZ439_12_006) and one study in combination with piperaquine which included African and Asian men and women; age 6 months to 60 years; body weight range 5.6 - 89 kg; single doses 100 - 1200 mg; 800 mg when dosed with piperaquine (MMV_OZ439_13_003) [Macintyre 2017]. It included a three-compartment disposition model with first-order absorption and a lag-time. Body weight was included allometrically using fixed exponents for of 0.75 for clearances and 1 for volumes. Vomiting, age, actual artefenomel dose and adult equivalent artefenomel dose were all identified as covariates. In none of the studies, artefenomel was co-administered with FQ (Table 2).

Table 2 Parameter estimates artefenomel historical population PK model in Patients [Macintyre 2017]

Parameter		Estimate[a]	BSV[b] (%)
F	Relative Oral Bioavailability	$1 * \left(\frac{AGE}{20}\right)^{0.19(12)}$	62 (4)
Fvom	Relative Oral Bioavailability in Vomiters	$0.51(9) * \left(\frac{AGE}{20}\right)^{0.19(12)}$	86 (8)
tlag (hr)	Absorption lag time	0.41 (1)	14 (8)
ka (1/hr)	Absorption rate constant	$0.17(2) * \left(\frac{ODOS}{800}\right)^{-0.34(8)}$	22 (8)
Cl/F (L/hr)	Apparent Clearance	$49.2(2) * \left(\frac{BW}{50}\right)^{0.75} \left(\frac{ODGP}{800}\right)^{-0.37(11)}$	33 (5)
V1/F (L)	Apparent central volume of distribution	$135(5) * \left(\frac{BW}{50}\right)^{1}$	73 (6)
Q/F (L/hr)	Apparent inter compartmental Clearance 1	$9.7(4) * \left(\frac{BW}{50}\right)^{0.75}$	36 (8)
V2/F (L)	Apparent peripheral volume of distribution 1	$269(5) * \left(\frac{BW}{50}\right)^{1}$	-
Q2/F (L/hr)	Apparent inter compartmental Clearance 2	$7.0(3) * \left(\frac{BW}{50}\right)^{0.75}$	-
V3/F (L)	Apparent peripheral volume of distribution 2	$1130(4) * \left(\frac{BW}{50}\right)^{1}$	51 (2)
residual	proportional	0.26 (2)	-

[a]Estimate, with between brackets the RSE (Relative Standard Error %)
[b]Between Subject Variability
AGE=age (years); ODOS=actual administered dose (mg);ODGP=adult equivalent dose (mg); BW= body weight (kg)

RESULTS

Data

The analysis population (PK set) for artefenomel consisted of 364 patients out of 366 patients who were dosed with artefenomel. The PK Population for ferroquine consisted of 367 patients out of 373 patients who were dosed with ferroquine. The data sets included a total of 2220 samples for artefenomel and 2345/2348 samples for ferroquine/SSR97213.
There were no missing covariates or sample date-times for any of the three analytes. For the artefenomel analysis 1 sample was removed from the analysis because of a positive pre-dose artefenomel concentration. For the ferroquine/SSR97213 analysis 1 patient was removed since all samples were BLQ. This patient had vomited after FQ administration, and had received rescue medication as per protocol.

Estimation of PK parameters
The comparison of observed artefenomel and ferroquine concentrations with the historical PK model predictions for this study population over the first 200 or 400 hours post-dose are shown in figures 1 and 2 respectively.
The historical population PK models were used to estimate the individual patient PK parameters, with all model parameter values fixed except for the residual proportional error. The estimated residual proportional errors were similar to the historical model values (0.27 and 0.26, respectively for artefenomel and 0.30/0.31 and 0.22/0.26 respectively for FQ/SSR97213).
The individual patient PK profiles were well described by the estimated individual PK parameters and considered adequate. Examples are shown below in Figures 3 and 5. More specifically, the observed concentrations around day 7, when available, were well described (Figures 4 and 6).

Figure 1 Comparison of Observed Artefenomel Concentrations with the Historical Population PK Model Predictions by Region-Age Group

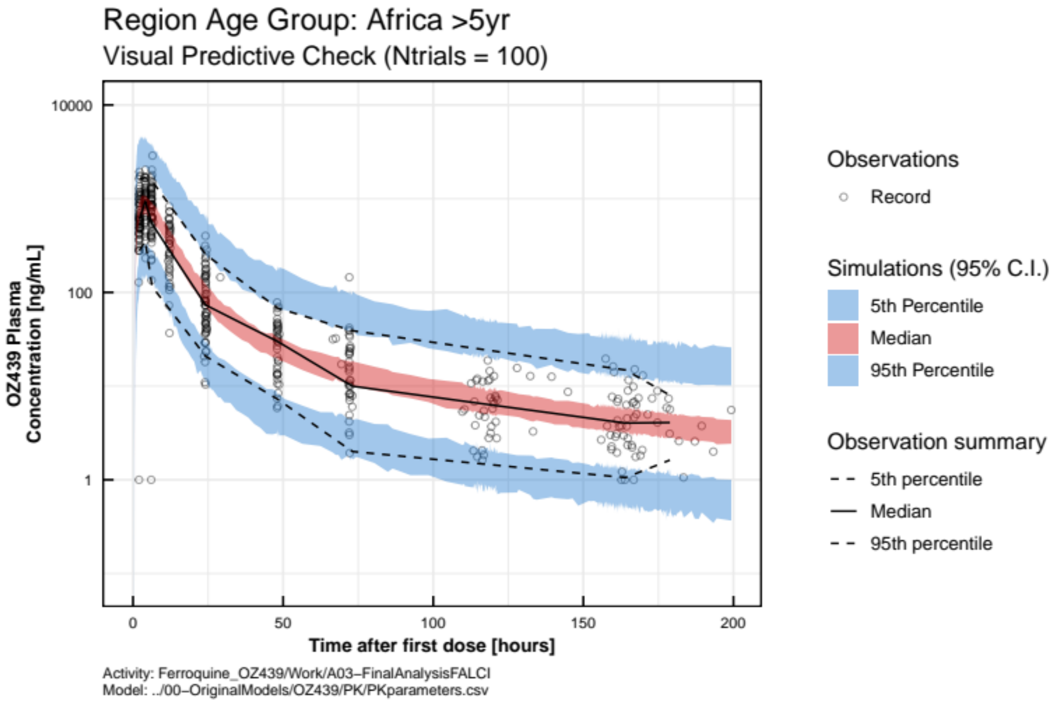

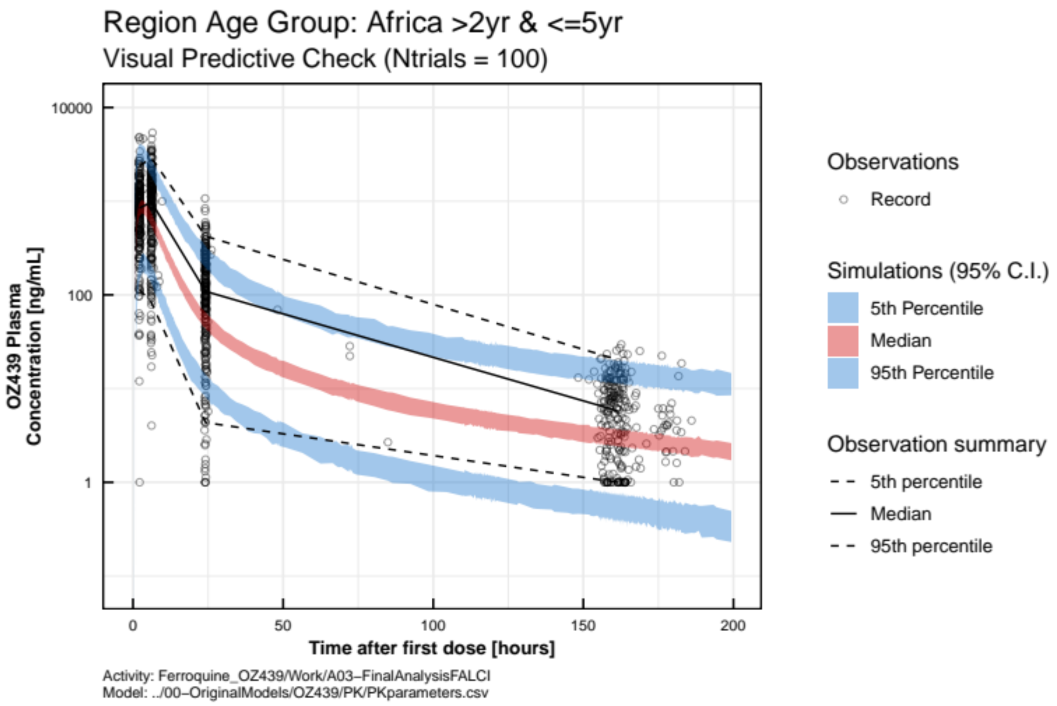

Region Age Group: Africa >2yr & <=5yr
Visual Predictive Check (Ntrials = 100)
10000
100
1
0
50
100
150
200
OZ439 Plasma Concentration [ng/mL]
Time after first dose [hours]
Observations
Record
Simulations (95% C.I.)
5th Percentile
Median
95th Percentile
Observation summary
5th percentile
Median
95th percentile
Activity: Ferroquine_OZ439/Work/A03–FinalAnalysisFALCI
Model: ../00–OriginalModels/OZ439/PK/PKparameters.csv

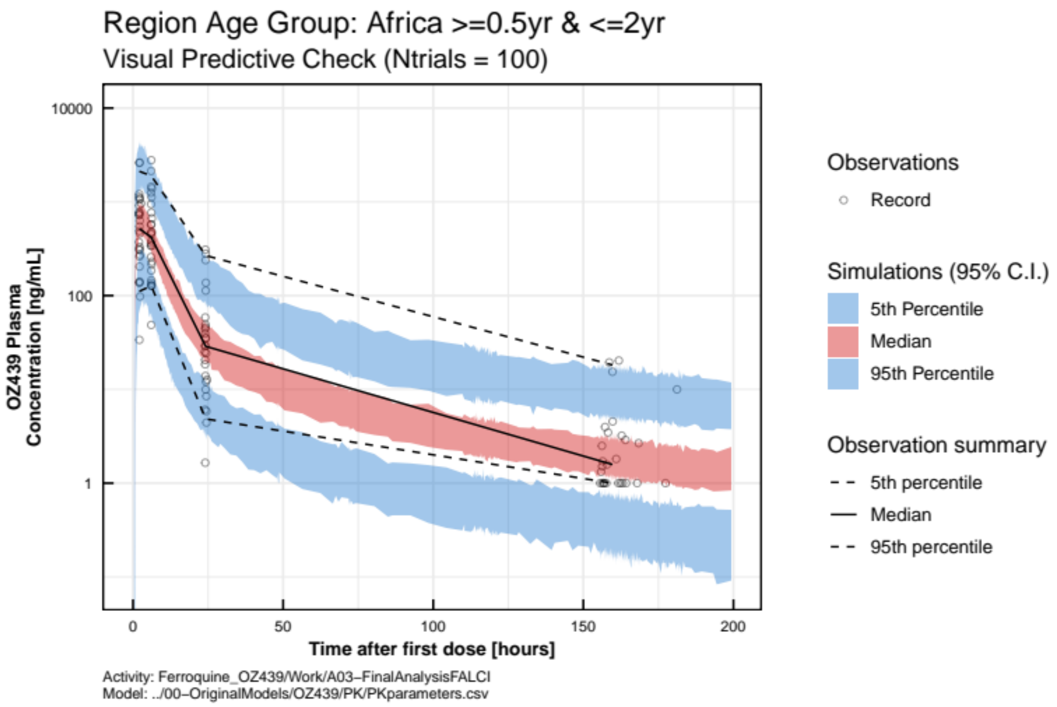

Region Age Group: Africa >=0.5yr & <=2yr
Visual Predictive Check (Ntrials = 100)
10000
100
1
0
50
100
150
200
OZ439 Plasma Concentration [ng/mL]
Time after first dose [hours]
Observations
Record
Simulations (95% C.I.)
5th Percentile
Median
95th Percentile
Observation summary
5th percentile
Median
95th percentile
Activity: Ferroquine_OZ439/Work/A03–FinalAnalysisFALCI
Model: ../00–OriginalModels/OZ439/PK/PKparameters.csv

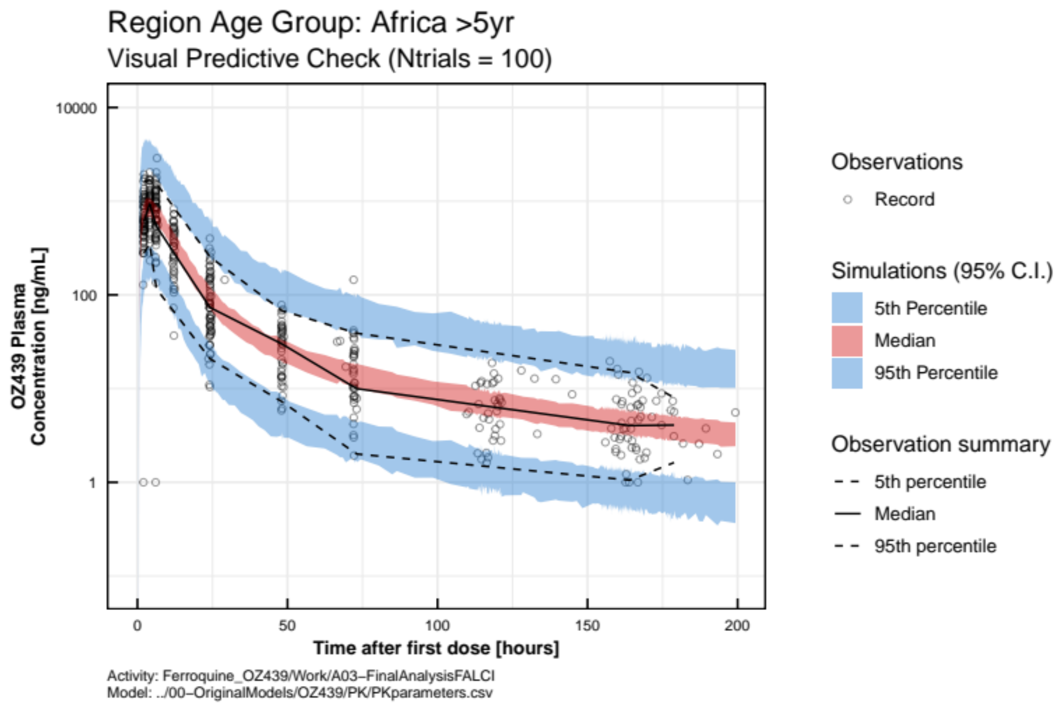

Figure 2 Comparison of Observed Ferroquine Concentrations with the Historical Population PK Model Predictions by Region-Age Group

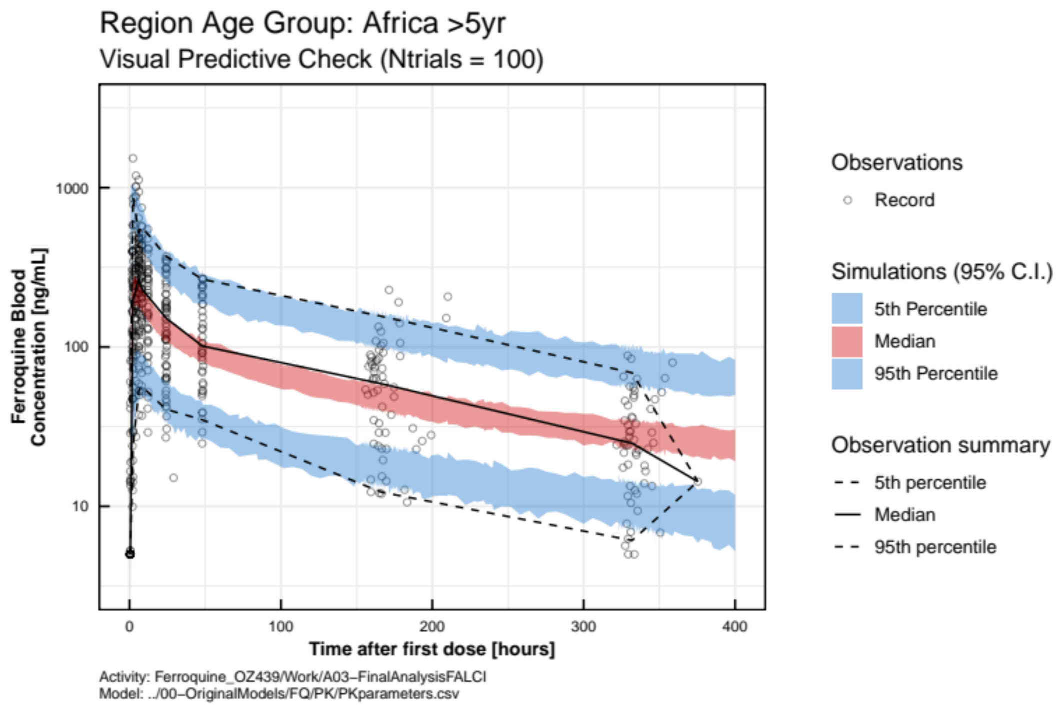

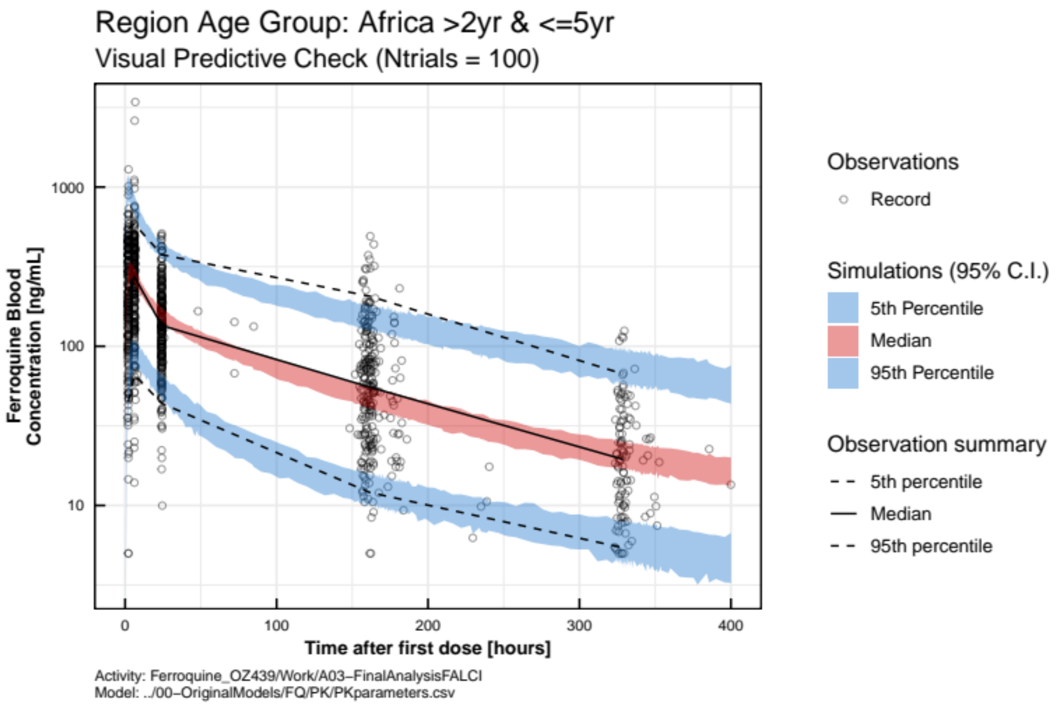
Region Age Group: Africa >2yr & <=5yr
Visual Predictive Check (Ntrials = 100)
Ferroquine Blood Concentration [ng/mL]
1000
100
10
0
100
200
300
400
Time after first dose [hours]
Observations
Record
Simulations (95% C.I.)
5th Percentile
Median
95th Percentile
Observation summary
5th percentile
Median
95th percentile
Activity: Ferroquine_OZ439/Work/A03–FinalAnalysisFALCI
Model: ../00–OriginalModels/FQ/PK/PKparameters.csv

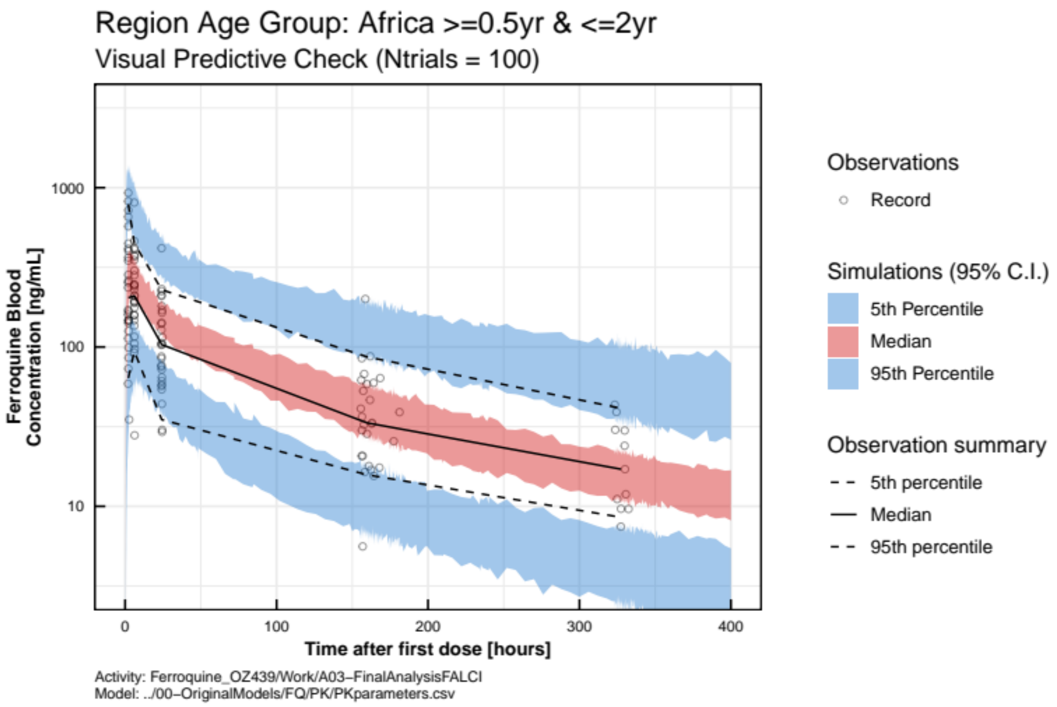
Region Age Group: Africa >=0.5yr & <=2yr
Visual Predictive Check (Ntrials = 100)
Ferroquine Blood Concentration [ng/mL]
1000
100
10
0
100
200
300
400
Time after first dose [hours]
Observations
Record
Simulations (95% C.I.)
5th Percentile
Median
95th Percentile
Observation summary
5th percentile
Median
95th percentile
Activity: Ferroquine_OZ439/Work/A03–FinalAnalysisFALCI
Model: ../00–OriginalModels/FQ/PK/PKparameters.csv

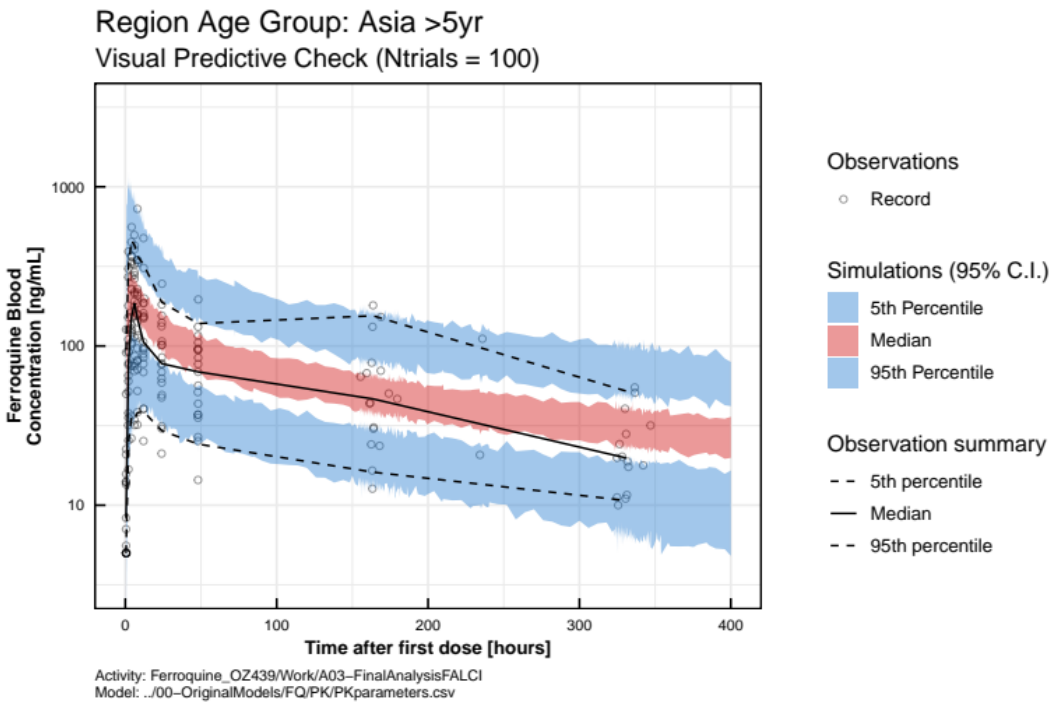

Figure 3 Example: Comparison of Observed Artefenomel Concentrations with the Individual Model Fit by Individual Patient

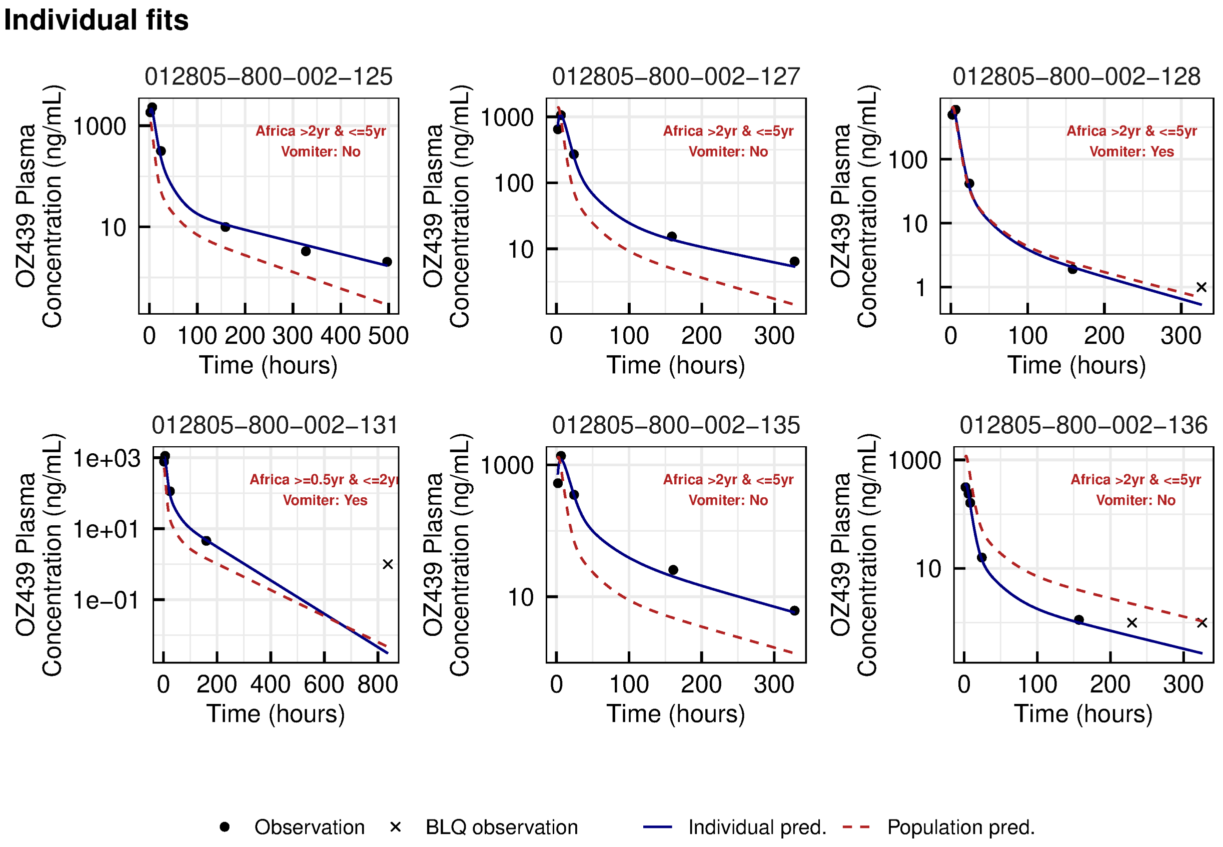

Figure 4 Observed vs Predicted Artefenomel concentrations for PK Samples taken between days 6 and 8 post-dose

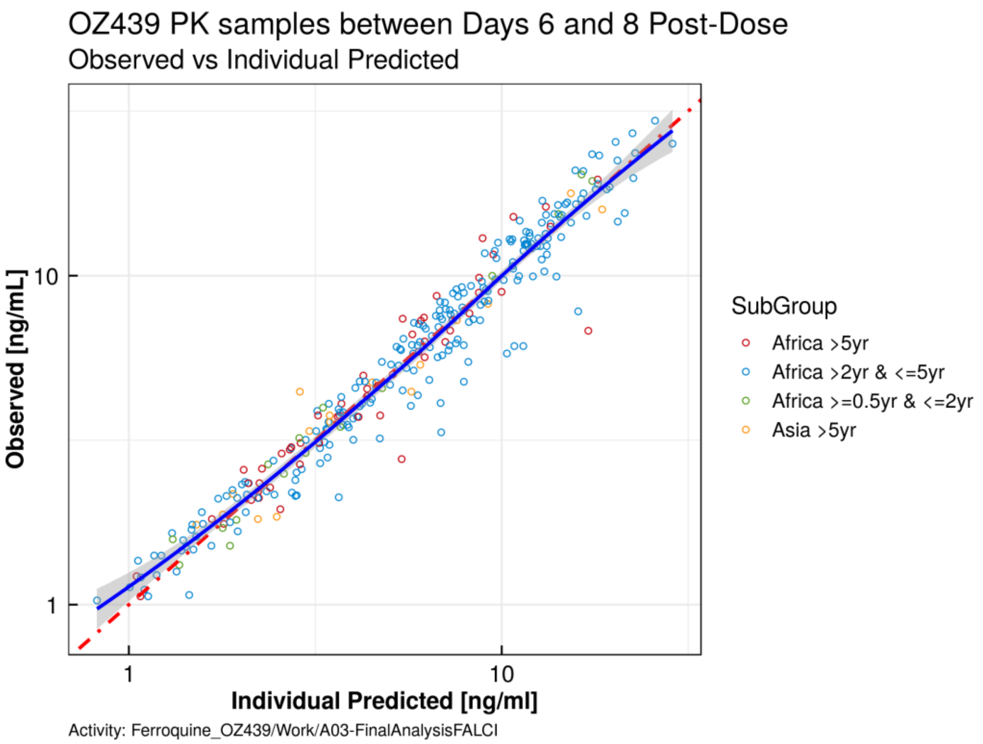

Figure 5 Example: Comparison of Observed Ferroquine Concentrations with the Individual Model Fit by Individual Patient

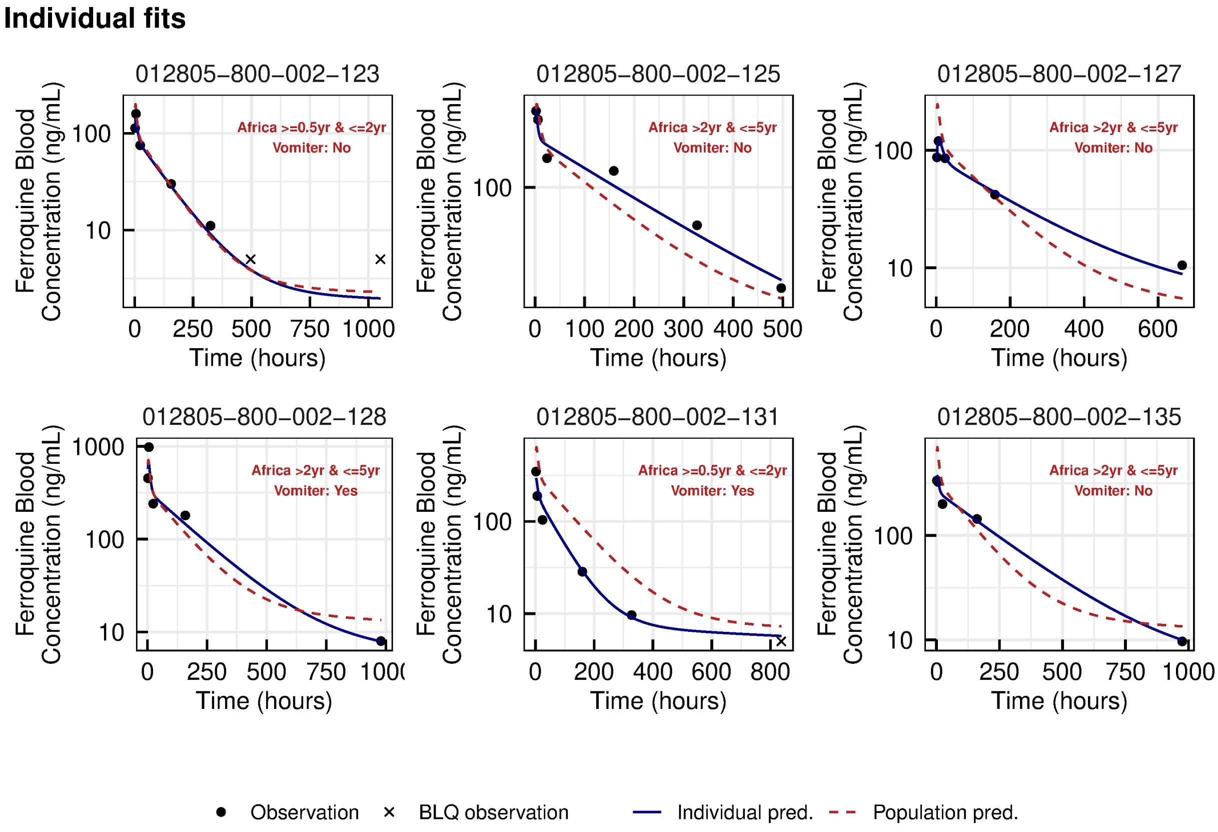

Figure 6 Observed vs Predicted Ferroquine Concentrations for PK Samples taken between days 6 and 8 post-dose

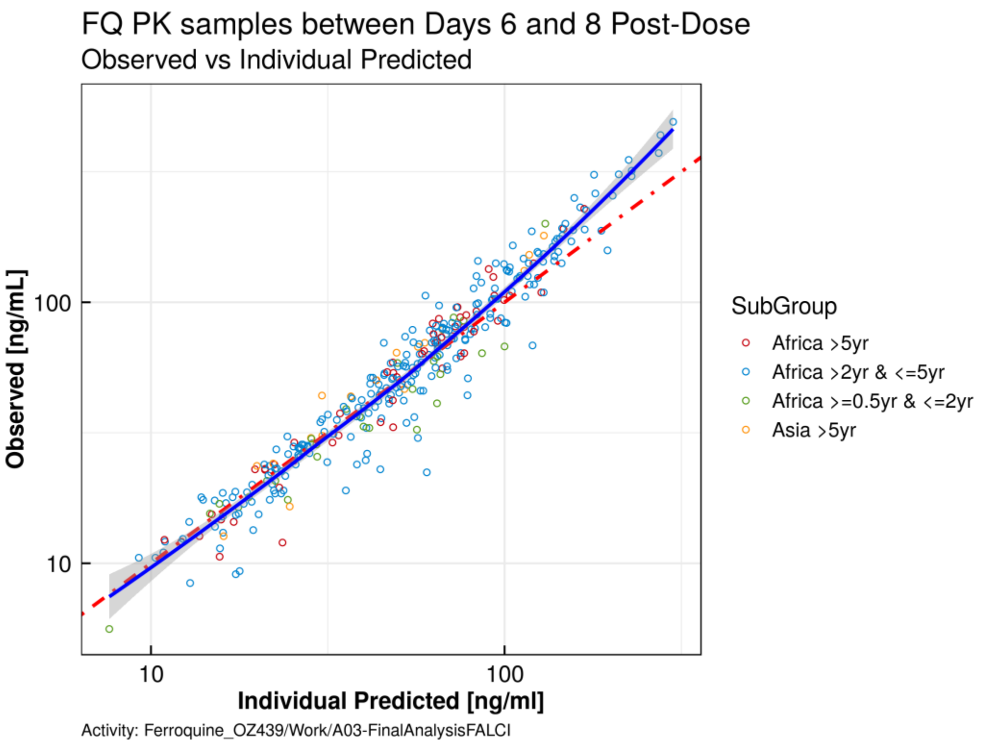

Estimated Individual Exposures

Tables 3, 4 and 5 summarize the estimated individual exposures for artefenomel, ferroquine and SSR97213, respectively.

Table 3 Selected Summary Statistics of the Individual Artefenomel Plasma Exposure Estimates by Treatment Arm for Various Sub-Populations (total n=364).

Treatment Arm	Sub-Group	n[a]	C_{max}[b] [ng/mL]	C_{day7}[b] [ng/mL]	$AUC_{(0-\infty)}$[b] [ug*hr/mL]
400mgFQ+800mgOZ	All patients	88	1072 (95%)	4.642 (170%)	14.24 (124%)
600mgFQ+800mgOZ	All patients	93	936.7 (91%)	4.212 (176%)	12.41 (127%)
900mgFQ+800mgOZ	All patients	93	771.8 (92%)	3.047 (172%)	9.621 (130%)
1200mgFQ+800mgOZ	All patients	90	797.8 (119%)	2.984 (196%)	9.605 (147%)
**Vomiting Status**					
400mgFQ+800mgOZ	Non-Vomiters	70	1324 (60%)	6.374 (102%)	18.66 (73%)
	Vomiters	18	471.1 (146%)	1.352 (257%)	4.978 (199%)
600mgFQ+800mgOZ	Non-Vomiters	70	1209 (47%)	6.29 (92%)	17.42 (63%)
	Vomiters	23	431.2 (136%)	1.243 (242%)	4.421 (183%)
900mgFQ+800mgOZ	Non-Vomiters	71	972.7 (77%)	4.323 (135%)	13.17 (101%)
	Vomiters	22	365.8 (68%)	0.9859 (112%)	3.492 (87%)
1200mgFQ+800mgOZ	Non-Vomiters	69	1034 (71%)	4.155 (119%)	13.05 (86%)
	Vomiters	21	340 (193%)	1.005 (312%)	3.509 (234%)
**Body Weight Band**					
400mgFQ+800mgOZ	7-9.9kg	6	818.2 (68%)	1.985 (141%)	8.277 (97%)
	10-14.9kg	46	1006 (112%)	4.666 (201%)	13.41 (149%)
	15-23.9kg	18	1536 (105%)	6.429 (203%)	20.11 (138%)
	24-34.9kg	2	941.5 (51%)	3.25 (35%)	10.38 (45%)
	over35kg	16	964.2 (34%)	4.558 (69%)	14.63 (46%)
600mgFQ+800mgOZ	7-9.9kg	7	521 (115%)	1.248 (174%)	4.812 (134%)
	10-14.9kg	41	1023 (99%)	4.419 (198%)	13.08 (138%)
	15-23.9kg	26	1024 (92%)	5.374 (173%)	14.56 (127%)
	24-34.9kg	4	1167 (26%)	8.147 (34%)	20.49 (30%)
	over35kg	15	781.4 (54%)	3.583 (94%)	11.09 (77%)
900mgFQ+800mgOZ	7-9.9kg	6	408.2 (103%)	0.8649 (127%)	3.576 (110%)
	10-14.9kg	48	762.5 (104%)	2.913 (187%)	9.147 (143%)
	15-23.9kg	19	947 (91%)	4.8 (202%)	13.65 (148%)
	24-34.9kg	3	713.7 (15%)	3.051 (25%)	10.31 (21%)
	over35kg	17	806.6 (56%)	3.248 (80%)	10.52 (62%)
1200mgFQ+800mgOZ	7-9.9kg	10	312.7 (162%)	0.798 (222%)	3.245 (187%)
	10-14.9kg	36	820.1 (93%)	3.213 (151%)	10.12 (119%)
	15-23.9kg	28	936.1 (154%)	3.615 (251%)	11.41 (185%)
	24-34.9kg	1	703.7	2.445	6.902
	over35kg	15	1043 (38%)	4.263 (91%)	12.94 (56%)
**Region / Age Band**					
400mgFQ+800mgOZ	Africa >=0.5yr & <=2yr	7	984.6 (85%)	2.591 (179%)	10.42 (112%)
	Africa >2yr & <=5yr	62	1125 (113%)	5.099 (204%)	14.93 (150%)
	Africa >5yr	15	907 (34%)	4.425 (72%)	13.32 (51%)
	Asia >5yr	4	1095 (34%)	3.592 (53%)	15.21 (31%)
600mgFQ+800mgOZ	Africa >=0.5yr & <=2yr	8	531.2 (111%)	1.782 (280%)	5.943 (159%)
	Africa >2yr & <=5yr	65	1018 (95%)	4.57 (185%)	13.29 (133%)
	Africa >5yr	15	924.5 (54%)	4.822 (104%)	13.96 (77%)
	Asia >5yr	5	817.7 (73%)	3.859 (97%)	11.67 (92%)
900mgFQ+800mgOZ	Africa >=0.5yr & <=2yr	6	471.2 (71%)	1.159 (93%)	4.526 (75%)
	Africa >2yr & <=5yr	64	824.2 (105%)	3.351 (203%)	10.28 (153%)
	Africa >5yr	17	637.8 (58%)	2.861 (112%)	8.689 (79%)
	Asia >5yr	6	1076 (30%)	3.479 (41%)	13.5 (36%)
1200mgFQ+800mgOZ	Africa >=0.5yr & <=2yr	7	378.1 (146%)	1.145 (162%)	4.182 (149%)
	Africa >2yr & <=5yr	63	802.1 (134%)	3.023 (231%)	9.748 (170%)
	Africa >5yr	15	1007 (31%)	3.96 (48%)	12.24 (37%)
	Asia >5yr	5	1053 (69%)	4.133 (211%)	12.34 (114%)

The reported concentrations are plasma concentrations.
No patients were recruited into the lowest body weight band (5-6.9kg) in this study.
[a] Number of patients with determinable metric;
[b] Geometric Mean (CV%).

Table 4 Selected Summary Statistics of the Individual Ferroquine Blood Exposure Estimates by Treatment Arm for Various Sub-Populations (total n=367).

Treatment Arm	Sub-Group	n[a]	C_{max}[b] [ng/mL]	C_{day7}[b] [ng/mL]	$AUC_{(0-day28)}$[b] [ug*hr/mL]
400mgFQ+800mgOZ	All patients	88	148.1 (52%)	27.08 (51%)	14.92 (40%)
600mgFQ+800mgOZ	All patients	93	222.8 (66%)	40.94 (65%)	22.56 (48%)
900mgFQ+800mgOZ	All patients	93	350 (55%)	63.72 (61%)	33.84 (48%)
1200mgFQ+800mgOZ	All patients	90	467.6 (80%)	87.52 (70%)	46.4 (52%)
**Vomiting Status**					
400mgFQ+800mgOZ	Non-Vomiters	69	157.2 (52%)	28.95 (48%)	15.67 (38%)
	Vomiters	19	119.4 (48%)	21.26 (54%)	12.49 (42%)
600mgFQ+800mgOZ	Non-Vomiters	70	246.9 (53%)	46.6 (54%)	24.87 (42%)
	Vomiters	23	163.2 (88%)	27.61 (74%)	16.76 (50%)
900mgFQ+800mgOZ	Non-Vomiters	69	398.5 (48%)	72.82 (52%)	37.99 (41%)
	Vomiters	24	240.9 (53%)	43.41 (64%)	24.25 (48%)
1200mgFQ+800mgOZ	Non-Vomiters	68	509.3 (82%)	95.72 (73%)	50.63 (52%)
	Vomiters	22	359.3 (66%)	66.38 (51%)	35.43 (40%)
**Body Weight Band**					
400mgFQ+800mgOZ	7-9.9kg	6	129 (28%)	21.67 (36%)	11.76 (26%)
	10-14.9kg	46	163.1 (55%)	27.92 (55%)	15.5 (40%)
	15-23.9kg	18	177.1 (38%)	30.74 (58%)	17.17 (44%)
	24-34.9kg	2	107.6 (11%)	19.24 (34%)	10.54 (20%)
	over35kg	16	100.5 (45%)	24.41 (34%)	13.05 (33%)
600mgFQ+800mgOZ	7-9.9kg	7	256 (71%)	27.12 (84%)	16.69 (50%)
	10-14.9kg	41	227.8 (61%)	45.07 (69%)	24.47 (49%)
	15-23.9kg	26	267.2 (50%)	42.25 (54%)	23.23 (37%)
	24-34.9kg	4	203.1 (92%)	37.47 (93%)	21.23 (69%)
	over35kg	15	147.1 (78%)	36.99 (53%)	20.08 (53%)
900mgFQ+800mgOZ	7-9.9kg	6	311.5 (79%)	36.42 (53%)	23.63 (39%)
	10-14.9kg	48	339.8 (59%)	61.25 (62%)	32.27 (48%)
	15-23.9kg	19	388.9 (50%)	78.73 (69%)	40.12 (56%)
	24-34.9kg	3	354.6 (37%)	58.86 (17%)	30.77 (15%)
	over35kg	17	351.4 (47%)	69.51 (42%)	36.9 (37%)
1200mgFQ+800mgOZ	7-9.9kg	10	428 (94%)	51.39 (85%)	31.45 (42%)
	10-14.9kg	36	462.6 (62%)	91.95 (71%)	48.6 (50%)
	15-23.9kg	28	516.8 (87%)	100.1 (63%)	51.1 (54%)
	24-34.9kg	1	717.7	71.99	38.69
	over35kg	15	410.5 (111%)	87.38 (59%)	45.46 (53%)
**Region / Age Band**					
400mgFQ+800mgOZ	Africa >=0.5yr & <=2yr	7	141.6 (58%)	23.6 (48%)	13.09 (37%)
	Africa >2yr & <=5yr	62	166.9 (49%)	28.96 (54%)	16 (41%)
	Africa >5yr	15	104.5 (45%)	23.06 (41%)	12.59 (36%)
	Asia >5yr	4	93.36 (26%)	22.28 (21%)	12.02 (21%)
600mgFQ+800mgOZ	Africa >=0.5yr & <=2yr	8	259 (63%)	33.07 (107%)	19.62 (69%)
	Africa >2yr & <=5yr	65	242.1 (58%)	43.26 (62%)	23.7 (44%)
	Africa >5yr	15	184.5 (68%)	40.34 (60%)	21.95 (54%)
	Asia >5yr	5	105.2 (92%)	29.38 (41%)	16.14 (41%)
900mgFQ+800mgOZ	Africa >=0.5yr & <=2yr	6	296.2 (54%)	44.77 (49%)	25.67 (22%)
	Africa >2yr & <=5yr	64	358.9 (59%)	65.79 (65%)	34.6 (51%)
	Africa >5yr	17	326 (36%)	64.95 (56%)	34.7 (44%)
	Asia >5yr	6	386.9 (63%)	61.16 (36%)	32.69 (34%)
1200mgFQ+800mgOZ	Africa >=0.5yr & <=2yr	7	352.3 (65%)	67.05 (52%)	35.37 (39%)
	Africa >2yr & <=5yr	63	492.7 (78%)	91.28 (78%)	48.68 (55%)
	Africa >5yr	15	533.6 (60%)	91.42 (41%)	47.28 (38%)
	Asia >5yr	5	242.6 (162%)	65.72 (73%)	35 (64%)

The reported concentrations are blood concentrations.
No patients were recruited into the lowest body weight band (5-6.9kg) in this study.
Patients who vomited after FQ dosing and never received artefenomel were not included in the summaries: 400mg FQ (N=2) or 1200mg (N=1).
[a] number of patients with determinable metric;
[b] Geometric Mean (CV%).

Table 5 Selected Summary Statistics of the Individual SSR97213 Blood Exposure Estimates by Treatment Arm for Various Sub-Populations (total n=367).

Treatment Arm	Sub-Group	n[a]	C_{max}[b] [ng/mL]	C_{day7}[b] [ng/mL]	$AUC_{(0-day28)}$[b] [ug*hr/mL]
400mgFQ+800mgOZ	All patients	88	34.62 (58%)	26.4 (56%)	13.57 (52%)
600mgFQ+800mgOZ	All patients	93	59.65 (78%)	42.83 (76%)	22.37 (66%)
900mgFQ+800mgOZ	All patients	93	94.04 (75%)	68.15 (71%)	35.06 (66%)
1200mgFQ+800mgOZ	All patients	90	140.9 (84%)	98.91 (82%)	50.96 (75%)
**Vomiting Status**					
400mgFQ+800mgOZ	Non-Vomiters	69	38.6 (53%)	28.79 (54%)	14.76 (50%)
	Vomiters	19	23.33 (54%)	19.3 (48%)	10 (44%)
600mgFQ+800mgOZ	Non-Vomiters	70	69.75 (63%)	49.62 (64%)	25.55 (56%)
	Vomiters	23	37.06 (92%)	27.35 (88%)	14.94 (74%)
900mgFQ+800mgOZ	Non-Vomiters	69	115.9 (57%)	81.95 (55%)	41.88 (51%)
	Vomiters	24	51.52 (76%)	40.11 (77%)	21.03 (69%)
1200mgFQ+800mgOZ	Non-Vomiters	68	160.3 (80%)	109.8 (83%)	56.98 (76%)
	Vomiters	22	94.64 (78%)	71.68 (64%)	36.11 (58%)
**Body Weight Band**					
400mgFQ+800mgOZ	7-9.9kg	6	29.08 (28%)	26.17 (31%)	11.85 (30%)
	10-14.9kg	46	36.42 (64%)	27.31 (60%)	13.9 (54%)
	15-23.9kg	18	44.62 (36%)	32.93 (51%)	16.92 (50%)
	24-34.9kg	2	28.25 (8%)	19.77 (21%)	9.704 (20%)
	over35kg	16	24.66 (58%)	19.44 (43%)	10.86 (43%)
600mgFQ+800mgOZ	7-9.9kg	7	46.51 (74%)	33.15 (91%)	16.55 (79%)
	10-14.9kg	41	61.68 (80%)	47.19 (81%)	24.47 (69%)
	15-23.9kg	26	68.28 (66%)	45.21 (64%)	23.14 (53%)
	24-34.9kg	4	54.46 (131%)	32.62 (109%)	17.88 (108%)
	over35kg	15	49.55 (84%)	36.22 (72%)	20.19 (67%)
900mgFQ+800mgOZ	7-9.9kg	6	60.34 (116%)	40.39 (60%)	21.28 (60%)
	10-14.9kg	48	92.05 (79%)	68.03 (77%)	34.03 (68%)
	15-23.9kg	19	107.1 (78%)	82.19 (77%)	41.4 (76%)
	24-34.9kg	3	76.33 (61%)	52.21 (24%)	26.52 (34%)
	over35kg	17	104.8 (47%)	70 (47%)	39.7 (43%)
1200mgFQ+800mgOZ	7-9.9kg	10	114 (99%)	60.09 (86%)	31.99 (73%)
	10-14.9kg	36	133.8 (72%)	105.5 (73%)	52.46 (70%)
	15-23.9kg	28	158.5 (83%)	114.3 (83%)	58.48 (75%)
	24-34.9kg	1	180.6	77.89	42.52
	over35kg	15	145.3 (114%)	91.55 (89%)	50.8 (83%)
**Region / Age Band**					
400mgFQ+800mgOZ	Africa >=0.5yr & <=2yr	7	33.12 (53%)	28.79 (40%)	13.69 (43%)
	Africa >2yr & <=5yr	62	38.58 (56%)	28.91 (57%)	14.69 (53%)
	Africa >5yr	15	24.13 (58%)	18.81 (46%)	10.31 (46%)
	Asia >5yr	4	27.11 (34%)	19.85 (20%)	11.02 (24%)
600mgFQ+800mgOZ	Africa >=0.5yr & <=2yr	8	49.76 (71%)	37.72 (100%)	18.95 (90%)
	Africa >2yr & <=5yr	65	64.21 (75%)	46.08 (74%)	23.77 (62%)
	Africa >5yr	15	56.48 (81%)	38.29 (74%)	21.13 (72%)
	Asia >5yr	5	36.03 (98%)	28.3 (75%)	15.8 (67%)
900mgFQ+800mgOZ	Africa >=0.5yr & <=2yr	6	68.46 (63%)	50.47 (43%)	25.89 (39%)
	Africa >2yr & <=5yr	64	96.43 (85%)	71.46 (79%)	35.92 (72%)
	Africa >5yr	17	95.59 (57%)	64.65 (58%)	35.08 (58%)
	Asia >5yr	6	94.37 (28%)	64.42 (45%)	36.67 (45%)
1200mgFQ+800mgOZ	Africa >=0.5yr & <=2yr	7	95.9 (79%)	73.03 (68%)	37.37 (62%)
	Africa >2yr & <=5yr	63	145.3 (82%)	104.7 (86%)	53.28 (78%)
	Africa >5yr	15	161.3 (71%)	98.79 (53%)	52.44 (55%)
	Asia >5yr	5	110 (171%)	73.94 (145%)	41.24 (116%)

The reported concentrations are blood concentrations.
No patients were recruited into the lowest body weight band (5-6.9kg) in this study.
Patients who vomited after FQ dosing and never received artefenomel were not included in the summaries: 400mg FQ (N=2) or 1200mg (N=1).
[a] number of patients with determinable metric;
[b] Geometric Mean (CV%).

References

[Boulu 2016] Boulu L. POH0456. Population PK analysis of ferroquine (SSR97193), and its metabolite SSR97213 from a pool of phase I and II studies (TDU5419, TDU5967, TDR5969, INT6856, ACT10420, DRI10382, TDU12511 and DRI12805). Sanofi. Internal Report, 2016.

[Macintyre 2017] Macintyre F. et al. A randomised, double-blind clinical phase II trial of the efficacy, safety, tolerability and pharmacokinetics of a single dose combination treatment with artefenomel and piperaquine in adults and children with uncomplicated Plasmodium falciparum malaria. BMC Medicine, 15(1), Oct 2017.
