# Supplementary material for: A randomized, double-blind, phase 2b study to investigate the efficacy, safety, tolerability and pharmacokinetics of a single-dose regimen of ferroquine with artefenomel in adults and children with uncomplicated Plasmodium falciparum malaria
Source: Malar J. 2021 May 19;20:222. doi: 10.1186/s12936-021-03749-4 (PMC8135182; doi:10.1186/s12936-021-03749-4)
Supplement: Supplementary file 4 — Additional file 4. Exposure–response analysis details. Supplementary document including tables and figures to provide further methodological details and results on the exposure–response analysis. [file 12936_2021_3749_MOESM4_ESM.pdf]

S4 Exposure Response Analysis Details

Note. Some of the figures and tables refer to "OZ" which stands for OZ439 and is the same as artefenomel.

Exposure Response Analysis

The relationship between estimated artefenomel and ferroquine C_{day7} and PCR-adjusted ACPR28 (success or failure) was evaluated statistically by logistic regression. This relationship was clearly present in the observed data as shown in the exploratory plot (Figure 9 in the main publication).

All data processing, analysis, model setup and modelling result analysis were conducted within R (Microsoft Open R 3.5.1) combined with the IQR package (v1.1.1) developed by IntiQuan (IQR Tools, https://iqrtools.intiquan.com) to support the entire workflow of a PK/PD analysis from estimations to simulations.

The exposure variable for artefenomel, ferroquine and SSR97213 used in the exposure-response analysis was the C_{day7}. Within the single dose setting of the study the C_{day7} is highly correlated to other possible exposure variables, such as $AUC_{(0-\infty)}$ or C_{day14}. However, C_{day7} was preferred to allow future extrapolation to multi-dose regimens as well for its scientific rationale (concentrations of any given drug may be required to exceed the minimum parasiticidal concentration (MPC) for at least 7 days to achieve full parasite clearance).

The following covariates were considered in the logistic regression analysis:

- Concentrations at Day 7 (C_{day7}) after drug administration for artefenomel, FQ and its metabolite SSR97213. These exposures were estimated as described in the PK analysis supplement. FQ C_{day7} and SSR97213 C_{day7} were included with units of 10ng/mL, to improve the interpretation of the logistic regression results.
- Region (Africa vs. Asia).
- Baseline parasitaemia in parasites/μL (continuous: log transformed, base 10).
- Age of the patient either as continuous variable or as age group: patients ≤5 years vs. patients > 5 years old.
- Sex of the patient
- *Kelch-13* genotype at screening (categorical: WT, ART, OTH or MISSING)*
  - True Wild Type (WT; no mutation in the locus)
  - Artemisinin resistance associated mutations: Validated [N458Y, Y493H, R539T, I543T, C580Y] and candidate [P441L, F446I, G339A, P553L, V568G, P574L, A675V] (ART; as per table 1 of the WHO Report, April 2017 [WHO 2017])
  - Other mutations (OTH; any synonymous or non-synonymous mutations not captured in ART)
- Center Grouping (categorical: Africa Flagged, Africa, Asia). A few African study centers were identified to have had data quality issues that may have impacted the efficacy endpoints: These were flagged.

* Post-analysis notes on the categories for *kelch-13* genotype used in for the analysis:

- Candidate mutation G339A (ART) was a typo and should have been G449A. Neither was in the data set. No impact on the analysis.
- A newer list of candidate and validated resistance mutations is available [WHO 2018]
  - F446I and P553L, previously candidates, are now validated, but category not changed (ART). No impact on the analysis.
  - M476I and R561H are new validated. Not associated in previous report. Neither was part of the data set. No impact on the analysis.
  - C469F, A481V, P527H, N537I, G538V and F673I are new candidates. Not associated in previous report. None were part of the data set. No impact on the analysis.

Continuous covariates were centered at approximately their median value in the analysis to allow a better estimation of the model intercept: C_{day7} artefenomel at 6 ng/ml, C_{day7} FQ and SSR97213 at 60 ng/ml, Age at 5 years, and log baseline parasitaemia at 4.

The general form of the logistic regression model can be presented as follows,

$$log(odds) = \beta_0 + \sum_{i=1}^{n} \beta_i * cov_i + \sum_{i=1}^{n-1} \sum_{j=i+1}^{n} \beta_{i,j} * cov_i * cov_j$$

where

$$log(odds) = logit(p) = log\left(\frac{p}{1-p}\right)$$

where p is the probability of $ACPR_{28}$ response, β_0 is the intercept, β_i represents the slope for the effect of covariate (cov) i, and $\beta_{i,j}$ is the coefficient for the interaction between two covariates i and j.
For the logistic regression the function glm() in R, as implemented in IQRtools, was used:
e.g. glm(ACPR28 ~ cov1 + cov2, family = binomial(link = "logit"), data=thedat)

The Odds Ratio (OR) for each covariate was subsequently calculated by taking the exponent of the estimated slope: *e.g.* Odds Ratio for COV1 = $e^{(\beta_1)}$.

A base model was developed first, including the artefenomel C_{day7}, FQ C_{day7}, as well as the baseline parasitaemia, as the basis for evaluation of any other covariate effects. SSR97213 C_{day7} was not included in the base model, since it was highly correlated with FQ C_{day7} (but it was still included in the univariate analysis).

The remaining covariates were evaluated as follows:

1. A univariate analysis (compared to the base model) of each of the other covariates to be evaluated. Identification of any statistically significant (p-value <0.05 and >2 point lower Akaike Information Criterion [AIC]) covariates as well as covariates deemed scientifically credible or of

particular interest (forward selection). If two covariates described the same variable, such as age and age group, only the one with lowest AIC was retained.

2. All identified covariates were included in a new full model. Removal of each covariate was evaluated. If removal of one or more covariates did not make the model significantly worse (increase in AIC of >2 points) the one covariate whose removal was associated with the lowest AIC was removed. This process was repeated until no covariate could be removed without resulting in a significantly worse (increase in AIC of >2 points) model (backwards elimination).
3. Evaluation of any interactions between the remaining covariates in the model. AIC and other statistical criteria as well as scientific judgment were applied.

A visual predictive check for the final model was performed by plotting the observed and model predicted $ACPR_{28}$ by treatment arm for the study population with 90% CI in various Sub-Groups. The simulations were based on the actual patient population in each Sub-Group (*i.e.* estimated individual C_{day7} and covariates in those patients) and summarized across 1000 replicates.

Analysis data set:

Table 1. Summary of Categorical Variables in the Exposure - Response Data Set

Covariate	Category	400mgFQ+ 800mgOZ439 (N=67)	600mgFQ+ 800mgOZ439 (N=77)	900mgFQ+ 800mgOZ439 (N=76)	1200mgFQ+ 800mgOZ439 (N=78)	TOTAL (N=298)
Sex	Female	22 (32.8%)	40 (51.9%)	34 (44.7%)	48 (61.5%)	144 (48.3%)
	Male	45 (67.2%)	37 (48.1%)	42 (55.3%)	30 (38.5%)	154 (51.7%)
Region	Africa	63 (94%)	73 (94.8%)	71 (93.4%)	73 (93.6%)	280 (94%)
	Asia	4 (5.97%)	4 (5.19%)	5 (6.58%)	5 (6.41%)	18 (6.04%)
Center ID	CREC	3 (4.48%)	4 (5.19%)	2 (2.63%)	3 (3.85%)	12 (4.03%)
	USS-Libreville	1 (1.49%)	1 (1.3%)	1 (1.32%)	3 (3.85%)	6 (2.01%)
	CERMEL	10 (14.9%)	11 (14.3%)	8 (10.5%)	12 (15.4%)	41 (13.8%)
	KEMRI-Kisumu	7 (10.4%)	6 (7.79%)	4 (5.26%)	8 (10.3%)	25 (8.39%)
	KEMRI-Kondele	0 (0%)	0 (0%)	0 (0%)	0 (0%)	0 (0%)
	CITSC	9 (13.4%)	7 (9.09%)	10 (13.2%)	7 (8.97%)	33 (11.1%)
	DH-BuDang	3 (4.48%)	3 (3.9%)	4 (5.26%)	3 (3.85%)	13 (4.36%)
	DH-PhuThien	1 (1.49%)	1 (1.3%)	1 (1.32%)	2 (2.56%)	5 (1.68%)
	IDRC-Tororo	15 (22.4%)	20 (26%)	23 (30.3%)	19 (24.4%)	77 (25.8%)
	IRSS	9 (13.4%)	14 (18.2%)	13 (17.1%)	11 (14.1%)	47 (15.8%)
	CNRFP-Banfora	9 (13.4%)	10 (13%)	10 (13.2%)	10 (12.8%)	39 (13.1%)
	CNRFP-Niangoloko	0 (0%)	0 (0%)	0 (0%)	0 (0%)	0 (0%)
Kelch-13 Genotype at Screening	WT	51 (76.1%)	62 (80.5%)	63 (82.9%)	61 (78.2%)	237 (79.5%)
	MISSING	11 (16.4%)	7 (9.09%)	9 (11.8%)	10 (12.8%)	37 (12.4%)
	OTH	1 (1.49%)	4 (5.19%)	1 (1.32%)	2 (2.56%)	8 (2.68%)
	ART	4 (5.97%)	4 (5.19%)	3 (3.95%)	5 (6.41%)	16 (5.37%)
Age Group	>5-year-old	16 (23.9%)	15 (19.5%)	18 (23.7%)	18 (23.1%)	67 (22.5%)
	<=5-year-old	51 (76.1%)	62 (80.5%)	58 (76.3%)	60 (76.9%)	231 (77.5%)
Center Grouping	Africa	49 (73.1%)	57 (74%)	60 (78.9%)	55 (70.5%)	221 (74.2%)
	Africa Flagged	14 (20.9%)	16 (20.8%)	11 (14.5%)	18 (23.1%)	59 (19.8%)
	Asia	4 (5.97%)	4 (5.19%)	5 (6.58%)	5 (6.41%)	18 (6.04%)
PCR-Adjusted $ACPR_{28}$	non-cured	18 (26.9%)	13 (16.9%)	10 (13.2%)	10 (12.8%)	51 (17.1%)
	cured	49 (73.1%)	64 (83.1%)	66 (86.8%)	68 (87.2%)	247 (82.9%)

N: Number of patients

Number of patients in each category and percentage within this category

Africa Flagged. Grouping of the following three Study Centers: USS-Libreville, CERC and CERMEL

Kelch-13 Genotype at Screening. WT=True wild type, ART=Artemisinin resistance associated mutations, OTH=Other mutations currently not associated with Artemisinin resistance, MISSING=No genotype available

Table 2. Summary of Continuous Variables in the Exposure - Response Data Set

Covariate	400mgFQ+ 800mgOZ439 (N=67)	600mgFQ+ 800mgOZ439 (N=77)	900mgFQ+ 800mgOZ439 (N=76)	1200mgFQ+ 800mgOZ439 (N=78)	TOTAL (N=298)
Age (years)	8.04 (11.1) [1.2-56]	6.57 (8.91) [0.728-61.9]	7.23 (9.05) [0.854-55.8]	7.44 (10) [0.635-53.4]	7.3 (9.71) [0.635-61.9]
FQ C_{Day7} (ng/mL)	31.8 (16.3) [7.04-83.6]	49.1 (24.7) [7.36-125]	70.3 (37.1) [14.7-173]	106 (57.1) [10.5-293]	65.5 (46.6) [7.04-293]
SSR97213 C_{Day7} (ng/mL)	30.7 (16) [5.02-94.1]	53.3 (30.8) [7.19-174]	79 (46.4) [11.6-221]	124 (77.1) [8.07-463]	73.4 (59.9) [5.02-463]
OZ439 C_{Day7} (ng/mL)	7.35 (6.06) [0.0704-28.9]	6.72 (5.21) [0.0519-21.5]	4.94 (5.03) [0.208-21.8]	5.17 (4.51) [0.0607-19]	6 (5.27) [0.0519-28.9]
Baseline Parasitaemia Asexual (log_{10}) (log_{10}(p/uL))	4.17 (0.73) [1.26-5.26]	4.36 (0.626) [2.74-5.25]	4.3 (0.58) [2.78-5.09]	4.18 (0.625) [2.96-5.02]	4.26 (0.641) [1.26-5.26]
Baseline Parasitaemia Asexual (p/uL)	34400 (34600) [18-183000]	45300 (42000) [550-178000]	36300 (32200) [599-124000]	32000 (32000) [920-106000]	37100 (35700) [18-183000]

N: Number of patients
Entries represent: Mean (Standard deviation) [Minimum-Maximum]

The correlations between the various covariates were explored. Apart from the obvious correlations between covariates, such as region and center grouping, of importance was the correlation between *kelch-13* genotype at screening and region: All ART genotypes were in Asia (Vietnam), only 2 out of the 19 Asian patients had a WT genotype, and no ART genotype was found in any African patient. It was therefore not possible to distinguish between Asia and *kelch-13* genotype as factor. Region as covariate was preferred.
Also, a negative correlation between age and baseline parasitaemia was observed (*i.e* higher baseline parasitaemias were mostly observed in the youngest patients). In the model building, preference was given to baseline parasitaemia over age as a covariate if needed.
In addition, a strong correlation was found between the C_{day7} of FQ and SSR97213. Therefore, the C_{day7} of FQ was considered as a proxy for the combined parent metabolite exposure.

Figure 1. Results - Univariate Addition to the Base Model

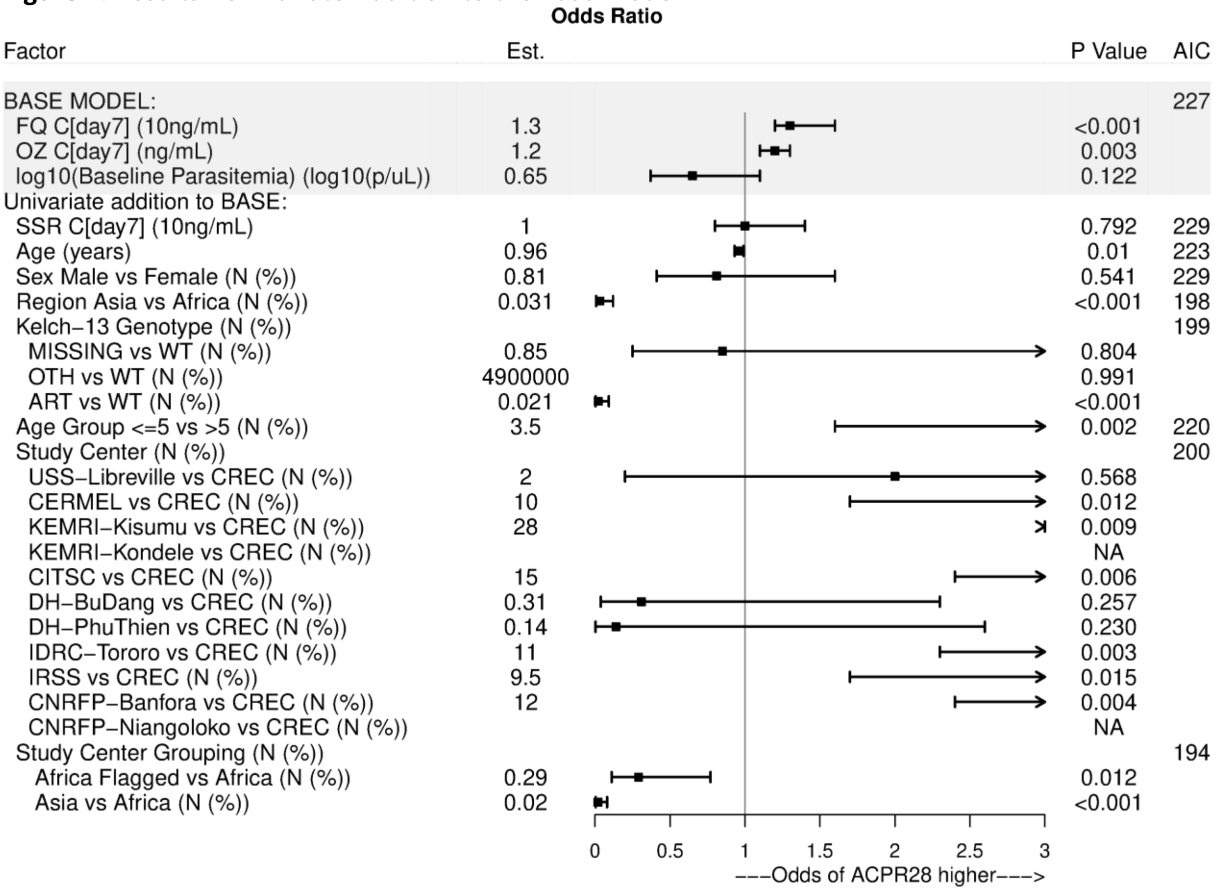

Based on the univariate analysis, the following covariates were included in the full model: OZ439 C_{day7}, FQ C_{day7}, baseline parasitaemia, age group and study center grouping.

During the backward elimination only age group could be removed without making the model worse. No meaningful interactions were identified for the final model.

Table 3. Final Logistic Regression Model Parameter Estimates

Parameter	Value	95% CI	P.value
Intercept	3.21	[2.491 , 4.073]	1.22e-15
$(FQ_{Day7} - 60) / 10$	0.3388	[0.188 , 0.5115]	3.892e-05
$(OZ_{Day7} - 6)$	0.1494	[0.04568, 0.2732]	0.009591
($\log_{10}$ Baseline Parasitaemia - 4)	-1.04	[-1.815 , -0.357]	0.004983
Study Center Group = Africa Flagged	-1.228	[-2.202 , -0.2668]	0.01215
Study Center Group = Asia	-3.918	[-5.556 , -2.527]	2.778e-07

Model: $ACPR_{28}$ ~ $(FQ_{Day7}$-60)/10 + $(OZ_{Day7}$-6) + (log10BPar-4) + SCGRP
Implemented as: fit <- glm($ACPR_{28}$ ~ FQ_{Day7} + OZ_{Day7} + log10BPar + SCGRP, data, family=binomial, method = glm.fit)
Reference category for SCGRP: Africa

The model predictions and observed $ACPR_{28}$ results for various sub populations of interest are shown in Figure 2. This figure shows that the model predicts the observed $ACPR_{28}$ well.

Figure 2. Model-predicted vs Observed PCR-adjusted ACPR$_{28}$ in Various Sub-Populations.

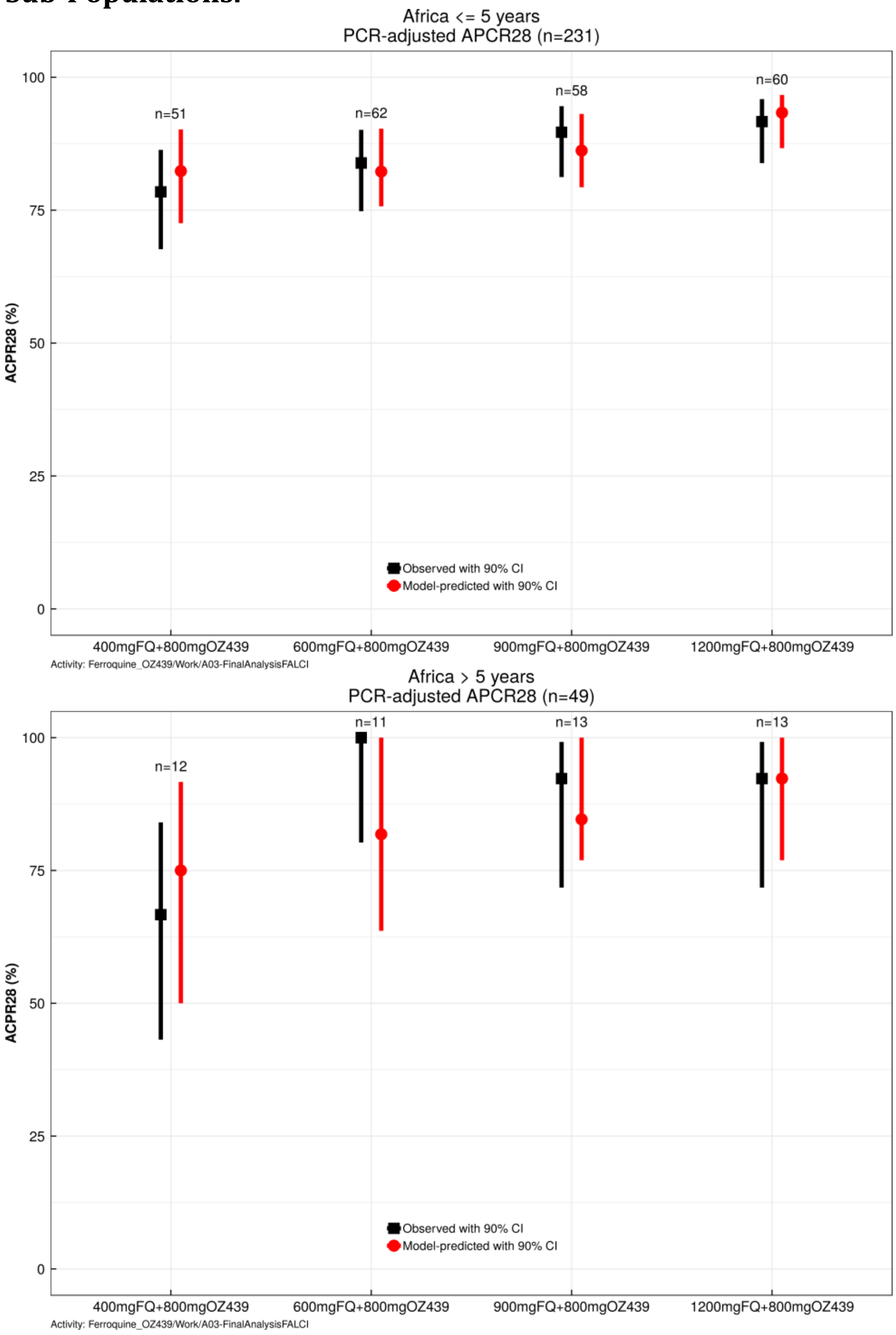

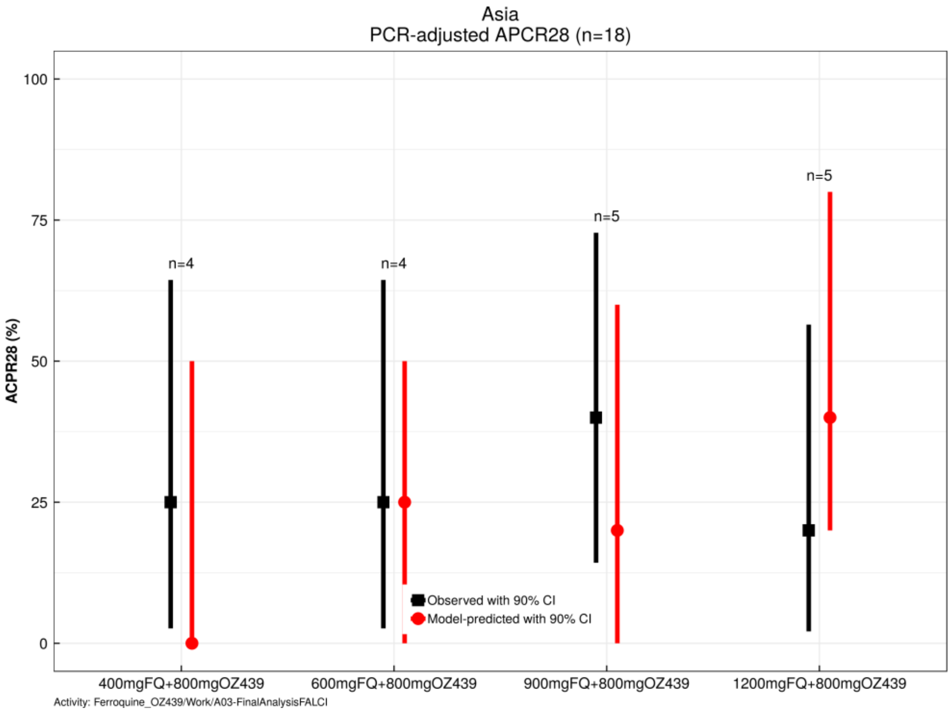

[WHO 2017] WHO. Global Malaria Programme. Artemisinin and artemisinin-based combination therapy resistance. Status report. World Health Organization Publication, April 2017.

[WHO 2018] WHO. Artemisinin resistance and artemisinin-based combination therapy efficacy - Global Malaria Programme, August 2018 Status Report. World Health Organization Publication, Geneva. https://www.who.int/malaria/publications/atoz/artemisinin-resistance-august2018/en/.
