# Supplementary material for: A randomized, double-blind, phase 2b study to investigate the efficacy, safety, tolerability and pharmacokinetics of a single-dose regimen of ferroquine with artefenomel in adults and children with uncomplicated Plasmodium falciparum malaria
Source: Malar J. 2021 May 19;20:222. doi: 10.1186/s12936-021-03749-4 (PMC8135182; doi:10.1186/s12936-021-03749-4)
Supplement: Supplementary file 5 — Additional file 5. Electrocardiogram (ECG) exposure–response analysis details. Supplementary document including tables and figures to provide further methodological details and results on the ECG exposure–response analysis. [file 12936_2021_3749_MOESM5_ESM.pdf]

S5 Electrocardiogram (ECG) exposure-response analysis details

Design of the ECG exposure-response analysis

The cardiac safety of the participating patients was monitored using 12-lead ECG recording. The following quantitative ECG parameters were determined using centralized readings of all digital ECG recordings (triplicate) by independent experts: heart rate (HR), QRS duration, RR interval, PR interval, QT interval, corrected QT interval using Fridericia's formula (QTcF), and corrected QT interval using Bazett's formula (QTcB).

Furthermore, blood and plasma samples were collected to measure the concentrations of ferroquine (FQ), its active metabolite SSR97213 and artefenomel (OZ439). The time span used in the ECG exposure-response analyses was limited to the time span of ECG recordings in the study, i.e., from pre-dose to 48 hours post-dose.

Using different types of plots, the relationship between change from baseline in HR, PR interval, QRS duration, QTcB, and QTcF, and the total concentration of FQ + SSR97213 and OZ439 concentrations was explored graphically, in order to investigate any potential delayed or sustained effects and the type of modelling to be done. Subsequently, a random coefficients linear regression of HR, PR, QRS, QTcB, and QTcF change from baseline versus concentration was performed, with fixed terms for common intercept and slope, and with random terms for subject-specific intercept and slope using a SAS® PROC MIXED procedure.

Population included in the ECG exposure-response analysis

A total of 366 subjects, 20 Asian and 346 African subjects, were included in the analysis. Most subjects were ≤14 years (305 versus 61 subjects >14 years) and most of these were in the age group of >2 to ≤5 years.

Adequacy of QT correction methods

A graphical exploration was performed to assess the appropriateness of QT correction methods by plotting baseline individual QTc values on the y-axis and baseline HR values on the x-axis (Figure 1). These scatter plots clearly indicated that Bazett's method provided a better correction of the QT interval when compared to Fridericia's method and, therefore, QTcB was used for the exposure-response analyses.

Figure 1 Scatter plot of QTcF (left panel) and QTcB (right panel) values versus HR values at baseline

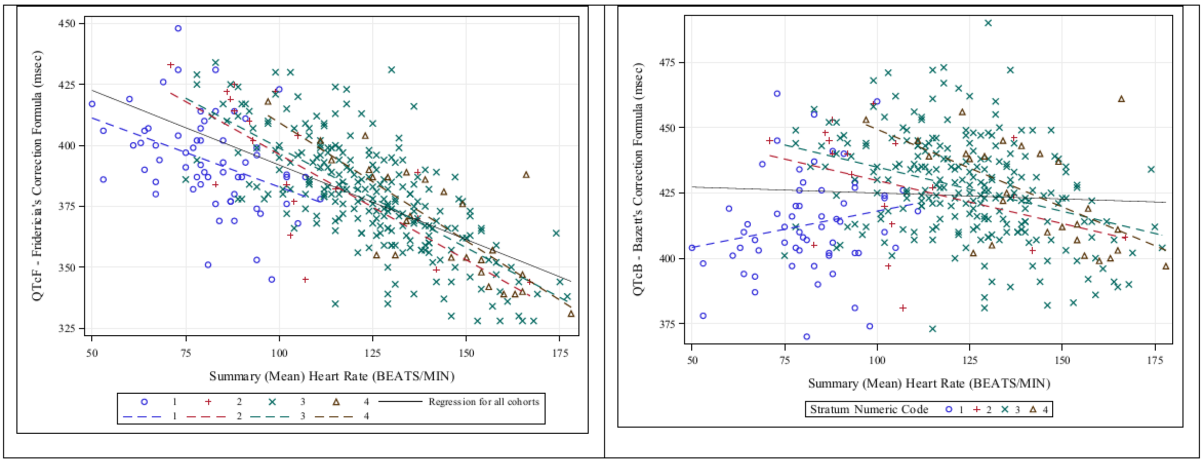

Stratum 1: >14 years and <70 years (cohort 1); Stratum 2: >5 years and ≤14 years (cohort 2), Stratum 3: >2 years and ≤5 years (cohort 3); Stratum 4: >6 months and ≤2 years (cohort 4).

Results of the ECG exposure-response analysis

A significant correlation between the concentrations of FQ and its metabolite SSR97213 was observed. This precluded the ability to dissociate the effects of FQ and SSR97213 on the ECG and meant that the main concentration-response analysis was performed using the total concentrations of FQ + SSR97213 rather than with the concentrations of each compound separately as covariates in the model. Of note, FQ and SSR97213 concentrations were also significantly correlated to OZ439 concentrations, but these correlations were much less strong.

Following administration of study treatments, HR decreased but this decrease did not appear to be related to the concentration of FQ + SSR97213 nor to the concentration of OZ439. For both PR interval and QRS duration, a significant linear concentration-response relationship was observed. However, the observed effects were small, ie, ≤5.6 ms at the geometric mean C_{max} of FQ + SSR97213 at the highest dose for PR interval and ≤2.8 ms at the geometric mean C_{max} for QRS duration. These small effects are unlikely to be of clinical relevance. Scatter plot of changes in QTcB versus FQ+SSR97213 and OZ439 concentrations are provided in Figure 2 and Figure 3, respectively.

The concentration-response relationship between FQ+SSR97213 and QTcB could be best described by a non-linear E_{max} model. The model predicted changes in QTcB at the C_{max} of FQ + SSR97213 varied from 7.0 ms (90% CI 5.8 to 8.2) at a dose of 400 mg FQ to 15.4 ms (90% CI 13.9 to 16.9) at a dose of 1200 mg FQ. At doses of 600 mg FQ and higher, the upper limit of the 90% CI exceeded 10 ms. Of note, with the exposure to FQ + SSR97213 obtained in the different treatment arms, the plateau of effect was not reached since the largest C_{max} was close to the concentration providing half of the maximum effect (EC_{50}). Due to the high correlation of FQ and SSR97213 concentrations, the individual contribution of FQ and its metabolite on the QTcB prolongation could not be established.

When OZ439 concentration was added to the model, the estimated maximum effect on QTcB decreased from 34.7 ms (95% CI 23.8 to 45.7) to 21.2 ms (95% CI 12.2 to 30.3) whereas an increase of the effect on QTcB at the two lowest doses of FQ was observed. There is no clear explanation for this.

Figure 2 Individual changes from baseline in QTcB versus total (FQ+SSR97213) concentrations

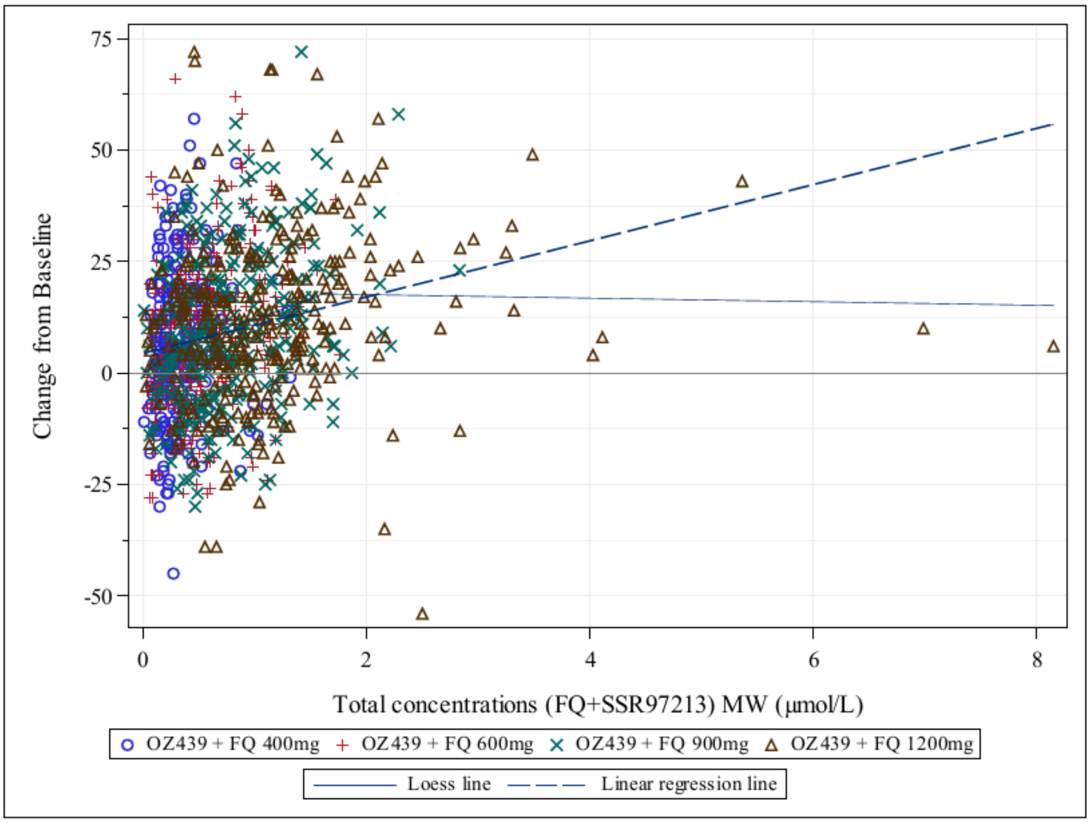

Figure 3 Individual changes from baseline in QTcB versus OZ439 concentrations

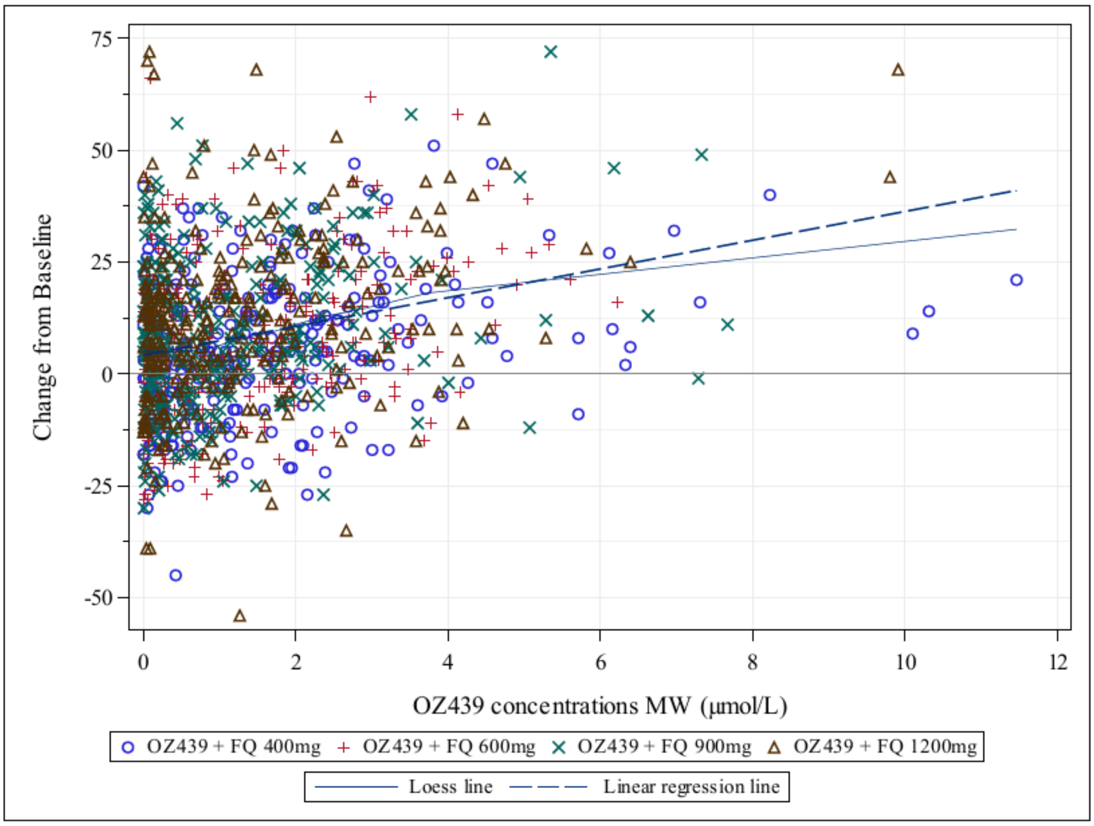

Conclusion

The results of ECG concentration-response analyses indicated that administration of different doses of FQ associated with a fixed dose of OZ439 caused small increases in PR interval and QRS duration that are unlikely to be of clinical relevance. A graphical exploration to assess appropriateness of QT exploration methods showed that Bazett's method provided a better correction of the QT interval when compared to Fridericia's method and, therefore, QTcB was used for the exposure-response analyses. The clinical relevance of the observed increase in QTcB in the concentration-response analyses remains to be established. A significant limitation of the study in evaluating the ECG exposure-response was the lack of a placebo control group.
